# Polyethylenimine–Bisphosphonate–Cyclodextrin Ternary Conjugates: Supramolecular Systems for the Delivery of Antineoplastic Drugs

**DOI:** 10.1021/acs.jmedchem.1c00887

**Published:** 2021-08-09

**Authors:** Simona Plesselova, Pablo Garcia-Cerezo, Victor Blanco, Francisco J. Reche-Perez, Fernando Hernandez-Mateo, Francisco Santoyo-Gonzalez, María Dolores Giron-Gonzalez, Rafael Salto-Gonzalez

**Affiliations:** † Department of Biochemistry and Molecular Biology II, School of Pharmacy, University of Granada, E-18071 Granada, Spain; ‡ Department of Organic Chemistry, School of Sciences, University of Granada, E-18071 Granada, Spain; § Biotechnology Institute, University of Granada, E-18071 Granada, Spain; ∥ Unit of Excellence in Chemistry Applied to Biomedicine and the Environment of the University of Granada, E-18071 Granada, Spain

## Abstract

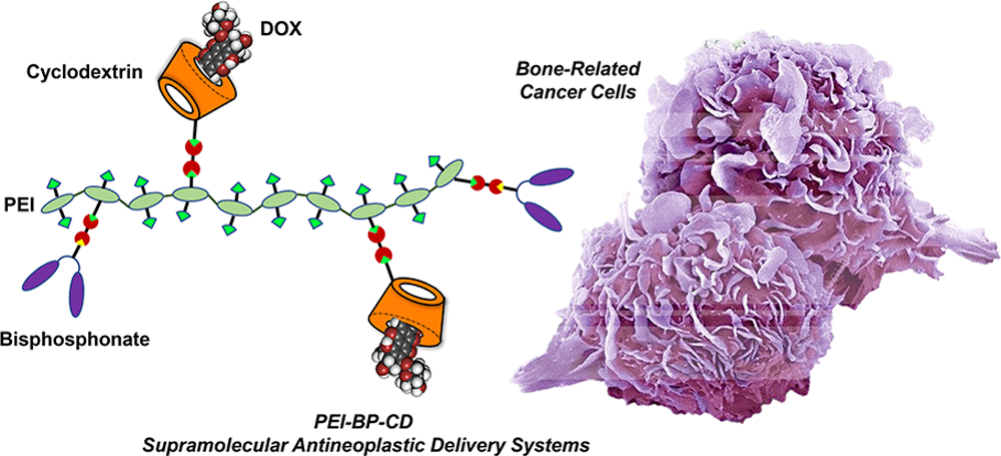

Bisphosphonates (BPs) are bone-binding molecules that provide targeting capabilities to bone cancer cells when conjugated with drug-carrying polymers. This work reports the design, synthesis, and biological evaluation of polyethyleneimine–BP–cyclodextrin (PEI-BP-CD) ternary conjugates with supramolecular capabilities for the loading of antineoplastic drugs. A straightforward, modular, and versatile strategy based on the click aza-Michael addition reaction of vinyl sulfones (VSs) allows the grafting of BPs targeting ligands and βCD carrier appendages to the PEI polymeric scaffold. The *in vitro* evaluation (cytotoxicity, cellular uptake, internalization routes, and subcellular distribution) for the ternary conjugates and their doxorubicin inclusion complexes in different bone-related cancer cell lines (MC3T3-E1 osteoblasts, MG-63 sarcoma cells, and MDA-MB-231 breast cancer cells) confirmed specificity, mitochondrial targeting, and overall capability to mediate a targeted drug transport to those cells. The *in vivo* evaluation using xenografts of MG-63 and MDA-MB-231 cells on mice also confirmed the targeting of the conjugates.

## Introduction

**Figure 1:**
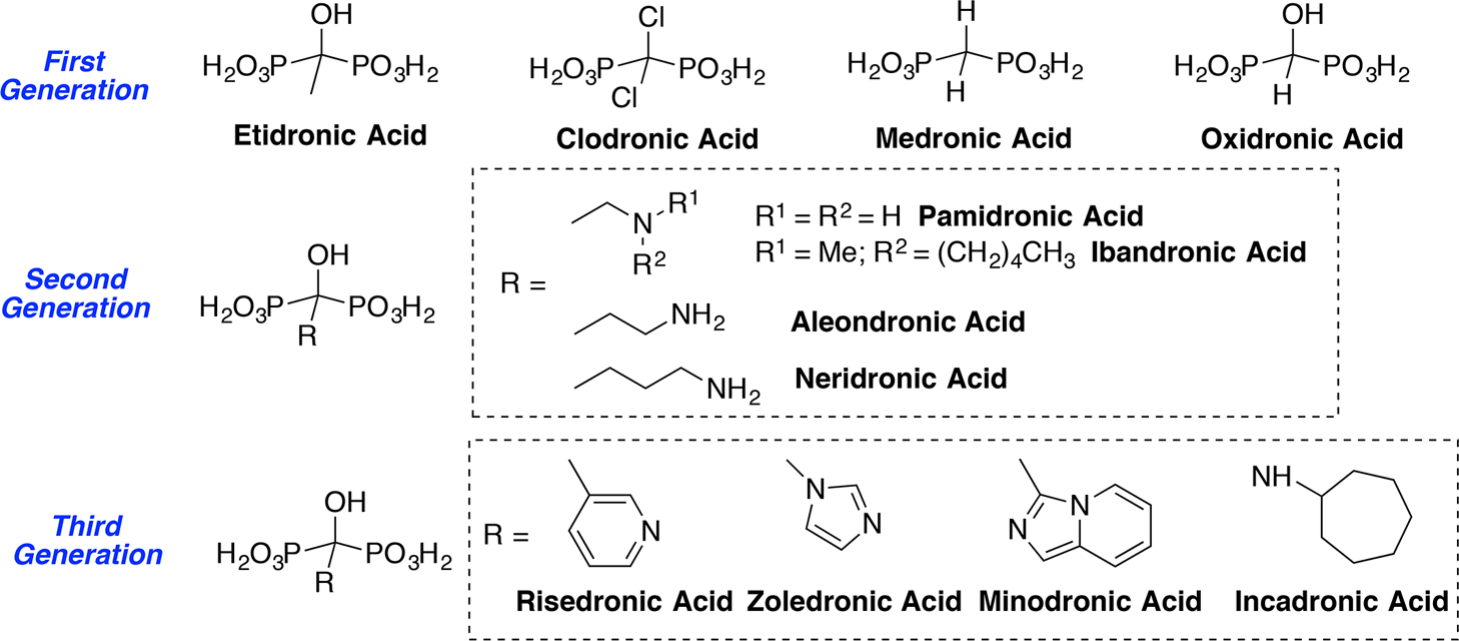
Structures of the different– generations of bisphosphonates in their acid forms.

Targeting therapeutic agents to bone using bisphosphonates (BPs) is an attractive technology widely explored since the 1990s to treat bone diseases, including osteoporosis, bone metastases, multiple myeloma, or osteosarcoma.^2^ This targeting methodology represents an intriguing solution to side effects and to the lack of selectivity associated with conventional therapies. This is especially relevant in the case of antineoplastic drugs for bone cancer and metastases. By virtue of their ability to bind to Ca^2+^, BPs behave as hydroxyapatite (HA) ligands^3^ and drug-delivery systems incorporating conjugated BPs become osteotropic (bone-seeking) nanocarriers,^4^ leading to an optimization of the therapeutic index. Devoted efforts have led to the development of successive generations of BPs (first, a non-nitrogen generation, and later on, second and third nitrogen-containing generations) with improved therapeutic effectiveness (Figure 1).^5^ Associated to the well-defined bone-binding properties of BPs, their preferential uptake by osteoclasts, antiresorptive effects, and relative safety of BP therapies are the bases of multiple successful therapeutic applications.^5−7^

Despite these significant advancements, the optimal design of the widely used BP–drug covalent conjugates is a task not exempt of limitations.^8^ First, optimum conjugation strategies usually require a compound-to-compound approach. Moreover, BP conjugation can alter the intrinsic pharmacological activity of the conjugated drug due to the alteration of biologically relevant reactive functional groups of the drug (amino, hydroxyl, or carboxyl groups) during the BP-drug linking. On the other hand, the pharmacological and pharmacokinetic properties of the BPs can also be affected by the conjugation. Finally, optimal BP-drug linkers, including degradable linkers, have to be inserted to attain an adequate balance of high stability in the blood stream and instability in the bone compartment to allow delivery and release of the parent drug. Therefore, the effort to develop novel osteotropic systems for the bone delivery of antineoplastic drugs continues to be of interest.

A rational design using supramolecular chemistry represents an attractive option with multiple benefits.^9^ Using specific, dynamic, and tunable noncovalent interactions, engineered approaches to supramolecular drug delivery allow improved routes for the incorporation and targeting of drugs. The use of supramolecular motifs gives rise to carriers with a controlled encapsulation and release of therapeutics that preserve their integrity, preventing the chemical modifications associated with covalent conjugation. Furthermore, the modularity of supramolecular interactions also facilitates the opportunity to combine multiple drugs within one delivery platform and the incorporation of several targeting units. In fact, the design opportunities afforded by supramolecular chemistry have already been explored for BP-based osteotropic drug-delivery systems, giving rise to a panoply of architectures: hydrogels, liposomes, bioceramics, nanocapsules, and nanospheres, among others.^10−12^

Cyclodextrins (CDs),^13,14^ one of the most recognizable macrocyclic supramolecular motifs, can be used for the development of osteotropic drug-delivery systems, which, to the best of our knowledge, have not yet been described. We have herein described a novel design of nanocarriers based on polyethylenimine (PEI)^15^ as a polymeric scaffold for the simultaneous conjugation of BPs as targeting ligands and CDs as supramolecular drug carriers. Polymers represent an attractive platform for engineering multifunctional systems, due to their high density of functional groups.^16^ Although bone-targeted polymer therapeutics is a prolific research area,^17−19^ nowadays, relatively few BP-based bone-targeted antineoplastic agents delivered by polymers have been explored. Furthermore, in the reported cases, both the ligand moieties and the anticancer payloads have been covalently grafted to the polymeric backbone.^12,20−22^ In contrast, our approach is based on robust chemistry, the click Michael addition reaction to vinyl sulfones (VSs),^23−25^ a modular assembly for accessing PEI-BP-CD ternary conjugates, and the supramolecular loading by hosting guest antineoplastics in the CD cavity. The therapeutics are conceived as open systems that can be tailored ad hoc through the modification of both the BP ligand and the CD-drug tandem.

To validate our design and the usefulness of the novel targeting anticancer agents, biological assays were carried out in cell cultures of MC3T3-E1 osteoblasts, MG-63 sarcoma cells, and MDA-MB-231 breast cancer cells. These studies were extended to animal models of xenografts of those cells using doxorubicin (DOX), as a model antitumoral.

## Results and Discussion

### Rational Design and Chemistry

To develop innovative and efficient bone-related therapeutics, macromolecular polymer–BP–CD ternary conjugates were designed by combining two already known polymer-based binary systems, namely, BP–polymer conjugates and CD–appended polymers. Using this approach was expected to result in a synergism between the active targeting ability of BPs with the formation of supramolecular CD–host drug inclusion complexes in a modular and versatile bone-targeted drug-delivery system.

PEI was selected as a suitable polymeric scaffold. Although PEI is a frequently used targeted nanocarrier, most of its applications deal with gene delivery, to improve the efficiency of gene transfer.^26^ More recently, a PEI-mediated gene and drug codelivery has been reported.^27^ However, forms of PEI-based bone-seeking nanoplatforms functionalized with BP are scarce in the literature and the reported advances have focused on the development of radiopharmaceuticals.^28−30^ PEI is attractive due to its intrinsic properties: defined size and molecular weight, high solubility in various solvents, including water, and, more remarkably, a high density of amine functional groups that are available for covalent chemical conjugation. Furthermore, PEI exhibits an inherent electrostatic attraction toward HA due to the complementary ionic character of both compounds, since HA shows a slightly negative surface charge under physiological conditions.^31^ Among the commercially available PEI, 2 kDa PEI was chosen for our objectives. Numerous studies have shown that the molecular weight of PEI is a determining parameter of its cytotoxicity, observing that cytotoxicity increases with polymer size. In contrast, low-molecular-weight 2 kDa PEI has proven to be nontoxic *in vivo*, providing long-term safety.^32^ Therefore, the polyamine nature of PEI is a favorable structural factor in increasing the binding affinity of the novel PEI-BP-CD ternary conjugates to the mineral surface. Second-generation nitrogen-containing BPs (alendronate, ALN, as the most prominent) are known to show strong affinities to the HA surface due to the presence of positively charged amine moieties in its side chain that allows the formation of additional interactions with HAp through the formation of N–H–O hydrogen bonds.^33^

**Figure 2:**
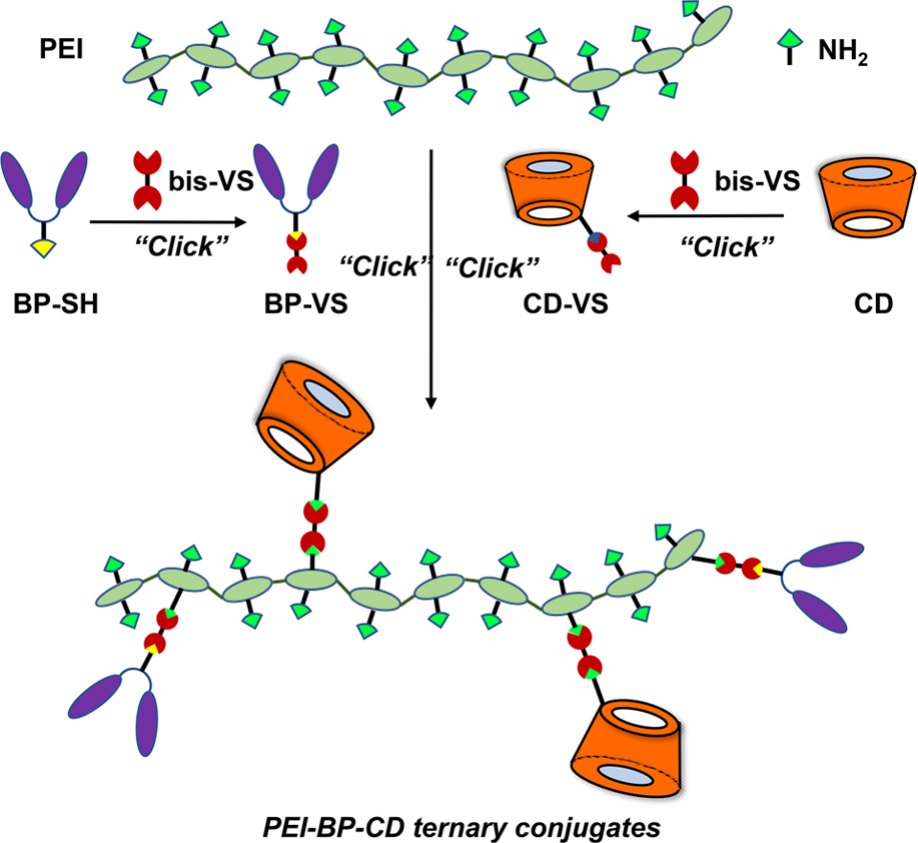
Rational design for the synthesis of PEI-BP-CD ternary conjugates (PEI = polyethylenimine, BP = bisphosphonate, CD = cyclodextrin, and VS = vinyl sulfone).

To obtain spatiotemporal drug release to bone-related cells, the covalent modular grafting of PEI was planned, with a BP as a target agent and a CD as a drug carrier (Figure 2). To this end, the click aza-Michael addition reaction to VSs of the amino groups of PEI was adopted as optimal conjugation chemistry. The strength and simplicity of the conjugate addition reactions to VSs have made it possible to make significant outputs in multiple (bio)conjugation applications.^23−25^

In our case, VS functionalization of both the BP and the CD is required prior to the conjugation of PEI (Figure 2). In this endeavor, a similar approach was undertaken for both compounds: a Michael-type addition reaction of a bis-VS (divinylsulfone, DVS, or 1,2-bis(2-ethenylsulfonylethoxy)ethane, DVS-EEE) with a suitable nucleophilic derivative of a BP and a CD and ulterior aza-Michael addition of the obtained BP-VS and CD-VS derivatives. In this strategy, the bis-VS plays the role of homobifunctional cross-linker to connect the functional moieties (BP and CD) with the polymeric backbone (PEI). Moreover, the structure of the bis-VS allows modulation of the length of the spacer between the BP and the polymer scaffold if required. In this manner, the strategy used is not only modular but also flexible. These characteristics make it possible to attain ad hoc structural variability and functionality by the adequate selection of the BP targeting a linker and the size of the host hydrophobic cavity of the CD to best fit the loaded drug guest.

**Scheme 1:**
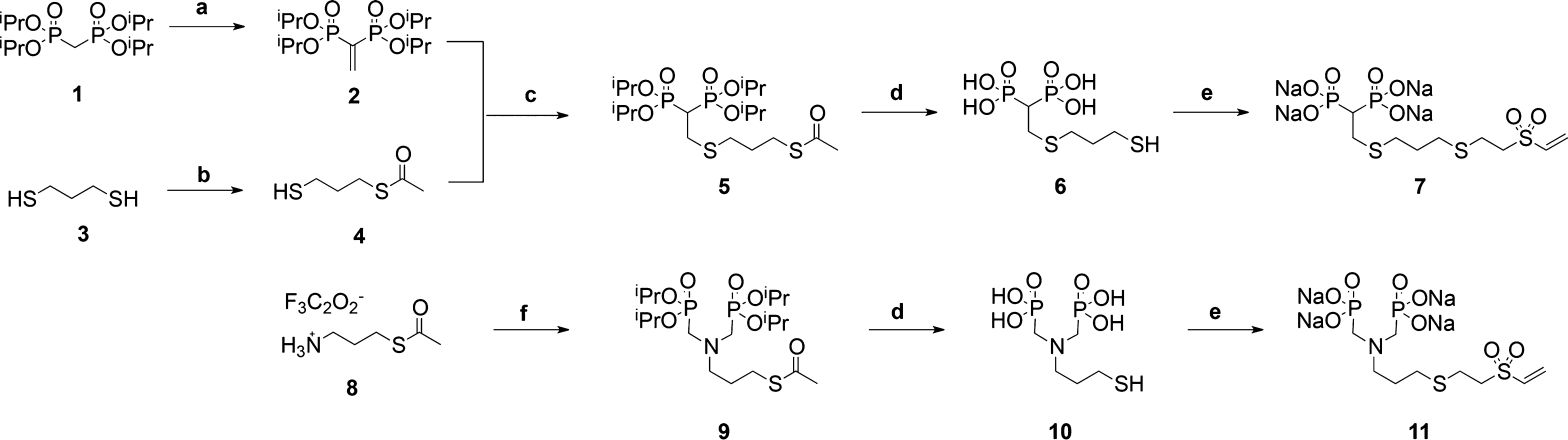
Synthesis of Bisphosphonate Vinyl Sulfone (BP-VS) Derivatives (**7** and **11**) Reagents and conditions: (a) paraformaldehyde, MeOH, reflux, 6 days, then *p*-TsOH, toluene, reflux, 40 h, yield = 74% for the two steps; (b) CH_2_Cl_2_/pyridine, acetic anhydride, rt, 16 h, yield = 46%; (c) CH_2_Cl_2_/2-propanol, Et_3_N, rt, 16 h, yield = 78%; (d) HCl_(aq)_ (6 M), reflux, 16 h, yield = quanti. for **6** and **10**; (e) divinyl sulfone, Na_2_CO_3_, H_2_O, rt, yield = 96% for **7** and quantitative for **11**; (f) HCHO, diisopropyl phosphite, THF, reflux, 16 h, yield = 57%.

With respect to the BP targeting moiety, two bidentate thiol-containing BPs (BP-SH) ((2-((3-mercaptopropyl)thio)ethane-1,1-diyl)diphosphonic acid) (**6**) and (((3-mercaptopropyl)azanediyl) bis(methylene))diphosphonic acid (**10**) were selected as the targeting moieties. These compounds differ in both the connecting backbones (P–C–P and P–C–N–C–P, respectively) in presenting the two chelating phosphonate groups and also in the length between the terminal nucleophilic thiol group and the pivotal C or N atom that supports the side chain. These differences introduce structural diversity to explore the influence of these factors on the targeting efficiency of the PEI-BP-CD ternary conjugates (Scheme 1). To access the BP-SH **6** and **10**, the corresponding thioacetyl tetraisopropyl phosphonate derivatives BPs-SAc, **5** and **9**, were first prepared following already known strategies: BP-SAc **5** starting from tetraisopropyl vinylidene diphosphonate **2** and BP-SAc **9** starting from diisopropyl phosphite. After acid hydrolytic cleavage of the ester groups, the BPs-SH **6** and **10** were quantitatively isolated and, in concordance with the VS-based conjugation strategy outlined above, these compounds were straightforwardly transformed into their VS derivatives (BPs-VS) **7** and **11** by the thiol-Michael addition click reaction^23^ with DVS, as the bis-VS of choice.

**Scheme 2:**
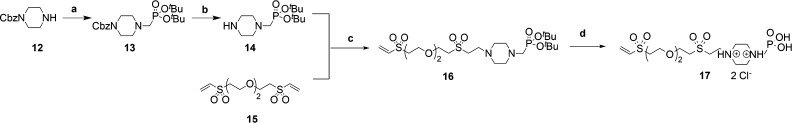
Synthesis of MP-VS Derivate 17 Reagents and conditions: (a) paraformaldehyde, di-*tert*-butyl phosphite, THF, reflux, 16 h, yield = 77%; (b) Pd/C, MeOH, rt, 16 h, yield = 96%; (c) CH_2_Cl_2_/2-propanol, Et_3_N, rt, 24 h, yield = 59%. (d) HCl_(aq)_ (2 M in Et_2_O), MeOH, rt, 30 min, yield = 98%.

In addition, an amino VS monophosphonate (MP-VS), compound **17**, was also prepared to be used as a control compound in the biological assays (Scheme 2). It is known that MPs are ineffective as inhibitors of bone resorption.^34^ The synthesis of **17** was performed using di-*tert*-butyl(piperazin-1-ylmethyl)phosphonate (**14**), an *N*-piperazine derivative prepared by a two-step procedure starting from 1-(benzyloxycarbonyl)piperazine (**12**): reaction with HCHO and di-*tert*-butyl phosphite, followed by quantitative deprotection of the N-protecting group. In this case, the VS functionalization was carried out using DVS-EEE instead of DVS leading to the desired MP-VS **17** after acid hydrolysis of the phosphonate ester groups.

**Scheme 3:**
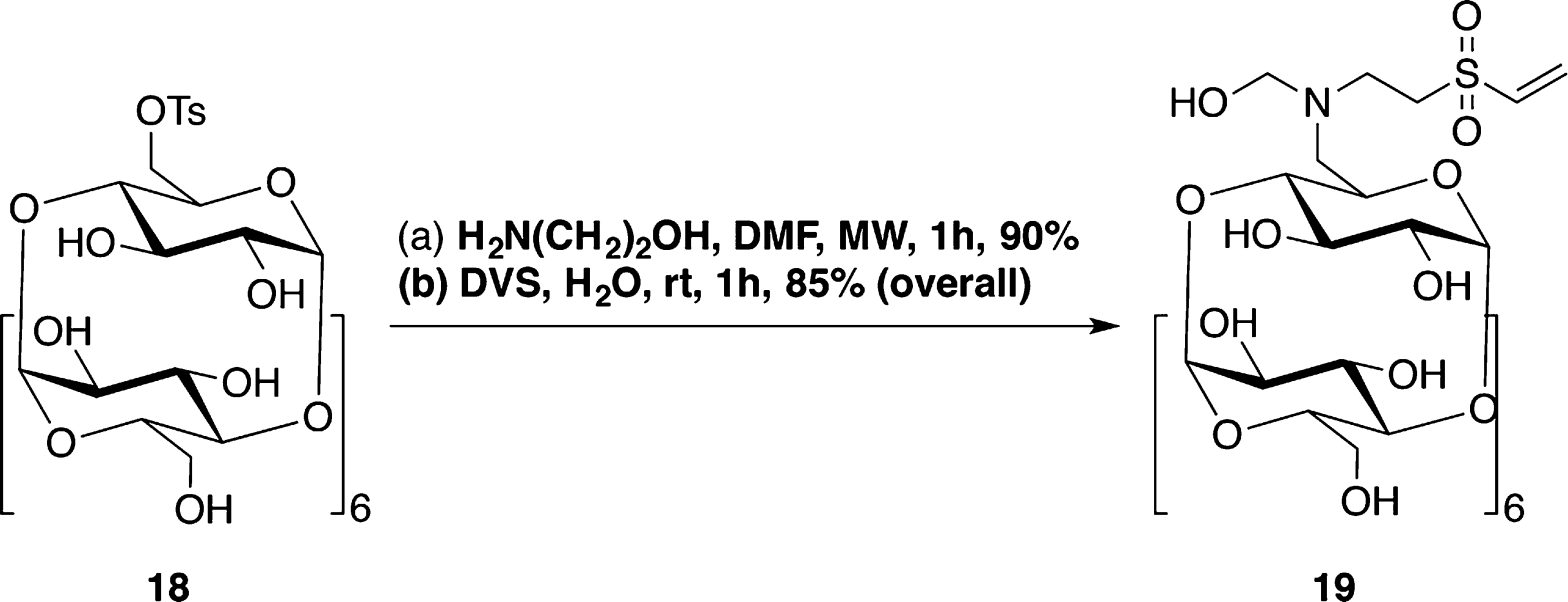
Synthesis of βCD-VS (**19**) Reagents and conditions: (a) H_2_N(CH_2_)_2_OH, DMF, MW, 1 h, yield = 90%; (b) DVS, H_2_O, rt, 1 h, yield = 85% for the two steps.^1^

Regarding the drug carrier moiety, the modularity of the assembly methodology enables a flexible selection of the CD drug vehicle that best fits the anticancer agent to be delivered, as commented above. In the present study, DOX was selected to be tested in the biological assays. DOX is a well-known anthracycline with a broad antitumor activity that is usually administered in a large variety of tumors.^35^ However, its potential use is limited due to its cumulative and dose-dependent cardiotoxicity.^36^ Therefore, efforts have been made to confer DOX specificity toward tumor cells and to decrease unwanted side effects. Notably, DOX is a commonly used chemodrug to treat primary malignant bone tumors such as osteosarcoma^37^ and metastatic bone tumors.^38,39^ For the delivery of DOX mediated by BPs, diverse binary DOX-BP conjugated prodrugs^40,41^ and also ternary polymer-BP-DOX delivery systems^20−22^ have been previously designed and tested. However, DOX is also well-known because it interacts with the hydrophobic cavities of βCD through supramolecular hydrophobic interactions.^13,42^ This property was used to confer specificity to the antitumoral through the covalent linking of the βCD moiety to diverse director molecules, including BPs.^43,44^ For this reason, the tandem DOX-βCD was considered a proof of concept for the proposed PEI-BP-CD ternary supramolecular systems. For the implementation of the VS-based assembly methodology, (6-deoxy-6-(2-hydroxyethyl)(vinylsulfonyl)-methyl)amino-β-CD (βCD-VS, **19**) was prepared by following a two-step procedure already reported by us:^1^ the microwave-assisted reaction of 6-*O*-monotosyl-6-β-CD (**18**) with ethanolamine to yield mono-6-(2-hydroxyethyl)amino-β-CD and concomitant aza-Michael click reaction of this compound with DVS (Scheme 3). In this way, the required VS-CD derivative was easily accessible.

**Scheme 4:**
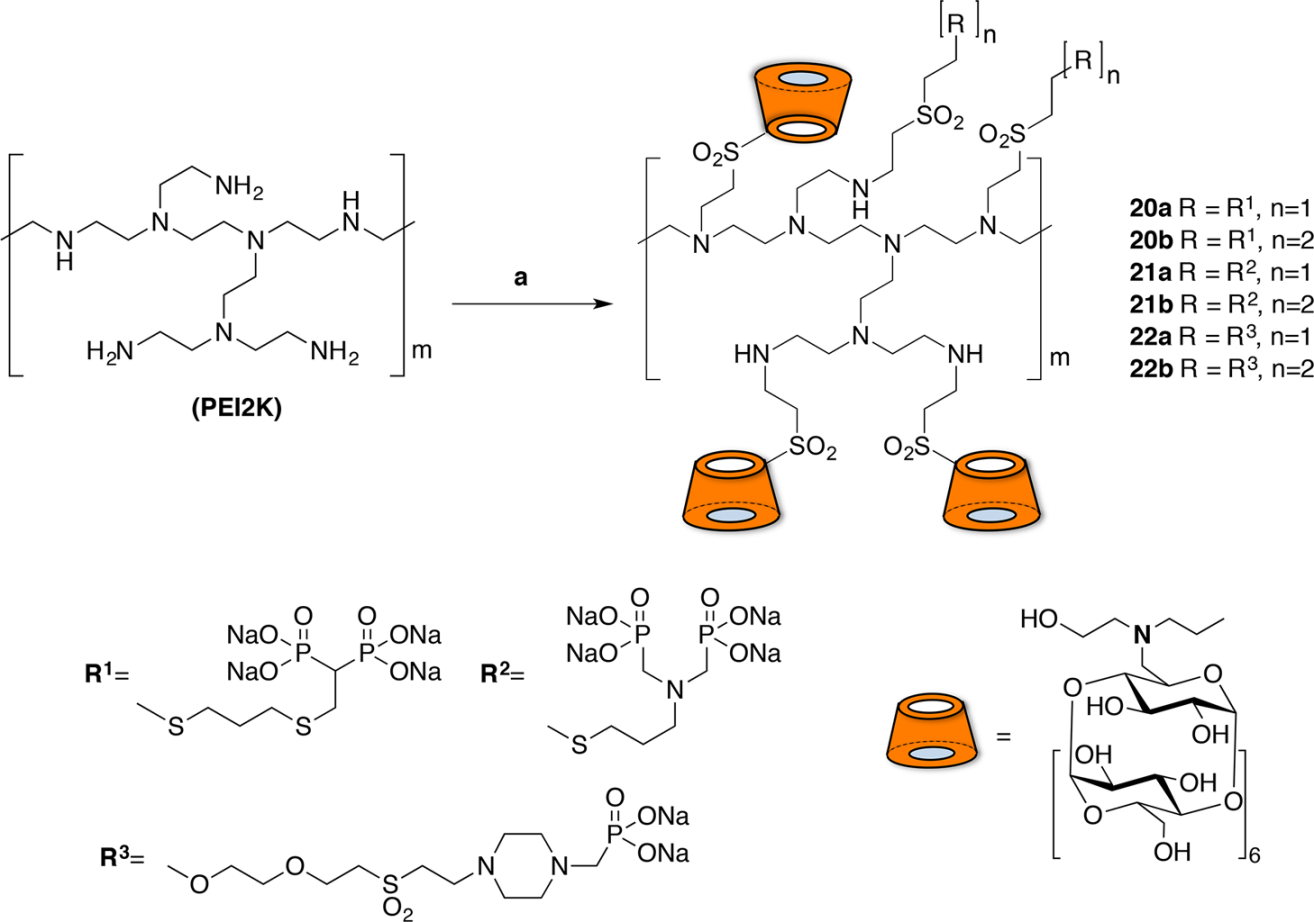
Synthesis of PEI-BP-CD (**20–21a,b**) and PEI-MP-CD (**22a,b**) Ternary Systems Reagents and conditions: (a) 19, H_2_O/DMSO, rt, 72 h. (b) 7, 11 or 17, rt, 72 h.

Once the different VS derivatives were available, we proceeded to their modular conjugation to the reactive primary amino groups of the PEI backbone by the straightforward aza-Michael addition click reaction by a simple mixture and stirring of the reagents in H_2_O (Scheme 4).

The reactions were performed at room temperature for a reaction time that was extended (48 h) to ensure completion of the covalent grafting. A set of six ternary conjugates **20–22a,b** were obtained from the BPs **7** or **11**, and the MP **17**. The conjugates were prepared using 1:1 and 1:2 PEI-BP or PEI-MP ratios to obtain systems differing in the density of the targeting ligand. The loading capacity was fixed using a 1:4 PEI-CD ratio that enabled the incorporation of four CD units per conjugate. The particle size was determined for the ternary system, and sizes ranging from 450 to 550 nm were obtained (Table S2). As mentioned above, the MP conjugates PEI-MP-CD (**22a**,**b**) were prepared to be used as control compounds.

Finally, the loading of DOX was carried out in the PEI-BP-CD and PEI-MP-CD conjugates by incubating them in the presence of DOX using a 1:0.9 βCD-DOX molar ratio leading to the formation and isolation of the corresponding DOX ⊂ PEI-BP-CD (**DOX ⊂ 20-21a,b**) and DOX ⊂ PEI-MP-CD (**DOX ⊂ 22a,b**) inclusion complexes, ready for their biological evaluation.

### *In Vitro* Cytotoxicity of PEI-BP-CD Ternary Conjugates

Nitrogen-containing BPs have been described as antiresorptive compounds, mainly promoting osteoclast apoptosis by blocking the mevalonate pathway and preventing the prenylation of GTP-binding proteins, such as Ras.^45^ In contrast, non-nitrogenous BPs, such as clodronate, are intracellularly metabolized by osteoclasts to nonhydrolyzable ATP analogues that can also induce osteoclast apoptosis.^3^ With this background, the effects of the new PEI-BP-CD and PEI-MP-CD ternary conjugates on cell viability were assayed in three cell lines: HeLa, MC373-E1, and MG-63 cells. HeLa cells were selected as a non-bone-related negative control. Osteoblasts, MC3T3-E1 cells, and the tumor bone-related cell line (MG-63) were used as bone-derived cells. The MC3T3-E1 cells are an accepted model to study osteoblast functionality in cell cultures.^46^ In the human osteosarcoma cells MG-63, active uptake of BP derivatives as zoledronic acid has been reported.^47^

**Figure 3:**
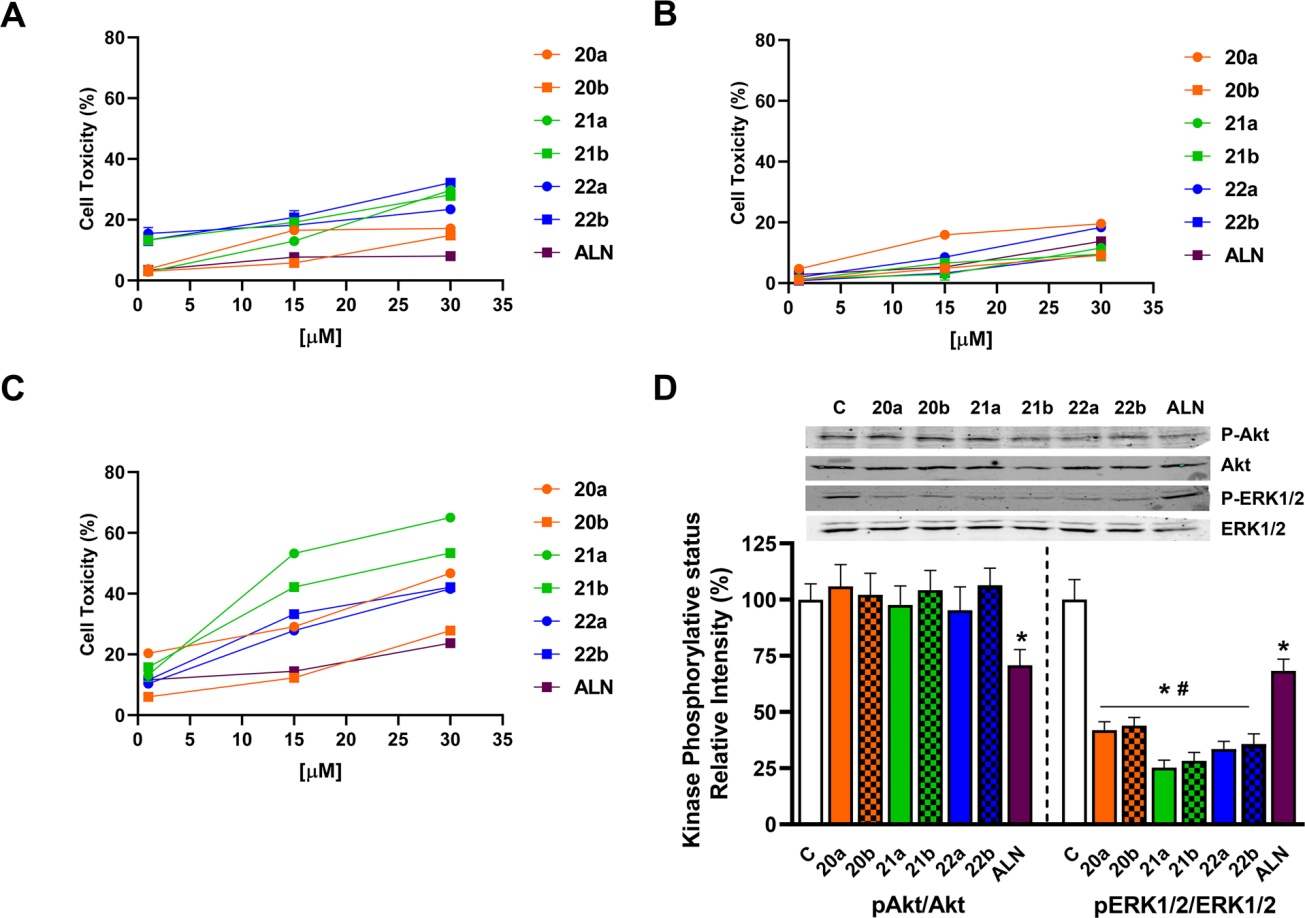
Cytotoxicity of PEI-BP-CD (**20–22a,b**) and PEI-MP-CD (**22a,b**) ternary conjugates in different cell lines. HeLa cells (A), MC3T3 osteoblasts (B), and MG-63 sarcomas (C) were incubated with 1–30 μM ALN or PEI-BP derivatives for 48 h, and the cell cytotoxicity (expressed as the percentage of the cell viability of the untreated cells minus the cell viability of treated cells) was determined by an MTT assay. Data are shown as mean ± SEM (*n* = 5). (D) MG-63 cells were incubated in the absence or presence of 15 μM PEI-BP or ALN for 30 min. The phosphorylative status of Akt and ERK1/2 was measured by Western blot. Data are shown as mean ± SEM (*n* = 4). *p < 0.05 *vs* nontreated (C) cells and #*p* < 0.05 *vs* ALN-treated cells.

First, their cytotoxicity was assayed and compared to ALN-mediated toxicity. The data in Figure 3 indicate that after 48 h of incubation, all ternary conjugates assayed showed limited cytotoxicity in HeLa cells and that the toxicity was even lower in MC3T3 osteoblasts. In contrast, significant cytotoxicity was detected for the PEI-BP-CD systems (**20–21a,b**) in the sarcoma cell line MG-63, where ALN has been reported as cytotoxic.^48^ In our cases, **21a,b** were significantly more cytotoxic compared to ALN. The combined high activity of **21a,b** on MG-63 cells and low toxicity in MC3T3 cells suggest specificity toward bone cancer cells.

It is known that BP conjugation to high-molecular-weight cationic polymers, poly-l-lysine and 25 kDa PEI, does not increase their natural affinity to HA. Moreover, under some conditions, the conjugation with 25 kDa PEI decreases the affinity of the polymers toward HA.^49^ These observations probably indicate that a balance between the positive charges provided by the PEI moiety and the negative charges of BP is needed to provide enhanced targeting toward bone. The increased cytotoxicity of the low-molecular-weight PEI-based ternary conjugates in bone cells presented in this article supports this idea, since in this case, BP conjugation does increase the affinity to HA.

Since BPs exert their cytotoxic effects blocking signaling pathways that promote cell proliferation, the effects of PEI-BP-CD ternary conjugates on key proteins were assayed. Akt and ERK1/2 kinases were selected, and their phosphorylation status was measured by Western blot. The activation of both kinases increases cell proliferation in cancer cells, since they have the ability to promote cell growth and to decrease apoptosis in tumor cells and osteoclasts.^50^ In these cells, ALN can block these signaling pathways.^51^ Furthermore, specific inhibition of the ERK1/2 signaling by 100 μM **ALN** in MG-63 can prevent differentiation and proliferation,^52^ blocking the PI3K–Akt–NFκB pathway.^53^

Our results (Figure 3D) show that PEI-BP-CD systems were able to significantly block ERK1/2 activation in the MG-63 cells, and the respective inhibition was significantly higher than the one detected after incubation with 15 μM ALN. Furthermore, **21a,b** showed a greater inhibitory effect. This is a remarkable effect when considering the inhibition of the Ras pathway described for nitrogen-containing BPs.^51^ The low molecular weight of the PEI used for the construction of the ternary conjugates is probably responsible for eliciting this response. In contrast, when the Ser473 phosphorylation of Akt, a characteristic target of the nitrogen-containing BPs, was tested in MG-63 cells, a moderate inhibition on the activation of this kinase was obtained by incubation with ALN, while the PEI-BP-CD systems did not affect its phosphorylative status. Taken together, the results indicate that PEI-BP-CD systems can specifically target sarcoma cells by decreasing cell viability. This effect could be ascribed to significant inhibition of the MAPK signaling pathway, as manifested by the inhibition of ERK1/2 phosphorylation. These effects are significantly higher than those obtained with ALN.

### Cell Uptake of DOX ⊂ PEI-BP-CD Complexes

Since conjugation of therapeutics or imaging agents to a BP moiety has been exploited to confer tissue specificity,^3,18^ we have occluded DOX into the CD moiety of the PEI-BP-CD systems to potentiate their therapeutic effects and to provide a targeting motive for the DOX.

**Figure 4:**
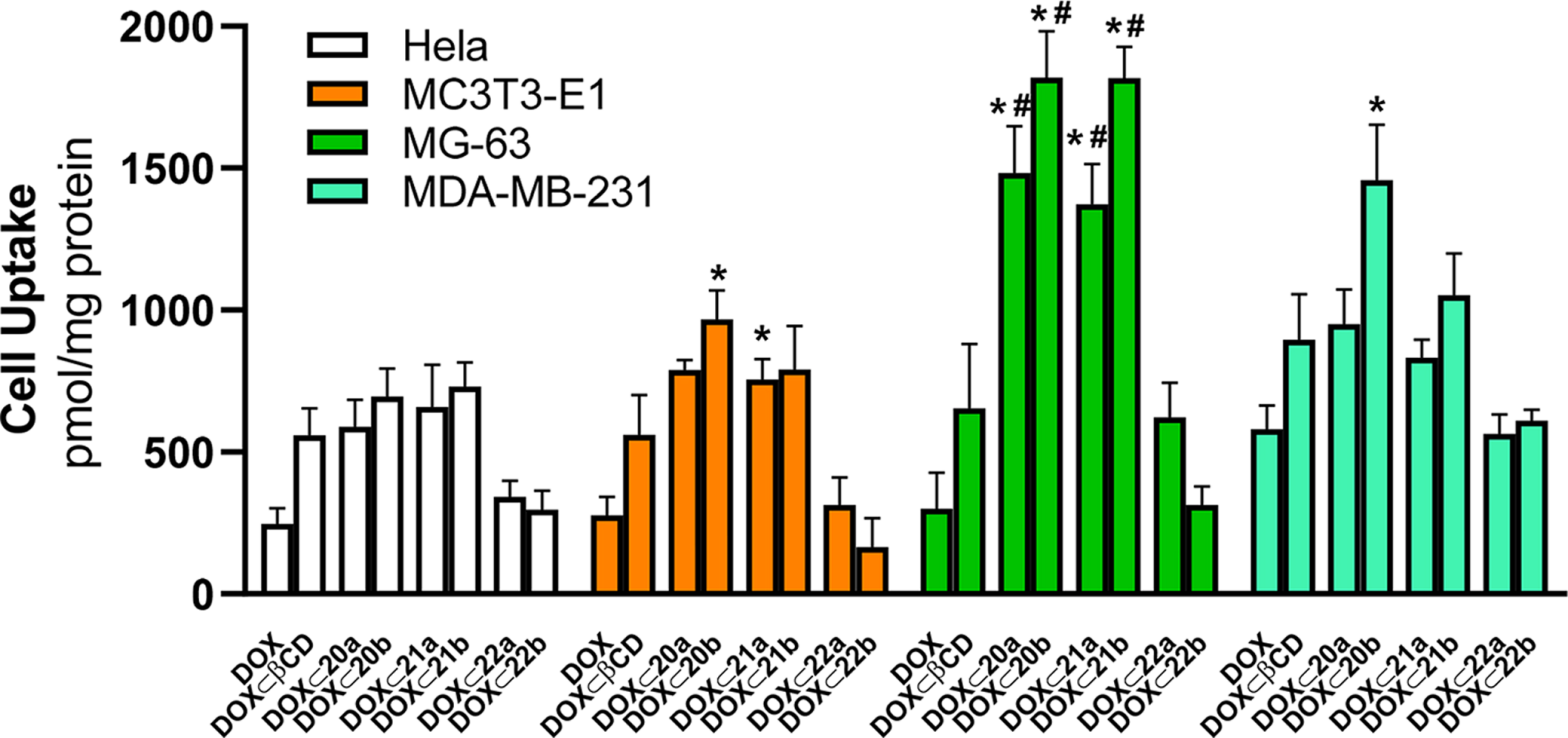
Cell uptake of **PEI-BP-CD** and **PEI-MP-CD** systems. HeLa, MC373-E1, MG-63, and MDA-MB-231 cells were incubated for 2 h in the presence of 1 μM DOX or an equivalent concentration of DOX occluded onto βCD and **PEI-BP-CD** (**20–21a,b**) and **PEI-MP-CD** (**22a,b**) systems. Results are expressed as pmol DOX/mg protein. The data are shown as mean ± SEM (*n* = 6). *p < 0.05 *vs* DOX-treated cells; #*p* < 0.05 *vs***DOX ⊂ βCD**-treated cells.

The cellular uptake of the DOX-loaded systems, (**DOX ⊂ 20-21a,b**) and **DOX⊂22a,b**, was evaluated by fluorimetry, exploiting the intrinsic DOX fluorescence and expressed as pmol **DOX**/mg protein (Figure 4). In addition to the HeLa, MC373-E1, and MG-63 cells, uptake was also assayed in MDA-MB-231 cells, a human breast cancer cell line. This cell line is a known model for bone-related cancer metastases, and the uptake of the BP zolendronic acid and ibandronate has been reported.^54,55^ Data corroborate the expectations of the rational design. First, an improved DOX uptake in all cell lines was observed with respect to free DOX when the antitumoral was occluded in native βCD. Second, there was a further uptake increase when DOX was occluded in ternary conjugates, particularly in HA-enriched cells, MG-63 and MDA-MB-231. This effect was less obvious in HeLa cells and the osteoblast MC3T3-E1 cell line. Third, the BP-based ternary conjugates (**20-21a,b**) promoted a better cell uptake compared to the MP-based systems (**22a,b**), a result in agreement with the one obtained on cell viability (Figure 3).

The highest uptake has been observed for DOX occluded into the BP-based ternary conjugates, **20–21a,b**, when assayed in MG-63 cells, suggesting specificity toward bone-related cancer cells and the capability to mediate a directed transport in those cells. Previous reports indicate that the uptake of functionalized BPs (e.g., pamidronate labeled with near-infrared fluorophores^56^) is dependent on their physicochemical properties such as net charge, hydrophobicity, and polarity, allowing their use as a specific imaging agent in the detection of bone-related tumors.^18^ On this basis, the preferential sarcoma cell uptake of the BP-based ternary conjugates (**20–21,ab**) could be tentatively explained by their physicochemical properties associated with the incorporation of positive charges provided by the protonated amino groups of the PEI moiety and also by the modulation of the hydrophobicity due to the presence of βCD. The targeted delivery of these BP-based ternary conjugates to HA-bearing bone cells could enable the accumulation of a high dose of the therapeutic. This is a promising result since the increased DOX uptake in sarcoma cells when delivered by PEI-BP-CD systems could allow the decrease in the DOX dose without hampering efficacy and therefore decreasing its toxicity in patients with bone tumors.

Finally, the BP-based ternary conjugates, **20–21a,b**, also showed facilitated transport of DOX into the MDA-MB-231 cells. These cells correspond to triple-negative breast cancer, which is characterized by the lack of specific receptors that allow directed drug delivery. These cells are also able to easily originate bone metastases. The capacity of some of the BP-based ternary conjugates (particularly, **20–21b**) to increase the DOX uptake by these cells could point out an alternative approach to providing a specific treatment for this type of tumor. BP-functionalized anticancer compounds such as phenylacetate-BP have been tested in an animal model of breast cancer having proapoptotic and antiangiogenic effects.^57^ Moreover, the use of fluorescent BPs targeting HA can increase the specificity and sensitivity of imaging techniques such as mammography for the detection of breast cancer.

### Internalization Routes of DOX ⊂ PEI-BP-CD Complexes

**Figure 5:**
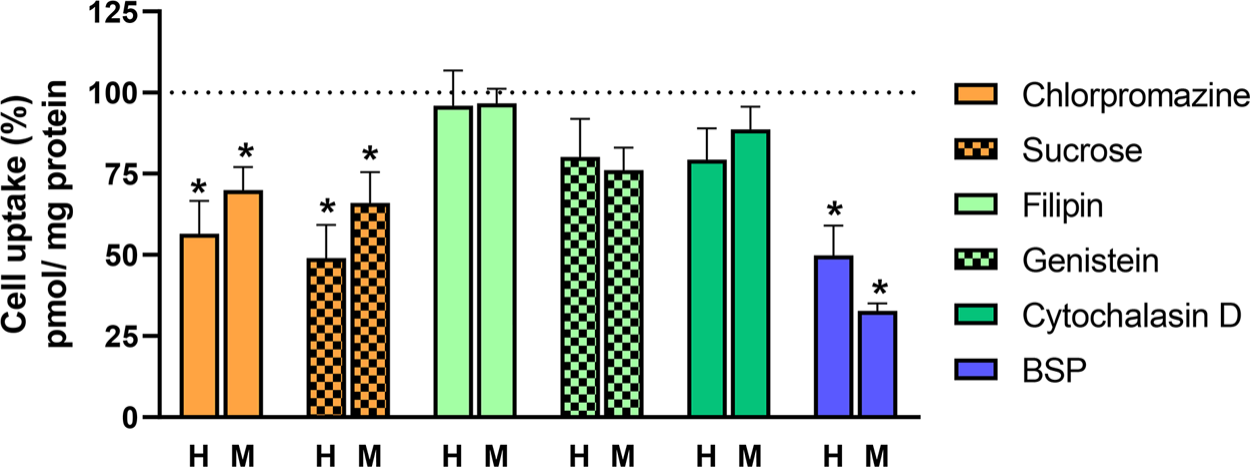
Effects of inhibitors of internalization routes on **DOX ⊂ 21b** uptake. HeLa (H) and MG-63 sarcoma (M) cells were pretreated with chlorpromazine (50 μM), sucrose (0.45 M), filipin (5 μg/mL), genistein (400 μM), cytochalasin D (2 μM), or BSP (0.25 mM) for 30 min before incubation with **DOX ⊂ 21b** (1 μM). DOX uptake was determined 2 h later. Results are expressed as relative uptake values normalized for a 100% value in the absence of inhibitors for each cell line as mean ± SEM (*n* = 6). *p < 0.05 *vs***DOX ⊂ 21b**-treated cells.

The observation that DOX occluded into PEI-BP-CD systems has a higher uptake in sarcoma cells compared to non-bone-related cells could be ascribed to structural modifications affecting the tropism of BP toward HA-containing cells. However, the use of alternative pathways by these compounds that would allow a better uptake in bone or cancer cells cannot be precluded. Therefore, the internalization of the PEI-BP-CD systems was next investigated using different inhibitors of the internalization routes. The assays were limited to the uptake of **DOX ⊂ 21b** in HeLa and MG-63 cells. After preincubation with the inhibitors for 30 min, the uptake of DOX ⊂ 21b was determined. Results are shown measured as pmol DOX/mg protein (Figure S70), and in Figure 5 normalized to 100% uptake. At this point, it is important to recall that nanoparticles use several different endocytic pathways to enter mammalian cells. Usually, they use clathrin-dependent and caveolae-mediated endocytosis pathways.^58^ The inhibitors of the clathrin-dependent route are chlorpromazine^59^ and sucrose,^60^ while filipin and genistein^59^ inhibit the caveolae-mediated endocytosis route.

Data indicate that the cell uptake of DOX ⊂ 21b in both HeLa and MG-63 cells could be partly mediated by the clathrin-dependent route, while the uptake was not changed after the preincubation with genistein or fili-pin, which inhibited caveolae endocytosis at the plasma membrane (Figure 3). Additionally, we have assayed in both cell lines the effects of cytochalasin D, an inhibitor of macropinocytosis and phagocytosis on the occluded DOX uptake. It has been observed that cytochalasin B did not affect the uptake of this compound, confirming that a preferred uptake route is clathrin-dependent in both cell lines.

Finally, we have studied whether their uptake takes place through organic anion-transporting peptides (OATPs), given that the PEI-BP-CD ternary conjugates have anionic groups.^58^ Members of the OATP family are capable of transporting a wide variety of structurally divergent drugs and bromosulfophthalein (BSP) is a competitive inhibitor of these transporters.^59^ With this rationale, the effect of BSP on the uptake of DOX mediated by **21b** in HeLa and sarcoma cells was assayed. Our results showed that preincubation with BSP significantly decreased (70%) the uptake of **DOX ⊂ 21b** in sarcoma cells, while BSP only produced a 50% inhibition of the uptake. This finding suggests that the cellular uptake of PEI-BP-CD systems in sarcoma cells is mediated preferentially by OATP and, to a lesser extent, by clathrin-dependent endocytosis.

### Cytotoxicity of DOX ⊂ PEI-BP-CD Complexes

**Figure 6:**
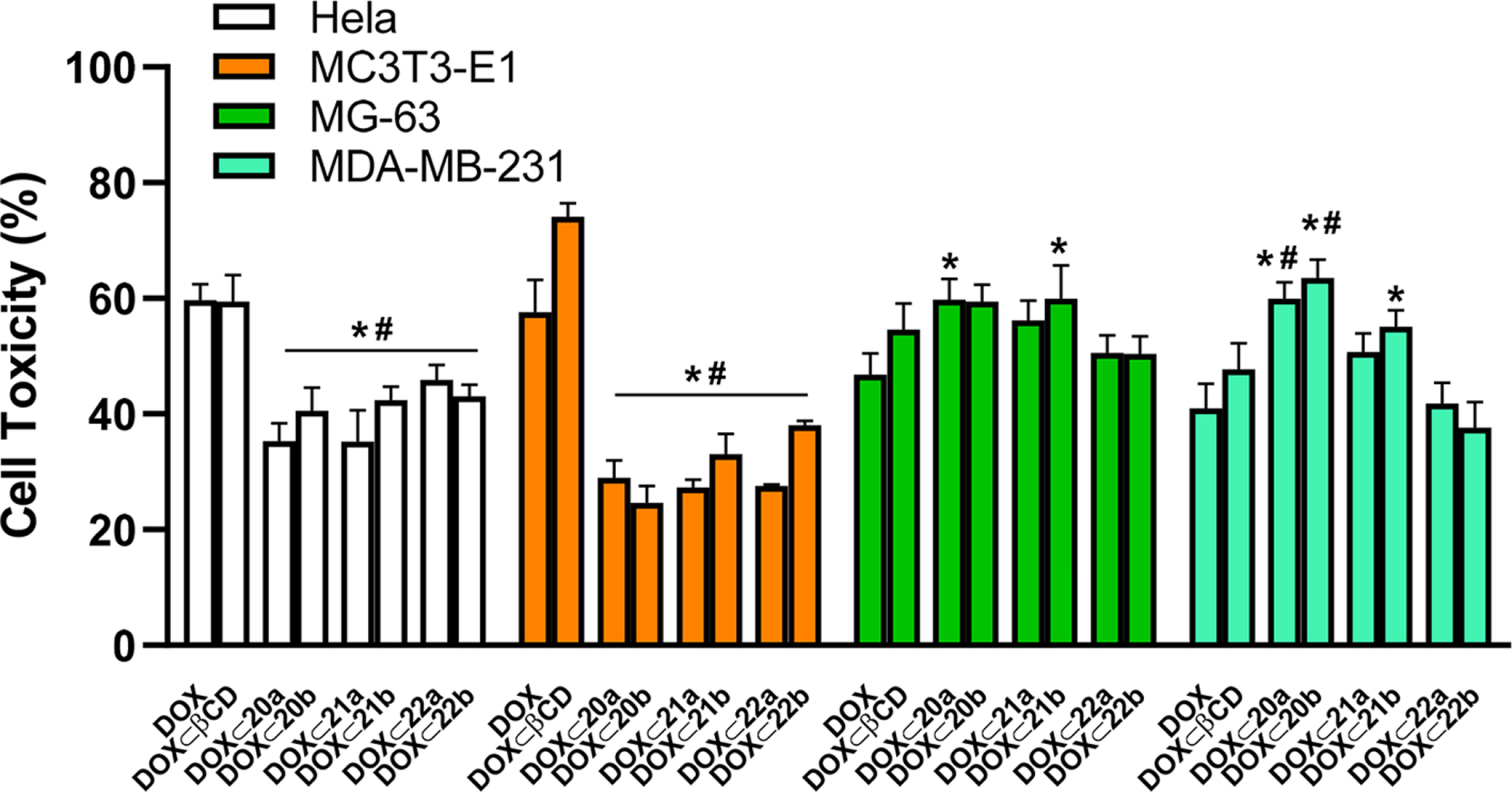
Cytotoxicity of DOX and DOX inclusion complexes in different cell lines. HeLa, MC373-E1, MG-63, and MDA-MB-231 cells were incubated for 48 h in the presence of 1 μM DOX or an equivalent concentration of DOX occluded onto βCD, PEI-BP-CD, and PEI-MP-CD conjugates. The cell cytotoxicity (expressed as the percentage of the cell viability of the untreated cells minus the cell viability of treated cells) was determined by an MTT assay. The data are shown as mean ± SEM (*n* = 10). *p < 0.05 *vs* DOX-treated cells; #*p* < 0.05 *vs* DOX ⊂ βCD-treated cells.

Once the preferential uptake of the DOX occluded into PEI-BP-CD ternary conjugates by sarcoma and breast cancer cells was elucidated, the effects of the occluded, delivered, and released DOX on the cell viability were assayed. The experiments were performed in the whole set of cells (HeLa, MG-63, MC3T3 osteoblasts, and MDA-MB-23) using **DOX**, either free or occluded into βCD as controls, and incubation periods of 48 h. Then, the cell viability was determined by MTT assay, and the cytotoxicity was deduced (Figure 6).

In general, cell cytotoxicity results are in agreement with the cell uptake data for the PEI-BP-CD ternary conjugates (Figure 4). DOX and DOX ⊂ βCD showed relevant cytotoxicity in all cell lines tested, as expected from the intrinsic toxicity of this antineoplastic. In contrast, in HeLa cells and MC3T3 osteoblasts, occlusion of DOX into PEI-BP-CD ternary conjugates (**20–21a,b**) produced a significant decrease in cytotoxicity compared to free DOX or DOX ⊂ βCD. However, in the bone-related cancer MG-63 and MDA-MB-231 cells, the occlusion of DOX produced an increase in cytotoxicity. These results are significant for the **20a** and **21b** derivatives in MG-63 sarcoma cells, while in MDA-MB-231 cells, **20a,b** derivatives produced the highest increase in cytotoxicity. Finally, and also in parallel with the uptake data, the MP-based derivatives **22a,b** failed to produce a significant targeted transport of DOX to the cells and the concomitant enhancement of specificity of the antitumoral.

### Subcellular Distribution of DOX ⊂ PEI-BP-CD

Next, the subcellular distribution of the delivered DOX was assayed. The experiment was performed by confocal microscopy on the MG-63 cell line and limited to the **DOX ⊂ 20a** complex considering its higher uptake in those cells, using DOX alone and **DOX ⊂ βCD** as controls.

**Figure 7:**
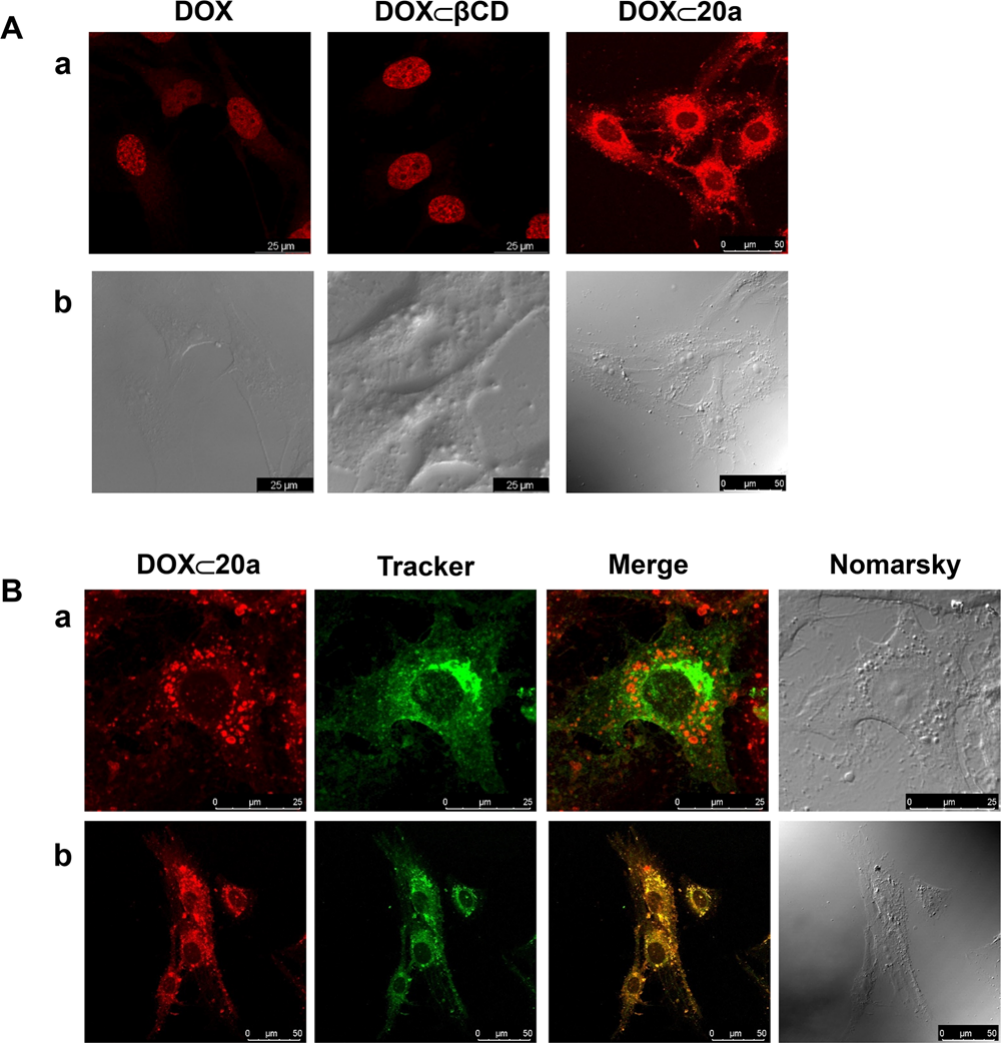
Subcellular distribution of **DOX ⊂ 20a**. (A) MG-63 sarcoma cells were incubated for 2 h with DOX, DOX ⊂ βCD, or **DOX ⊂ 20a**, and then, confocal images were obtained (fluorescent (a) and Nomarski (b) images are shown). (B) MG-63 cells were preincubated for 30 min with either Alexa 488-labeled cholera toxin as a marker of late endosomes (a) or green mitotracker as a mitochondrial marker (b), and then, cells were incubated for 1 h with **DOX ⊂ 23a**, and sequential confocal fluorescence images were obtained.

The results (Figure 7A) indicate a significant location of the DOX fluorescence after 2 h of incubation in the nuclei of the DOX- and DOX ⊂ βCD-treated cells. This observation is in accordance with the described nuclear tropism for DOX.^60^ Interestingly, an intense perinuclear punctured distribution pattern and a smaller allocation in the nucleus was detected for **DOX ⊂ 20a**. A similar pattern of distribution was confirmed for DOX occluded into **23b** and **24b** systems (Figure S71).

To determine more precisely the subcellular distribution of **DOX ⊂ 20a**, MG-63 cells were preincubated for 30 min with Alexa488-labeled cholera toxin, as a marker of caveolae-dependent endosomes in the endocytic route or mitotracker green, as a marker of mitochondria, prior to the 1 h incubation with **DOX ⊂ 20a**. Confocal images (Figure 7Ba) indicate a high rate of colocalization of **DOX ⊂ 20a** and the mitotracker, compatible with a mitochondrial location. In contrast, the fluorescence of **DOX ⊂ 20a** was not associated with endosomes (Figure 7Bb) since the fluorescence due to DOX did not colocalize with Alexa488-labeled cholera toxin.

**Figure 8:**
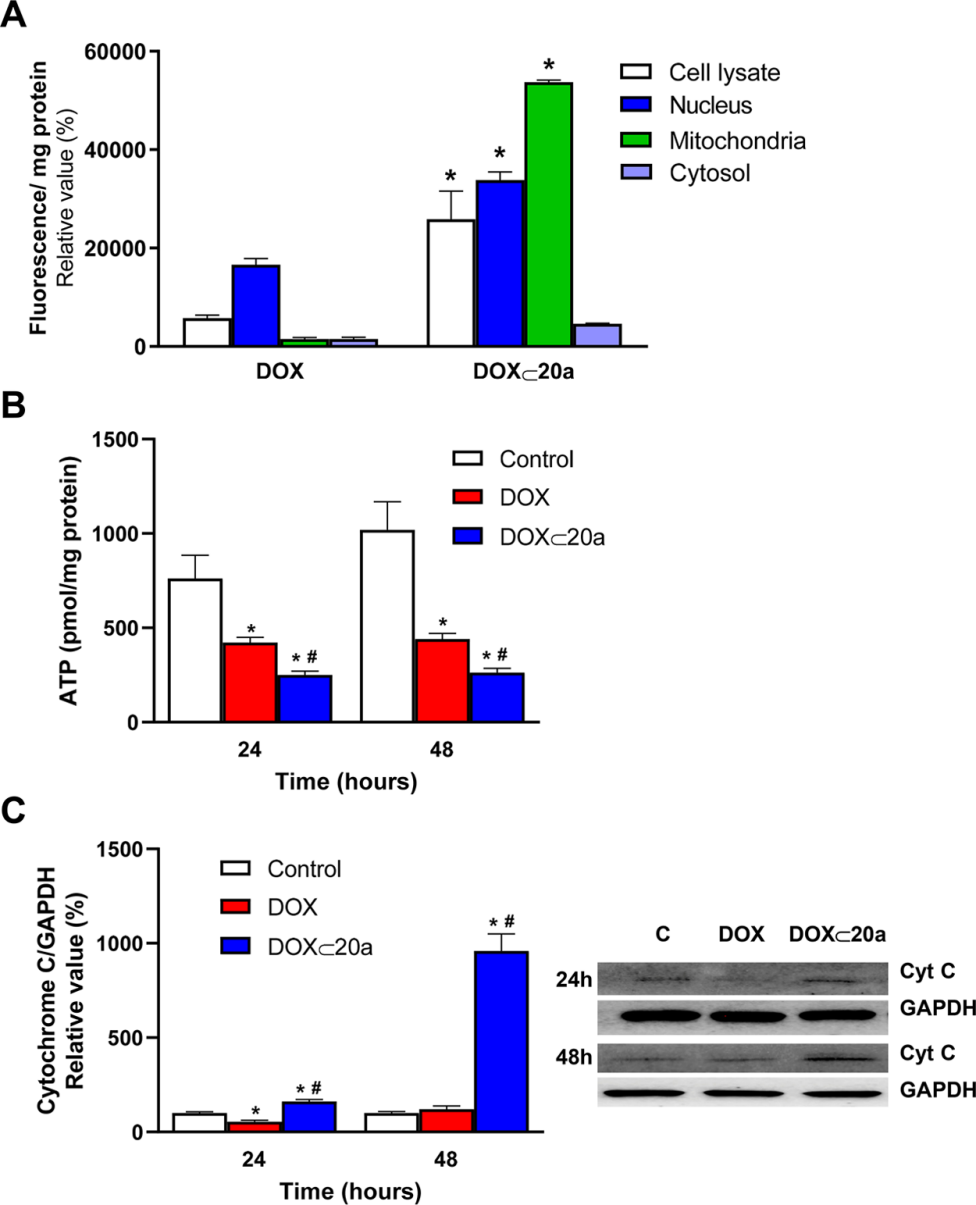
Mitochondrial targeting of the **DOX ⊂ 20a** inclusion complex. (A) MG-63 cells were incubated in the absence or presence of 1 μM DOX or equivalent concentration occluded in the **DOX ⊂ 20a** complex for 24 h. A subcellular fractionation of the MG-63 cells was carried out and fluorescence was measured in each fraction. Results are mean ± S.E.M. (*n* = 4). *p < 0.05 compared to DOX-treated cells. (B) MG-63 cells were treated under the same conditions as in A for 24 or 48 h, and the ATP concentration was determined in the mitochondrial fraction. (C) MG-63 cells were treated under the same conditions as in B, and the release of cytochrome *c* to the cytosol was measured by Western blot in the cytosolic fraction. Results are mean ± S.E.M. (*n* = 4). *p < 0.05 compared to untreated cells. #*p* < 0.05 compared to DOX-treated cells.

To further confirm the subcellular allocation of **DOX ⊂ 20a** on the MG-63 cells, these cells were incubated either with free **DOX** or **DOX ⊂ 20a** for 24 h and then a subcellular fractionation was carried out. For each fraction, specific markers of cytosol, mitochondria, and nucleus were assayed to verify the enrichment (Table S2). The DOX fluorescence was evaluated in the cell lysates as well as in nuclear, mitochondrial, and cytosolic fractions (Figure 8a). Data show a preferential location of DOX fluorescence in the nuclear fraction, in accordance with the results of confocal microscopy. In contrast, the incubation with **DOX ⊂ 20a** led to an enhanced uptake (measured by fluorescence in the lysate fraction), correlated with a main allocation in the mitochondrial and nuclear fractions. Therefore, from these results, it is possible to propose that **DOX ⊂ 20a** mediates not only a preferential uptake by the MG-63 cells but also a targeted mitochondrial location. Moreover, it cannot be discarded that upon partial dissociation of the inclusion complex in the cytosolic fraction, a fraction of free DOX would target the nucleus of the cells, as the confocal microscopy data and subcellular fractionation indicate.

Recently, targeting mitochondria of osteosarcoma cells has been proposed as an effective therapeutic strategy to treat drug-resistant tumor cells. DOX targeting to the mitochondria was achieved by chemical modification of the compound by including nitro groups and the resulting compound was termed nitrooxy-DOX.^61^ The toxicity of DOX targeting to the mitochondria relies not only on the topoisomerase inhibition but also on a decrease in mitochondrial respiration. In the mitochondria, nitrooxy-DOX decreases the flux through the Krebs cycle and the activity of complex I and, consequently, a diminished ATP synthesis. It also stimulates the release of cytochrome *c*. These changes are associated with nitrooxy-DOX-induced apoptosis. However, DOX compounds directed to the mitochondria can have deleterious effects on the heart, an organ based on aerobic mitochondria metabolism, since nitrooxy-DOX lacks specificity toward cancer cells.^62^ With this body of knowledge, we have evaluated two parameters associated with the proposed effects onto the mitochondria: the mitochondrial ATP synthesis and the cytochrome *c* release.

The cells were treated with the **DOX ⊂ 23a** inclusion complex and free DOX as the control. Although DOX decreases ATP levels, this decrease is significantly higher in the **DOX ⊂ 23a**-treated cells, supporting the idea of a mitochondrial targeting of the inclusion complex (Figure 8B). With respect to the cytochrome *c* release to the cytosol, the **DOX ⊂ 20a**-treated cells exhibit a strong signal by Western blot, while in the DOX-treated cells, the release was significantly lower and similar to the untreated cells (Figure 8C). Taken together, these results point to a mitochondrial targeting of the DOX ⊂ PEI-BP-CD complexes and support an alternative mechanism for the cytotoxicity of these compounds based on apoptosis.

### *In Vivo* Targeting of PEI-BP-CD Inclusion Complexes

**Figure 9:**
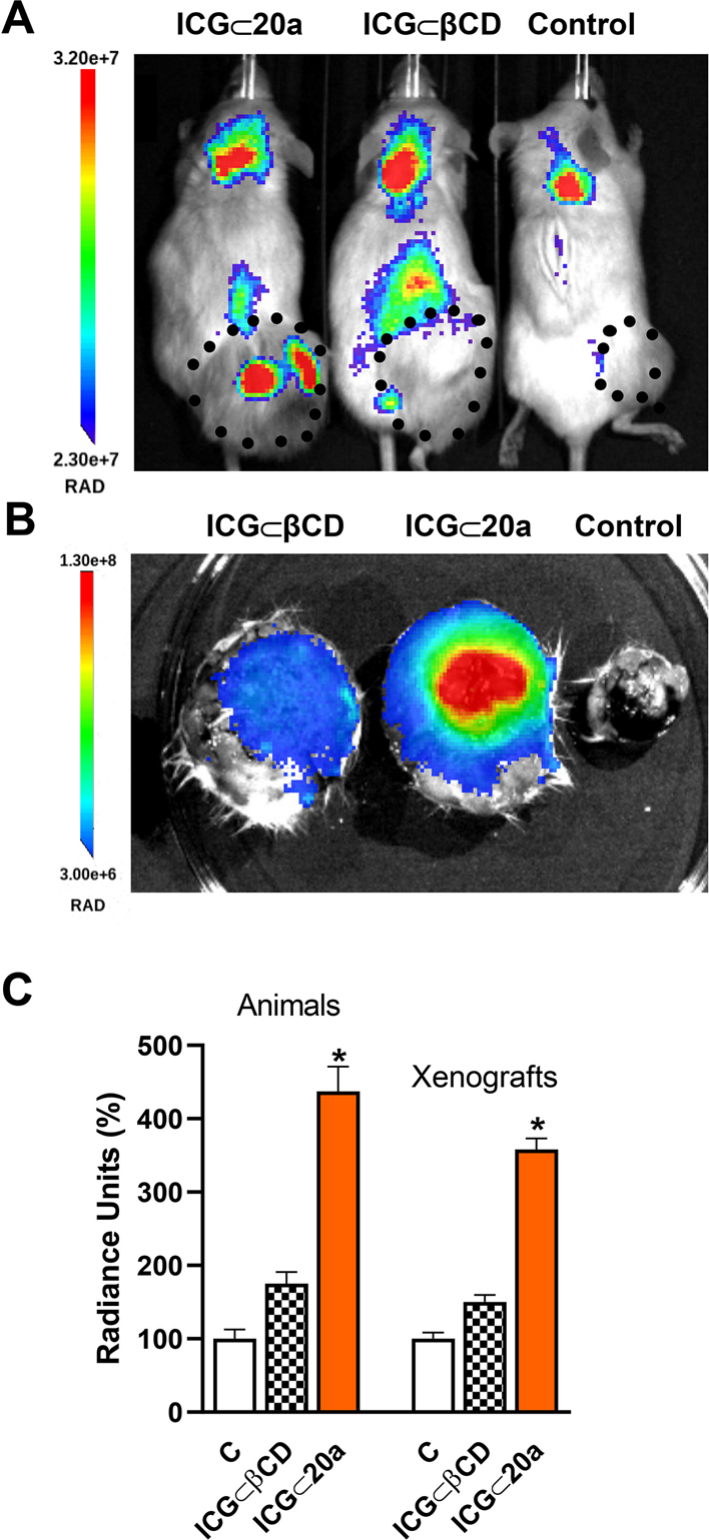
*In vivo* imaging of tumor MG-63 xenografts in mice. NSG mice bearing sarcoma (MG-63 cells) tumors were injected intravenously in the tail vein with IGC ⊂ βCD or **IGC ⊂ 20a** complexes and fluorescence was measured 30 min later. (A) ICG fluorescence images. The size of the xenografts is indicated by a dotted line. (B) Fluorescence imaging of dissected xenografts. (C) Average radiance of the xenografts *in vivo* and dissected xenografts. Data are shown as mean ± SEM (*n* = 4). *p < 0.05 *vs* C animals.

The capability of DOX ⊂ PEI-BP-CD inclusion complexes to target tumor cells was finally tested *in vivo* in xenograft animal models. The assays were performed in NSG mice bearing MG-63 xenografts using indocyanine green (ICG) as a DOX surrogate, considering not only its easy occlusion into βCD but particularly its spectral properties that facilitate detection by infrared emission.^63^ Animals were injected in the tail vein with the ICG ⊂ βCD and **ICG ⊂ 20a** complexes and visualized 30 min later (Figure 9A). A significantly higher fluorescence signal was detected in the tumor area (dotted line) of the **ICG ⊂ 20a**-treated animals compared to those injected with **ICG ⊂ βCD**. In both experiments, a significant liver signal was also detected. To further confirm the tumor targeting, the animals were sacrificed, the tumors were excised, and their associated fluorescence was evaluated, observing a similar pattern of distribution of fluorescence as in the animals (Figure 9B,C).

Finally, a similar *in vivo* experiment was carried out using breast cancer xenografts (MDA-MB-231 cells) considering the reported capability of these cells to metastasize into bone (Figure S72). In this case, the animals were injected with the **ICG ⊂ 21b** complex. The imaging results proved to be similar to those obtained with sarcoma cells with the ICG fluorescence specifically allocated in the xenografts.

Taken together, the *in vivo* results confirm the usefulness of the PEI-BP-CD ternary conjugates for the supramolecular targeting delivery to cancer tumors and metastases. The delivery capability of these compounds could be also enhanced by an enhanced permeability and retention effect due to its particle size (Table S2). Moreover, the supramolecular hosting capabilities can also be exploited in cancer imaging techniques.

## Conclusions

In summary, we present a modular and versatile design for multicomponent polymer-bisphosphonate-CD conjugates. These ternary systems are optimal supramolecular drug-delivery systems to bone cancer cells and metastases. They synergistically exploit BPs as targeting ligands and CDs as vehicles of antineoplastic drugs. The conjugates are open systems that can be tailored ad hoc to diverse therapeutic strategies because of the adaptability of the BP ligand module and the CD-drug tandem. The VS-based click chemistry used for the engineering of such nanoconstructions proves to be a convenient assembly technology to quickly generate delivery systems and to achieve structural variability. In this respect, PEI proves to be a suitable polymeric scaffold and bone-seeking nanoplatform. Moreover, the supramolecular hosting approach for the drug transport prevents the chemical modifications of the antitumoral and should facilitate the solubility and delivery of any hydrophobic drug by occlusion on the cavity of the CD appendages. The reported set of PEI-BP-CD ternary systems and their DOX occlusion complexes, prepared as a proof of concept, have been validated in selected bone-related cancer cell lines (MC3T3-E1, MG-63, and MDA-MB-231). The assays have also allowed elucidating *in vitro* valuable insights into their molecular biology: cytotoxicity due to an inhibition of the MAPK signaling pathway, internalization mainly mediated by cell membrane OATPs and to a lesser extent by the clathrin-dependent endocytosis route and, more importantly, mitochondrial targeting. This finding enables the PEI-BP-CD ternary conjugates to treat DOX-resistant cells, as an innovative approach. *In vivo* results confirm the usefulness of the PEI-BP-CD ternary conjugates for the supramolecular targeted delivery to cancer tumors and metastases and their potential in medicinal chemistry.

## Experimental Section

### Chemistry

Unless otherwise noted, commercially available reagents, solvents, and anhydrous solvents were used as purchased without further purification. Compounds **8**,^64^**12**,^65^**15**,^25^ and **β-CD-VS** (**19**)^1^ were prepared according to literature procedures. Thin-layer chromatography (TLC) was performed on Merck Silica gel 60 F_254_ aluminum sheets. The TLC plates were stained with potassium permanganate (1% w/v in water), sulfuric acid (50% v/v in water), or ninhydrin (0.3% w/v in ethanol) and observed under UV light when applicable. Flash column chromatography was performed with Silica gel 60 (VWR, 40–63 μm) with the solvent mixtures specified in the corresponding experiment. Nuclear magnetic resonance (NMR) spectra were recorded at room temperature on a Varian Direct Drive (400 MHz or 500 MHz), Bruker AVANCE III HD NanoBay (400 MHz), Bruker AVANCE Neo (400 MHz or 500 MHz), or Varian Direct Drive (600 MHz) spectrometers at a constant temperature of 298 K with tetramethylsilane as an internal reference. Chemical shifts δ are reported in parts per million (ppm). ^1^H NMR spectra were referenced to the residual partially nondeuterated solvent signal of CHCl_3_ (δ = 7.27 ppm), D_2_O (δ = 4.79 ppm), DMSO (δ = 2.50 ppm), or MeOD (δ = 3.31 ppm) or to the signal of the residual TMS (δ = 0.00 ppm). ^13^C NMR spectra were referenced to the deuterated solvent signal of CDCl_3_ (δ = 77.00 ppm), DMSO-*d*_6_ (δ = 39.51 ppm), or MeOD (δ = 49.00 ppm). ^31^P NMR spectra are referenced according to the unified chemical shift scale as recommended by the IUPAC.^66^ The collection of ^13^C and ^31^P NMR data was done with complete ^1^H decoupling. Coupling constants *J* are reported in Hz, and splitting patterns are described as m = multiplet, dd = double doublet, tt = triple triplet, td = triple doublet, p = quintet, t = triplet, d = doublet, and s = singlet. 2D NMR spectroscopy (HSQC) and diffusion ordered spectroscopy were used to analyze the different species present. IR spectra were recorded with a PerkinElmer Spectrum Two Fourier transform infrared attenuated total reflection spectrometer. Electrospray (ESI) HRMS spectra were recorded on a Waters Xevo G2-XS QTOF or a WATERS SYNAP G2. Unless specified otherwise, the purity of all final compounds was determined to be ≥95% by ^1^H NMR.

### Chemical Synthesis

#### Tetraisopropyl Ethene-1,1-diylbis(phosphonate) (**2**)

Compound **2** was prepared modifying the previously described procedure:^67^ A mixture of paraformaldehyde (4.36 g, 145 mmol, 5.00 equiv) and diethylamine (3.00 mL, 29.0 mmol, 1.00 equiv) in MeOH (50 mL) was refluxed until the complete dissolution of the reactant. The solution was cooled to room temperature and tetraisopropyl methylenediphosphonate (**1**, 10.0 g, 29.0 mmol, 1.00 equiv) was added. The resulting solution was refluxed for 6 days. The solution was cooled to room temperature, and the solvent was eliminated under reduced pressure. The resulting oil was dissolved in toluene (20 mL), and the solvent was evaporated again under reduced pressure. The resulting crude material was dissolved in dry toluene (250 mL), and a catalytic amount of *p*-toluenesulfonic acid was added. The reaction mixture was refluxed in a Soxhlet apparatus containing powdered calcium hydride (3 g) for 40 h. The mixture was cooled to room temperature, and the solvent was removed under reduced pressure. The crude material was purified by column chromatography (SiO_2_, hexane/acetone 1:1) to afford **2** (7.62 g, 74%) as colorless oil. ^1^H NMR (400 MHz, CDCl_3_): δ ^1^H NMR (400 MHz, CDCl_3_): δ 6.88 (dd, *J* = 37.9, 34.1 Hz, 2H), 4.70 (m, 4H), 1.32 (d, *J* = 6.2 Hz, 12H), 1.29 (d, *J* = 6.2 Hz, 12H). ^13^C NMR (101 MHz, CDCl_3_): δ 147.5, 134.9 (t, *J* = 168.7 Hz), 71.4 (m), 24.2 (m), 23.9 (m).

#### *S*-(3-Mercaptopropyl) Ethanethioate (**4**)

Under an inert atmosphere, acetic anhydride (1.89 mL, 20.0 mmol, 1.00 equiv) was added to a solution of 1,3-propanedithiol (**3**, 2.00 mL, 20.0 mmol, 1.00 equiv) in anhydrous CH_2_Cl_2_/pyridine (1:1, 10 mL). The solution was stirred for 16 h at room temperature. The solvent was removed under reduced pressure. The crude material was purified by column chromatography (SiO_2_, CH_2_Cl_2_/hexane 1:1) to afford **4** (1.40 g, 46%) as oil. ^1^H NMR (400 MHz, CDCl_3_): δ 2.96 (t, *J* = 7.1 Hz, 2H), 2.56 (m, 2H), 2.31 (s, 3H), 1.86 (p, *J* = 7.1 Hz, 2H), 1.38 (t, *J* = 8.1 Hz, 1H). ^13^C NMR (101 MHz, CDCl_3_): δ 195.7, 33.7, 30.7, 27.6, 23.5.

#### *S*-(3-((2,2-Bis(diisopropoxyphosphoryl)ethyl)thio)propyl) Ethanethioate (**5**)

A solution of **4** (346 mg, 2.30 mmol, 2.00 equiv) in anhydrous CH_2_Cl_2_ (5 mL), 2-propanol (0.50 mL), and Et_3_N (160 μL, 1.15 mmol, 1 equiv) was added to **2** (410 mg, 1.15 mmol, 1.00 equiv) under an Ar atmosphere. The resulting solution was stirred overnight (16 h) at room temperature under an Ar atmosphere. The solvent was eliminated under vacuum. The crude material was purified by column chromatography (SiO_2_, hexane/acetone 6:4) to afford **5** (454 mg, 78%) as oil. ^1^H NMR (400 MHz, CDCl_3_): δ 4.69–4.59 (m, 4H), 2.89–2.78 (m, 4H), 2.47 (t, *J* = 7.2 Hz, 2H), 2.29 (tt, *J* = 24.1, 5.8 Hz, 1H), 2.17 (s, 3H), 1.72 (p, *J* = 7.2 Hz, 2H), 1.20 (m, 24H). ^13^C NMR (101 MHz, CDCl_3_): δ 195.6, 71.6 (m), 40.9 (t, *J* = 132.9 Hz), 32.0, 30.7, 29.2, 28.2 (t, *J* = 4.9 Hz), 28.1, 24.3 (m), 24.0 (m). ^31^P NMR (162 MHz, CDCl_3_): δ 19.63. IR (neat): ν 2978, 1691, 1374, 1241, 1102, 977 cm^–1^. HR-MS (ESI^+^) *m*/*z*: 529.1595 [M + Na]^+^ (calcd for C_19_H_40_O_7_NaP_2_S_2_, 529.1588).

#### *S*-(3-(Bis((diisopropoxyphosphoryl)methyl)amino)propyl) Ethanethioate (**9**)

Diisopropyl phosphite (875 μL, 5.26 mmol, 2.00 equiv) was added to a mixture of **8** (651 mg, 2.63 mmol, 1.00 equiv) and paraformaldehyde (159 mg, 5.26 mmol, 2.00 equiv) in anhydrous tetrahydrofuran (THF) (5 mL), dropwise at room temperature, under an Ar atmosphere. The solution was refluxed overnight (16 h). The solvent was evaporated under reduced pressure. The crude material was purified by column chromatography (SiO_2_, CH_2_Cl_2_/MeOH 95:5) to afford **9** (0.72 g, 57%) as light-yellow syrup. ^1^H NMR (400 MHz, CDCl_3_): δ 4.64 (m, 4H), 3.05 (d, *J* = 8.2 Hz, 4H), 2.87–2.79 (m, 4H), 2.23 (s, 3H), 1.67 (p, *J* = 7.1 Hz, 2H), 1.24 (d, *J* = 6.2 Hz, 24H). ^13^C NMR (101 MHz, CDCl_3_): δ 195.5, 70.2 (t, *J* = 3.5 Hz), 54.9 (t, *J* = 7.3 Hz), 50.8 (dd, *J* = 155.9, 5.9 Hz), 30.4, 27.4, 26.4, 24.0 (m). ^31^P NMR (162 MHz, CDCl_3_): δ 22.73. IR (neat): ν 2979, 1690, 1375, 1210, 1140, 978 cm^–1^. HR-MS (ESI^+^) *m*/*z*: 512.1989 [M + Na]^+^ (calcd for C_19_H_41_NO_7_NaP_2_S, 512.1977).

### General Procedure for the Synthesis of Mercapto Diphosphonic Acids (**6** and **10)**

Under an inert atmosphere, a solution of HCl_(aq)_ (6 M, 10 mL) was added to the appropriate ethane thionate (**5** or **9**, 1 mmol, 1.00 equiv). The solution was refluxed overnight (16 h). The solvent was removed under reduced pressure to afford the corresponding thiols **6** and **10** as colorless syrup. They were characterized and used directly after evaporation of the solvent:

#### (2-((3-Mercaptopropyl)thio)ethane-1,1-diyl)diphosphonic acid **(6)** (74 mg, Quantitative) as Colorless Syrup

^1^H NMR (400 MHz, D_2_O): δ 2.96 (td, *J* = 16.1, 6.4 Hz, 2H), 2.65 (t, *J* = 7.2 Hz, 2H), 2.56 (t, *J* = 6.9 Hz, 2H), 2.50 (tt, *J* = 23.1, 6.4 Hz, 1H), 1.82 (p, *J* = 7.0 Hz, 2H). Spectral data agree with previously reported values.^40^

#### (((3-Mercaptopropyl)azanediyl)bis(methylene))diphosphonic acid (**10**) (318 mg, Quantitative) as Colorless Syrup

^1^H NMR (400 MHz, D_2_O): δ 3.60–3.49 (m, 6H), 2.56 (t, *J* = 6.8 Hz, 2H), 2.03 (p, *J* = 14.9, 7.0 Hz, 2H). ^13^C NMR (101 MHz, D_2_O): δ 55.8 (t, *J* = 3.9 Hz), 51.3 (dd, *J* = 137.4, 4.3 Hz), 27.2, 20.5. ^31^P NMR (162 MHz, D_2_O): δ 7.84.

### General Procedure for the Synthesis of Vinylsulfone Bisphosphonates **VS-BP** (**7** and **11**)

Divinyl sulfone (501 μL, 5 mmol, 5.0 equiv) and Na_2_CO_3_ (263 mg, 2.5 mmol, 2.5 equiv) were added to a solution of the corresponding mercapto diphosphonic acids (**6** or **10**, 1 mmol, 1.00 equiv) in H_2_O (10 mL) under an Ar atmosphere. The reaction mixture was stirred for 16 h at room temperature. The solution was diluted with H_2_O (20 mL) and washed with CH_2_Cl_2_ (5 × 20 mL). The aqueous phase was lyophilized to afford the corresponding vinyl sulfone derivatives **7** and **11** as white solids:

#### Sodium (2-((3-((2-(Vinylsulfonyl)ethyl)thio)propyl)thio) Ethane-1,1-diyl)bis(phosphonate) **(7)** (522 mg, 96%) as a White Solid

^1^H NMR (400 MHz, D_2_O): δ 6.90 (dd, *J* = 16.6, 10.1 Hz, 1H), 6.47 (d, *J* = 16.7 Hz, 1H), 6.40 (d, *J* = 10.0 Hz, 1H), 3.58–3.50 (m, 2H), 3.06–2.89 (m, 4H), 2.77–2.63 (m, 4H), 2.06 (tt, *J* = 20.7, 7.0 Hz, 1H), 1.91 (p, *J* = 7.1 Hz, 2H). ^13^C NMR (101 MHz, D_2_O): δ 134.3, 132.9, 53.3, 40.1 (t, *J* = 109.6 Hz), 30.9, 30.2, 28.6 (d, *J* = 3.8 Hz), 28.1, 23.1. ^31^P NMR (162 MHz, D_2_O): δ 17.94. IR (neat): ν 3098, 1650, 1360, 1083 cm^–1^. HR-MS (ESI^–^) *m*/*z*: 412.9706 [M – 4Na + 3H]^−^ (calcd for C_9_H_19_O_8_P_2_S_3_, 412.9717).

#### Sodium (((3-((2-(Vinylsufonyl)ethyl)thio)propyl)azanediyl)bis (methylene))bis (phosphonate) (**11)** (180 mg, Quantitative) as a White Solid

^1^H NMR (400 MHz, D_2_O): δ 6.91 (dd, *J* = 16.5, 10.1 Hz, 1H), 6.48 (d, *J* = 16.6 Hz, 1H), 6.40 (d, *J* = 10.0 Hz, 1H), 3.70–3.50 (m, 4H), 3.27 (d, *J* = 11.3 Hz, 4H), 2.97 (dd, *J* = 8.9, 6.4 Hz, 2H), 2.71 (t, *J* = 7.4 Hz, 2H), 2.24–2.01 (m, 2H). ^13^C NMR (126 MHz, D_2_O): δ 134.3, 132.8, 55.3, 54.1 (dd, *J* = 125.5, 4.1 Hz), 53.2, 27.9, 23.5, 23.2. ^31^P NMR (202 MHz, D_2_O): δ 6.33. IR (neat): ν 3100, 1739, 1366, 1088, 969 cm^–1^. HR-MS (ESI^+^) *m*/*z*: 398.0261 [M + H]^+^ (calcd for C_9_H_22_NO_8_S_2_P_2_, 398.0262).

#### Benzyl 4-((Di-*tert*-butoxyphosphoryl)methyl)piperazine-1-carboxylate (**13**)

Di-*tert*-butyl phosphite (503 μL, 2.49 mmol, 1.10 equiv) was added to a mixture of 1-(benzyloxycarbonyl)piperazine (**12**, 498 mg, 2.26 mmol, 1.00 equiv) and paraformaldehyde (75 mg, 2.49 mmol, 1.10 equiv) in anhydrous THF (5 mL), dropwise at room temperature, under an Ar atmosphere. The reaction mixture was refluxed overnight (16 h). The solvent was evaporated under reduced pressure. The crude material was purified by column chromatography (SiO_2_, EtOAc to EtOAc/MeOH 98:2) to afford **13** (745 mg, 77%) as oil. ^1^H NMR (400 MHz, CDCl_3_): δ 7.35–7.33 (m, 5H), 5.11 (s, 2H), 3.49 (t, *J* = 5.1 Hz, 4H), 2.64 (d, *J* = 11.7 Hz, 2H), 2.59 (t, *J* = 5.1 Hz, 4H), 1.49 (s, 18H). ^13^C NMR (101 MHz, CDCl_3_): δ 155.3, 136.9, 128.6, 128.1, 128.0, 82.3 (d, *J* = 9.0 Hz), 67.2, 57.2 (d, *J* = 167.9 Hz), 54.3 (d, *J* = 9.9 Hz), 44.0, 30.6 (d, *J* = 3.9 Hz). ^31^P NMR (162 MHz, CDCl_3_): 16.03. IR (neat): ν 2977, 1702, 1367, 1237, 979 cm^–1^. HR-MS (ESI^+^): *m*/*z* 449.2188 [M + Na]^+^ (calcd for C_21_H_35_N_2_O_5_NaP, 449.2181); 427.2366 [M + H]^+^ (calcd for C_21_H_36_N_2_O_5_P, 427.2362).

#### Di-*tert*-butyl (Piperazin-1-ylmethyl)phosphonate (**14**)

Pd/C (10%, 62 mg) was added to a solution of **13** (623 mg, 1. 46 mmol, 1.00 equiv) in dry MeOH (15 mL). The resulting suspension was stirred under a H_2_ atmosphere at room temperature overnight (16 h). Then, it was filtered through a pad of Celite and washed with MeOH (15 mL). The solvent was removed under reduced pressure to afford **14** (427 mg, quant.) as brown syrup. ^1^H NMR (400 MHz, MeOD): δ 3.03 (t, *J* = 5.1 Hz, 4H), 2.77–2.71 (m, 6H), 1.53 (s, 18H). ^13^C NMR (101 MHz, MeOD): δ 84.5 (d, *J* = 9.1 Hz), 57.9 (d, *J* = 168.8 Hz), 54.2 (d, *J* = 10.6 Hz), 45.7, 30.8 (d, *J* = 4.0 Hz). ^31^P NMR (162 MHz, MeOD): δ 15.80. IR (neat): ν 3389, 2978, 1632, 1370, 1038, 982 cm^–1^. HR-MS (ESI^+^) *m*/*z*: 293.1991 [M + H]^+^ (calcd for C_13_H_30_N_2_O_3_P, 293.1994).

#### Di-*tert*-butyl ((4-(2-((2-(2-(Vinylsulfonyl)ethoxy)ethyl) sulfonyl)ethyl)piperazin-1-yl)methyl)phosphonate (**16**)

A solution of 1,2-bis(2-ethenylsulfonylethoxy)ethane (**15**, 571 mg, 1.92 mmol, 5.00 equiv) in anhydrous CH_2_Cl_2_ (6 mL) and Et_3_N (53 μL, 0.38 mmol, 1.00 equiv) was added to a solution of **14** (112 mg, 0.38, 1.00 equiv) in 2-propanol (2 mL) under an Ar atmosphere. The resulting solution was stirred for 24 h at room temperature. The solvent was evaporated under vacuum. The crude material was purified by column chromatography (SiO_2_, CH_2_Cl_2_/MeOH 95:5) to afford **16** (276 mg, 59%) as syrup. ^1^H NMR (400 MHz, CDCl_3_): δ 6.71 (dd, *J* = 16.6, 9.9 Hz, 1H), 6.33 (d, *J* = 16.6 Hz, 1H), 6.06 (d, *J* = 9.9 Hz, 1H), 3.86–3.77 (m, 4H), 3.60–3.52 (m, 4H), 3.36 (t, *J* = 5.4 Hz, 2H), 3.20 (t, *J* = 5.8 Hz, 2H), 3.16 (t, *J* = 6.5 Hz, 2H), 2.75 (t, *J* = 6.5 Hz, 2H), 2.63–2.52 (m, 6H), 2.44 (sbr, 4H), 1.43 (s, 18H). ^13^C NMR (101 MHz, CDCl_3_): δ 137.8, 129.0, 82.1 (d, *J* = 9.0 Hz), 70.2, 70.1, 64.8, 64.5, 56.9 (d, *J* = 168.3 Hz), 54.8, 54.6, 54.4 (d, *J* = 10.5 Hz), 52.9, 52.1, 51.5, 30.5 (d, *J* = 3.9 Hz). ^31^P NMR (202 MHz, CDCl_3_): δ 16.11. IR (neat): ν 2977, 1368, 1314, 1118, 975 cm^–1^. HR-MS (ESI^+^) *m*/*z*: 591.2544 [M + H]^+^ (calcd for C_23_H_48_N_2_O_9_PS_2_, 591.2539); 613.2365 [M + Na]^+^ (calcd for C_23_H_47_N_2_O_9_NaPS_2_, 613.2358).

#### 1-(Phosphonomethyl)-4-(2-((2-(2-(vinylsulfonyl)ethoxy)ethyl)sulfonyl)ethyl)piperazine-1,4-Diium **VS-MP** (**17**)

A solution of HCl in Et_2_O (2 M, 3.00 mL, 6.00 mmol, 10.5 equiv) was added to a solution of **16** (169 mg, 0.28 mmol, 1.00 equiv) in MeOH (4 mL). The solution was stirred for 30 min at room temperature. The solvent was evaporated under reduced pressure to afford **17** (156 mg, 98%) as red syrup. ^1^H NMR (400 MHz, D_2_O): δ 6.88 (dd, *J* = 16.6, 10.1 Hz, 1H), 6.42 (d, *J* = 16.6 Hz, 1H), 6.32 (d, *J* = 10.0 Hz, 1H), 4.00–3.92 (m, 4H), 3.90–3.74 (m, 12H), 3.69 (ms, 4H), 3.60 (t, *J* = 5.0 Hz, 2H), 3.54–3.46 (m, 4H). ^13^C NMR (101 MHz, D_2_O): δ 135.62, 131.62, 69.62, 69.44, 63.54, 63.49, 53.53, 52.93 (d, *J* = 136.0 Hz), 50.34, 50.28, 49.15, 49.10, 48.47. ^31^P NMR (162 MHz, D_2_O): δ 6.15. IR (neat): ν 3370, 2923, 1632, 1287, 1119 cm^–1^. HR-MS (ESI^–^): *m*/*z*: 477.1129 [M – 3H – 2Cl]– (calcd for C_15_H_30_N_2_O_9_PS_2_, 477.1130).

### General Procedure for the Synthesis of PEI-BP-CD and PEI-MP-CD Ternary Conjugates (**20–22a,b**)

A solution of **β-CD-VS** (**19**, 4.00 equiv) in DMSO (2 mL) and Na_2_CO_3_ (5.00 equiv) was added to a solution of freshly lyophilized 2kPEI (1.00 equiv) in H_2_O (4 mL). The resulting solution was stirred for 3 days at room temperature. Subsequently, the corresponding **VS-BP** or **VS-MP** derivate (**7**, **11**, or **17**, 1.00 or 2.00 equiv) and the resulting solution were stirred for another 3 days at room temperature. The crude material was purified by dialysis (MWCO = 1000 Da) against water for 5 h and lyophilized to afford the corresponding **PEI-BP-CD** (**20–21a,b**) and **PEI-MP-CD** (**22a,b**). For individual conditions and details, see Table S2.

#### Nanoparticle Size

The nanoparticle size was determined using a Zetasizer μV instrument (Malvern) in 50 μL UV-transparent disposable cuvettes (Sarstedt). A 0.1 mg/mL suspension of nanoparticles (PEI-βCD and PEI-BP derivatives) was prepared in PBS, and the measurement was carried out at 25 °C using a refractive index of the sample of 1.53 and *A* = 0 and 3 cycles of 15 measurements of 10 s each.

### General Procedure for the Preparation of DOX ⊂ PEI-BP-CD and DOX ⊂ PEI-MP-CD Inclusion Complexes

DOX or ICG and **PEI-BP-CD** conjugates (**20–22a,b**) were incubated overnight at 4 °C (molar ratio *n*/*n*_CD_ = 0:9) to produce the corresponding inclusion complexes (**DOX ⊂ 20-22a,b**). After freeze-drying, the formation of the complexes was confirmed using UV–vis and fluorescence spectroscopy.

### *In Vitro* Biological Evaluation

#### Cell Culture

The cell lines were provided by the cell culture facility of the University of Granada. The MG-63 (ATCC CRL-1427), HeLa (ECACC 93021013), MDA-MB231 (ATCC HTB-26), and H9c2 (ATCC CRL-1446) cells were grown to reach 80–90% confluence at 37 °C in 5% CO_2_ in Dulbecco’s modified Eagle’s Medium and MC3T3-E1 cells (ECACC 99072810) were grown in minimum essential medium eagle, both supplemented with 10% (v/v) fetal bovine serum, 2 mM glutamine, 100 U/mL penicillin, and 0.1 mg/mL streptomycin (Sigma-Aldrich, Missouri, USA).

#### DOX Uptake Assay

The cells were seeded in a 24-well plate at a density of 4.5 × 10^5^ cells/well and, after 24 h, incubated for 1 h with 1 μM DOX or DOX equivalent amounts loaded into the βCD-based scaffolds. The fluorescence was measured after cell lysis with cell culture lysis reagent 1× (Promega, Madison, USA) at 499 nm (5 nm; excitation maximum wavelength) and 555 nm (10 nm; emission maximum wavelength) with a Shimadzu RF-5301PC fluorimeter and normalized for protein concentration, measured by a BCA assay.

#### Cell Cytotoxicity Assay

A total of 1.6 × 10^5^ cells/well were seeded in 48-well plates and incubated with 1 μM DOX or equivalent amounts of DOX-loaded into the βCD-based scaffolds. Cytotoxicity was evaluated after 48 h, using the 3-(4,5-dimethylthiazol-2-yl)-2,5-diphenyl-2*H*-tetrazolium bromide method. The results were calculated as the percentage of cell cytotoxicity based on the untreated control cells.

#### Western Blot Analysis

The MG-63 cells were incubated in the absence or presence of 15 μM PEI-BP or ALN for 30 min. The cells were lysed in 50 mM Hepes pH 7.5, 150 mM NaCl, 1% Nonidet P-40, 10 mM NaF, 20 mM NaPPi, 1 mM MgCl_2_, 1 mM CaCl_2_, 20 mM β-glycerophosphate, 2 mM sodium orthovanadate, 2 mM EDTA, 2 mM PMSF, and 4 μg/L leupeptin, and the lysate was centrifuged at 16,000×*g* for 15 min at 4 °C. Protein concentration on the supernatant was measured using a bicinchoninic acid kit (Bio-Rad, Madrid, Spain). The proteins were separated by SDS-PAGE and immunoblotted with selected antibodies. Specific antibodies against total and phospho (Ser473)-PKB/Akt and total and phospho (Thr202/Tyr204)-p44/42 MAPK (ERK1/2) (Cell Signaling, Beverly, MA, USA) were used.

#### Confocal Microscopy

For confocal analysis of cell internalization and localization of DOX and DOX loaded into the βCD-based scaffolds, the cells were seeded and grown for 24 h onto coverslips in 12-well plates at a density of 9 × 10^5^ cells/well, then incubated for 2 h with 1 μg/mL of DOX or DOX-loaded nanoparticles, and fixed with 2% paraformaldehyde in PBS for 15 min at room temperature. The MitoTracker Green FM (ThermoFischer Scientific, Waltham, Massachusetts, USA) was used as a marker of cell mitochondria and cholera toxin subunit B (Recombinant), Alexa Fluor 488 conjugate (ThermoFischer Scientific) as a marker of late endosomes. The cells were preincubated for 30 min before the addition of the compounds. The coverslips were mounted on glass slides using VECTASHIELD mounting media (Vector Laboratories, Inc., Burlingame, CA). Confocal microscopy was performed on a Leica TCS-SP5 II multiphoton confocal microscope. A sequential acquisition mode was used to separately collect the images in a single channel for color analysis. A pinhole of 1 Airy unit was used. Images were acquired at a resolution of 1024 Å ≈ 1024. Series were acquired in the xyz mode. Data were processed using the Leica AF software package.

#### Subcellular Fractionation

To study the subcellular distribution of DOX, 5 × 10^6^ of MG-63 cells/condition were treated for 2 h with DOX or DOX loaded into the β-CD-based scaffolds as described above. Mitochondria were isolated by a differential centrifugation method. Briefly, cells were washed and scraped with 1.5 mL of sterile ice-cold phosphate-buffered saline (PBS 1×, Sigma-Aldrich, Missouri, USA) and centrifuged 10 min at 800 × g. The pellet was homogenized with a mitochondrial isolation buffer (Tris HCl 50 mM pH 7.5; sucrose 250 mM, EDTA 1 mM) in a Teflon-glass homogenizer at the maximum speed for 10 passes. The cell lysate was centrifuged 10 min at 800 × g to remove cell debris and nuclei, and then, the supernatant was spun 10 min at 10 000 × g to obtain mitochondria-enriched pellet and a cytosolic fraction as the supernatant. The entire procedure was carried out under cold conditions (at 4 °C). Markers for each subcellular fraction were measured (Table S3). The fluorescence of DOX was measured in each cell fraction (cell lysate, nucleus, cytosol, and mitochondria) at its maximum excitation and emission wavelength and normalized for protein concentration in each sample.

#### Cytochrome *c* Release

MG-63 cells (3 × 10^6^/plate) were incubated with 2 μM DOX or **DOX ⊂ 23a** for either 24 or 48 h, and then, the mitochondrial and cytosolic fractions were isolated by the differential centrifugation method as previously described. The cytosolic release of cytochrome *c* was determined by Western blot of 5 μg of cytosolic and mitochondrial fractions, using cytochrome *c* antibody (Santa Cruz Biotechnology). Human GAPDH determination with GAPDH antibody (Santa Cruz Biotechnology) was used as a positive control.

#### ATP Synthesis

The synthesis of ATP in the mitochondrial fraction was measured using an ATP Determination Kit (A22066, ThermoFisher Scientific, Madrid, Spain) following the manufacturer’s instructions. The luminescence was determined using a Sirius L Tube Luminometer (Berthold Detection Systems). The amount of synthesized ATP was normalized by the protein concentration in each mitochondria sample (nmol ATP/mg protein).

### *In Vivo* Biological Evaluation

#### Uptake of Bisphosphonate-Based Compounds

Female NSG immunodeficient mice (6–8 weeks of age, 25–30 g weight) were purchased from the animal facility of the University of Granada and studied following guidelines established by Directive 2012/707/UE and the approval of the Committee on Animal Research at the University of Granada (03-02-15-186). For xenograft models, MDA-MB-231 or MG-63 cells were used. The cells were trypsinized and resuspended in PBS (density equal to 1 × 10^8^ cells/mL). The cells (5 × 10^6^) were injected intradermally into the flanks of each female NSG mouse. When tumor sizes reached 1 to 6 mm in diameter, the mice were injected in the tail vein with BP-based compounds containing 1 μM ICG occluded into the βCD (*n* = 4 for each experimental group). The *in vivo* imaging over time assays was performed in mice anesthetized with isofluorane. After 10 min, the animals were placed in a dark chamber for fluorescence (excitation/emission: 499/555 for DOX; 675/720 nm for ICG). Images were taken with an IVIS Spectrum (xCaliper Life Sciences, MA, USA) and analyzed with the Aura Imaging software 3.2 (Spectral Instruments Imaging, LLC).

#### Statistical Analysis

Results are expressed as mean ± SEM. Statistical analysis was performed by one-way ANOVA, followed by Tukey’s test as appropriate. *p* < 0.05 was considered statistically significant.

## Supporting Information Available

Molecular formula strings, individual conditions and details for the synthesis of PEI-BP-CD and PEI-MP-CD ternary systems (**20–22a,b**), spectroscopic characterization of novel and key compounds by NMR and HRMS experiments, particle size of the PEI-BP-CD ternary conjugates, subcellular fractionation of MG-63 cells, effects of inhibitors of internalization routes on **DOX ⊂ 21b** uptake, specific PEI-BP-CD (**20–21b**) and PEI-MP-CD (**22b**) conjugate uptake in bone cells, and *in vivo* imaging of tumor MDA-MB-231 xenografts in mice (PDF)
SMILES spreadsheet (XLSX)

## Abbreviations

ALN: alendronate
BPs: bisphosphonates
CD: cyclodextrin
DOX: doxorubicin
HA: hydroxyapatite
ICG: indocyanine green
MP: monophosphonate
PEI: polyethylenimine
TLC: thin-layer chromatography
VS: vinyl sulfone

## References

[ref1] del Castillo T.; Morales-Sanfrutos J.; Santoyo-González F.; Magez S.; Lopez-Jaramillo F. J.; Garcia-Salcedo J. A. Monovinyl Sulfone β-Cyclodextrin. A Flexible Drug Carrier System. ChemMedChem 2014, 9, 383–389. 10.1002/cmdc.201300385. 24339407

[ref2] Xing L.; Ebetino F. H.; Boeckman R. K. Jr.; Srinivasan V.; Tao J.; Sawyer T. K.; Li J.; Yao Z.; Boyce B. F. Targeting anti-cancer agents to bone using bisphosphonates. Bone 2020, 138, 115492 10.1016/j.bone.2020.115492. 32585321 PMC8485333

[ref3] Cole L. E.; Vargo-Gogola T.; Roeder R. K. Targeted delivery to bone and mineral deposits using bisphosphonate ligands. Adv. Drug Delivery Rev. 2016, 99, 12–27. 10.1016/j.addr.2015.10.005. 26482186

[ref4] Farrell K. B.; Karpeisky A.; Thamm D. H.; Zinnen S. Bisphosphonate conjugation for bone specific drug targeting. Bone Reports 2018, 9, 47–60. 10.1016/j.bonr.2018.06.007. 29992180 PMC6037665

[ref5] Barbosa J. S.; Almeida Paz F. A.; Braga S. S. Bisphosphonates, Old Friends of Bones and New Trends in Clinics. J. Med. Chem. 2021, 64, 1260–1282. 10.1021/acs.jmedchem.0c01292. 33522236

[ref6] Kuźnik A.; Październiok-Holewa A.; Jewula P.; Kuźnik N. Bisphosphonates-much more than only drugs for bone diseases. Eur. J. Pharmacol. 2020, 866, 172773 10.1016/j.ejphar.2019.172773. 31705903

[ref7] Cui Y.; Zhu T.; Li D.; Li Z.; Leng Y.; Ji X.; Liu H.; Wu D.; Ding J. Bisphosphonate-Functionalized Scaffolds for Enhanced Bone Regeneration. Adv. Healthcare Mater. 2019, 8, 1901073 10.1002/adhm.201901073. 31693315

[ref8] Hirabayashi H.; Fujisaki J. Bone-Specific Drug Delivery Systems. Clin. Pharmacokinet. 2003, 42, 1319–1330. 10.2165/00003088-200342150-00002. 14674786

[ref9] Webber M. J.; Langer R. Drug delivery by supramolecular design. Chem. Soc. Rev. 2017, 46, 6600–6620. 10.1039/c7cs00391a. 28828455

[ref10] Malhotra K.; Fuku R.; Chan T. S.; Kraljevic N.; Sedighi A.; Piunno P. A. E.; Krull U. J. Bisphosphonate Polymeric Ligands on Inorganic Nanoparticles. Chem. Mater. 2020, 32, 4002–4012. 10.1021/acs.chemmater.0c00547.

[ref11] Ossipov D. A. Bisphosphonate-modified biomaterials for drug delivery and bone tissue engineering. Expert Opin. Drug Delivery 2015, 12, 1443–1458. 10.1517/17425247.2015.1021679. 25739860

[ref12] Zhong Y.; Meng F.; Deng C.; Zhong Z. Ligand-Directed Active Tumor-Targeting Polymeric Nanoparticles for Cancer Chemotherapy. Biomacromolecules 2014, 15, 1955–1969. 10.1021/bm5003009. 24798476

[ref13] Santos A. C.; Costa D.; Ferreira L.; Guerra C.; Pereira-Silva M.; Pereira I.; Peixoto D.; Ferreira N. R.; Veiga F. Cyclodextrin-based delivery systems for in vivo-tested anticancer therapies. Drug Delivery Transl. Res. 2021, 11, 49–71. 10.1007/s13346-020-00778-5. 32441011

[ref14] Zhang J.; Ma P. X. Cyclodextrin-based supramolecular systems for drug delivery: Recent progress and future perspective. Adv. Drug Delivery Rev. 2013, 65, 1215–1233. 10.1016/j.addr.2013.05.001. PMC3885994 23673149

[ref15] Vicennati P.; Giuliano A.; Ortaggi G.; Masotti A. Polyethylenimine in medicinal chemistry. Curr. Med. Chem. 2008, 15, 2826–2839. 10.2174/092986708786242778. 18991638

[ref16] Calori I. R.; Braga G.; de Jesus P. d. C. C.; Bi H.; Tedesco A. C. Polymer scaffolds as drug delivery systems. Eur. Polym. J. 2020, 129, 109621 10.1016/j.eurpolymj.2020.109621.

[ref17] Low S. A.; Kopeček J. Targeting polymer therapeutics to bone. Adv. Drug Delivery Rev. 2012, 64, 1189–1204. 10.1016/j.addr.2012.01.012. PMC3349803 22316530

[ref18] Nadar R. A.; Margiotta N.; Iafisco M.; van den Beucken J. J. J. P.; Boerman O. C.; Leeuwenburgh S. C. G. Bisphosphonate-Functionalized Imaging Agents, Anti-Tumor Agents and Nanocarriers for Treatment of Bone Cancer. Adv. Healthcare Mater. 2017, 6, 1601119 10.1002/adhm.201601119. 28207199

[ref19] Kopeček J.; Yang J. Polymer nanomedicines. Adv. Drug Delivery Rev. 2020, 156, 40–64. 10.1016/j.addr.2020.07.020. PMC7736172 32735811

[ref20] Hrubý M.; Etrych T.; Kučka J.; Forsterová M.; Ulbrich K. Hydroxybisphosphonate-containing polymeric drug-delivery systems designed for targeting into bone tissue. J. Appl. Polym. Sci. 2006, 101, 3192–3201. 10.1002/app.23446.

[ref21] Ye W.-l.; Zhao Y.-p.; Li H.-q.; Na R.; Li F.; Mei Q.-b.; Zhao M.-g.; Zhou S.-y. Doxorubicin-poly (ethylene glycol)-alendronate self-assembled micelles for targeted therapy of bone metastatic cancer. Sci. Rep. 2015, 5, 14614 10.1038/srep14614. 26419507 PMC4588583

[ref22] Rudnick-Glick S.; Corem-Salkmon E.; Grinberg I.; Margel S. Targeted drug delivery of near IR fluorescent doxorubicin-conjugated poly(ethylene glycol) bisphosphonate nanoparticles for diagnosis and therapy of primary and metastatic bone cancer in a mouse model. J. Nanobiotechnol. 2016, 14, 80 10.1186/s12951-016-0233-6. PMC5139040 27919267

[ref23] Nair D. P.; Podgórski M.; Chatani S.; Gong T.; Xi W.; Fenoli C. R.; Bowman C. N. The Thiol-Michael Addition Click Reaction: A Powerful and Widely Used Tool in Materials Chemistry. Chem. Mater. 2014, 26, 724–744. 10.1021/cm402180t.

[ref24] Lopez-Jaramillo F. J.; Hernandez-Mateo F.; Santoyo-Gonzalez F. Vinyl Sulfone: A Multi-Purpose Function in Proteomics. Integrative Proteomics; 2012, pp 301−326. 10.5772/29682.

[ref25] Morales-Sanfrutos J.; Lopez-Jaramillo J.; Ortega-Muñoz M.; Megia-Fernandez A.; Perez-Balderas F.; Hernandez-Mateo F.; Santoyo-Gonzalez F. Vinyl sulfone: a versatile function for simple bioconjugation and immobilization. Org. Biomol. Chem. 2010, 8, 667–675. 10.1039/b920576d. 20090986

[ref26] Wang X.; Niu D.; Hu C.; Li P. Polyethyleneimine-Based Nanocarriers for Gene Delivery. Curr. Pharm. Des. 2015, 21, 6140–6156. 10.2174/1381612821666151027152907. 26503146

[ref27] Zakeri A.; Kouhbanani M. A. J.; Beheshtkhoo N.; Beigi V.; Mousavi S. M.; Hashemi S. A. R.; Karimi Zade A.; Amani A. M.; Savardashtaki A.; Mirzaei E.; Jahandideh S.; Movahedpour A. Polyethylenimine-based nanocarriers in co-delivery of drug and gene: a developing horizon. Nano Rev. Exp. 2018, 9, 1488497 10.1080/20022727.2018.1488497. 30410712 PMC6171788

[ref28] Zeevaart J. R.; Louw W. K. A.; Kolar Z. I.; Wagener J. M.; Jarvis N. V.; Claessens R. A. M. J. A thermodynamic approach, using speciation studies, towards the evaluation and design of bone-seeking radiopharmaceuticals as illustrated for 117mSn(II)-PEI-MP. J. Radioanal. Nucl. Chem. 2003, 257, 83–91. 10.1023/a:1024745310042.

[ref29] Jansen D. R.; Krijger G. C.; Wagener J.; Senwedi R. M.; Gabanamotse K.; Kgadiete M.; Kolar Z. I.; Zeevaart J. R. Blood plasma model predictions for the proposed bone-seeking radiopharmaceutical [117mSn]Sn(IV)-N,N′,N′-trimethylenephosphonate-poly(ethyleneimine). J. Inorg. Biochem. 2009, 103, 1265–1272. 10.1016/j.jinorgbio.2009.07.007. 19665234

[ref30] Jansen D. R.; Rijn Zeevaart J.; Denkova A.; Kolar Z. I.; Krijger G. C. Hydroxyapatite Chemisorption of N,N′,N′-Trimethylenephosphonate–Poly(ethyleneimine) (PEI–MP) Combined with Sn2+ or Sn4+. Langmuir 2009, 25, 2790–2796. 10.1021/la802485g. 19437756

[ref31] Harding I. S.; Rashid N.; Hing K. A. Surface charge and the effect of excess calcium ions on the hydroxyapatite surface. Biomaterials 2005, 26, 6818–6826. 10.1016/j.biomaterials.2005.04.060. 15955555

[ref32] Brunot C.; Ponsonnet L.; Lagneau C.; Farge P.; Picart C.; Grosgogeat B. Cytotoxicity of polyethyleneimine (PEI), precursor base layer of polyelectrolyte multilayer films. Biomaterials 2007, 28, 632–640. 10.1016/j.biomaterials.2006.09.026. 17049374

[ref33] Cole L. E.; Vargo-Gogola T.; Roeder R. K. Targeted delivery to bone and mineral deposits using bisphosphonate ligands. Adv. Drug Delivery Rev. 2016, 99, 12–27. 10.1016/j.addr.2015.10.005. 26482186

[ref34] Russell R. G. G. Bisphosphonates: The first 40years. Bone 2011, 49, 2–19. 10.1016/j.bone.2011.04.022. 21555003

[ref35] Rivankar S. An overview of doxorubicin formulations in cancer therapy. J. Canc. Res. Therapeut. 2014, 10, 853–858. 10.4103/0973-1482.139267. 25579518

[ref36] Zhao L.; Shen G.; Ma G.; Yan X. Engineering and delivery of nanocolloids of hydrophobic drugs. Adv. Colloid Interface Sci. 2017, 249, 308–320. 10.1016/j.cis.2017.04.008. 28456289

[ref37] Bishop M. W.; Janeway K. A.; Gorlick R. Future Directions in the Treatment of Osteosarcoma. Curr. Opin. Pediatr. 2016, 1, 26 10.1097/MOP.0000000000000298. PMC4761449 26626558

[ref38] Petrioli R.; Fiaschi A. I.; Francini E.; Pascucci A.; Francini G. The role of doxorubicin and epirubicin in the treatment of patients with metastatic hormone-refractory prostate cancer. Canc. Treat Rev. 2008, 34, 710–718. 10.1016/j.ctrv.2008.05.004. 18620815

[ref39] Ottewell P. D.; Woodward J. K.; Lefley D. V.; Evans C. A.; Coleman R. E.; Holen I. Anticancer mechanisms of doxorubicin and zoledronic acid in breast cancer tumor growth in bone. Mol. Cancer Ther. 2009, 8, 2821–2832. 10.1158/1535-7163.mct-09-0462. 19789217

[ref40] Hochdörffer K.; Abu Ajaj K.; Schäfer-Obodozie C.; Kratz F. Development of novel bisphosphonate prodrugs of doxorubicin for targeting bone metastases that are cleaved pH dependently or by cathepsin B: synthesis, cleavage properties, and binding properties to hydroxyapatite as well as bone matrix. J. Med. Chem. 2012, 55, 7502–7515. 10.1021/jm300493m. 22882004

[ref41] David E.; Cagnol S.; Goujon J.-Y.; Egorov M.; Taurelle J.; Benesteau C.; Morandeau L.; Moal C.; Sicard M.; Pairel S.; Heymann D.; Redini F.; Gouin F.; Le Bot R. 12b80 - Hydroxybisphosphonate Linked Doxorubicin: Bone Targeted Strategy for Treatment of Osteosarcoma. Bioconjugate Chem. 2019, 30, 1665–1676. 10.1021/acs.bioconjchem.9b00210. 31045351

[ref42] Bekers O.; Beijnen J. H.; Otagiri M.; Bult A.; Underberg W. J. M. Inclusion complexation of doxorubicin and daunorubicin with cyclodextrins. J. Pharm. Biomed. Anal. 1990, 8, 671 10.1016/0731-7085(90)80100-4. 2100605

[ref43] Liu X.-M.; Lee H.-T.; Reinhardt R. A.; Marky L. A.; Wang D. Novel biomineral-binding cyclodextrins for controlled drug delivery in the oral cavity. J. Controlled Release 2007, 122, 54–62. 10.1016/j.jconrel.2007.06.021. 17673326

[ref44] Hein C. D.; Liu X.-M.; Chen F.; Cullen D. M.; Wang D. The Synthesis of a Multiblock Osteotropic Polyrotaxane by Copper(I)-Catalyzed Huisgen 1,3-Dipolar Cycloaddition. Macromol. Biosci. 2010, 10, 1544–1556. 10.1002/mabi.201000205. 20954201

[ref45] Luckman S. P.; Hughes D. E.; Coxon F. P.; Russell R. G. G.; Rogers M. J.; Rogers M. J. Nitrogen-containing bisphosphonates inhibit the mevalonate pathway and prevent post-translational prenylation of GTP-binding proteins, including Ras. J. Bone Miner. Res. 1998, 13, 581–589. 10.1359/jbmr.1998.13.4.581. 9556058

[ref46] Quarles L. D.; Yohay D. A.; Lever L. W.; Caton R.; Wenstrup R. J. Distinct proliferative and differentiated stages of murine MC3T3-E1 cells in culture: an in vitro model of osteoblast development. J. Bone Miner. Res. 1992, 7, 683–92. 10.1002/jbmr.5650070613. 1414487

[ref47] Chang J.; Wang W.; Zhang H.; Hu Y.; Yin Z. Bisphosphonates regulate cell proliferation, apoptosis and pro-osteoclastic expression in MG-63 human osteosarcoma cells. Oncol. Lett. 2012, 4, 299–304. 10.3892/ol.2012.723. 22844373 PMC3402760

[ref48] Sun J.; Song F.; Zhang W.; Sexton B. E.; Windsor L. J. Effects of alendronate on human osteoblast-like MG63 cells and matrix metalloproteinases. Arch. Oral Biol. 2012, 57, 728–736. 10.1016/j.archoralbio.2011.12.007. 22251575

[ref49] Zhang S.; Wright J. E. I.; Özber N.; Uludağ H. The interaction of cationic polymers and their bisphosphonate derivatives with hydroxyapatite. Macromol. Biosci. 2007, 7, 656–670. 10.1002/mabi.200600286. 17457941

[ref50] Lee K.; Seo I.; Choi M. H.; Jeong D. Roles of Mitogen-Activated Protein Kinases in Osteoclast Biology. Int. J. Mol. Sci. 2018, 19, 3004 10.3390/ijms19103004. PMC6213329 30275408

[ref51] Tsubaki M.; Komai M.; Itoh T.; Imano M.; Sakamoto K.; Shimaoka H.; Takeda T.; Ogawa N.; Mashimo K.; Fujiwara D.; Mukai J.; Sakaguchi K.; Satou T.; Nishida S. Nitrogen-containing bisphosphonates inhibit RANKL- and M-CSF-induced osteoclast formation through the inhibition of ERK1/2 and Akt activation. J. Biomed. Sci. 2014, 21, 10 10.1186/1423-0127-21-10. 24490900 PMC3996180

[ref52] Carpio L.; Gladu J.; Goltzman D.; Rabbani S. A. Induction of osteoblast differentiation indexes by PTHrP in MG-63 cells involves multiple signaling pathways. Am. J. Physiol. Endocrinol. Metab. 2001, 281, E489–E499. 10.1152/ajpendo.2001.281.3.e489. 11500304

[ref53] Inoue R.; Matsuki N.-a.; Jing G.; Kanematsu T.; Abe K.; Hirata M. The inhibitory effect of alendronate, a nitrogen-containing bisphosphonate on the PI3K-Akt-NFκ B pathway in osteosarcoma cells. Br. J. Pharmacol. 2005, 146, 633–641. 10.1038/sj.bjp.0706373. 16100524 PMC1751194

[ref54] Ebert R.; Meissner-Weigl J.; Zeck S.; Määttä J.; Auriola S.; Coimbra de Sousa S.; Mentrup B.; Graser S.; Rachner T. D.; Hofbauer L. C.; Jakob F. Probenecid as a sensitizer of bisphosphonate-mediated effects in breast cancer cells. Mol. Canc. 2014, 13, 265 10.1186/1476-4598-13-265. PMC4295226 25496233

[ref55] Hiraga T.; Williams P. J.; Mundy G. R.; Yoneda T. The bisphosphonate ibandronate promotes apoptosis in MDA-MB-231 human breast cancer cells in bone metastases. Cancer Res. 2001, 61, 4418–24. 11389070

[ref56] Bao K.; Nasr K. A.; Hyun H.; Lee J. H.; Gravier J.; Gibbs S. L.; Choi H. S. Charge and hydrophobicity effects of NIR fluorophores on bone-specific imaging. Theranostics 2015, 5, 609–617. 10.7150/thno.11222. 25825600 PMC4377729

[ref57] Sebbah-Louriki M.; Colombo B. M.; el Manouni D.; Martin A.; Salzmann J. L.; Leroux Y.; Perret G. Y.; Crépin M. A new phenylacetate-bisphosphonate inhibits breast cancer cell growth by proapoptotic and antiangiogenic effects. Anticancer Res. 2002, 22, 3925–31. 12553014

[ref58] Svoboda M.; Riha J.; Wlcek K.; Jaeger W.; Thalhammer T. Organic anion transporting polypeptides (OATPs): regulation of expression and function. Curr. Drug Metab. 2011, 12, 139–153. 10.2174/138920011795016863. 21395542

[ref59] Chandra P.; Zhang P.; Brouwer K. L. R. Short-term regulation of multidrug resistance-associated protein 3 in rat and human hepatocytes. Am. J. Physiol. Gastrointest. Liver Physiol. 2005, 288, G1252–G1258. 10.1152/ajpgi.00362.2004. 15650133

[ref60] Coley H.; Amos W.; Twentyman P.; Workman P. Examination by laser scanning confocal fluorescence imaging microscopy of the subcellular localisation of anthracyclines in parent and multidrug resistant cell lines. Br. J. Cancer 1993, 67, 1316–1323. 10.1038/bjc.1993.244. 8099807 PMC1968480

[ref61] Riganti C.; Rolando B.; Kopecka J.; Campia I.; Chegaev K.; Lazzarato L.; Federico A.; Fruttero R.; Ghigo D. Mitochondrial-targeting nitrooxy-doxorubicin: a new approach to overcome drug resistance. Mol. Pharm. 2013, 10, 161–174. 10.1021/mp300311b. 23186264

[ref62] Buondonno I.; Gazzano E.; Jean S. R.; Audrito V.; Kopecka J.; Fanelli M.; Salaroglio I. C.; Costamagna C.; Roato I.; Mungo E.; Hattinger C. M.; Deaglio S.; Kelley S. O.; Serra M.; Riganti C. Mitochondria-Targeted Doxorubicin: A New Therapeutic Strategy against Doxorubicin-Resistant Osteosarcoma. Mol. Cancer Ther. 2016, 15, 2640–2652. 10.1158/1535-7163.mct-16-0048. 27466354

[ref63] Marshall M. V.; Rasmussen J. C.; Tan I.-C.; Aldrich M. B.; Adams K. E.; Wang X.; Fife C. E.; Maus E. A.; Smith L. A.; Sevick-Muraca E. M. Near-Infrared Fluorescence Imaging in Humans with Indocyanine Green: A Review and Update. Open Surg. Oncol. J. 2010, 2, 12–25. 10.2174/1876504101002010012. 22924087 PMC3424734

[ref64] Hallett A. J.; Christian P.; Jones J. E.; Pope S. J. A. Luminescent, water-soluble gold nanoparticles functionalised with 3MLCT emitting rhenium complexes. Chem. Commun. 2009, 4278–4280. 10.1039/b905692k. 19585046

[ref65] Mori M.; Dasso Lang M. C.; Saladini F.; Palombi N.; Kovalenko L.; De Forni D.; Poddesu B.; Friggeri L.; Giannini A.; Malancona S.; Summa V.; Zazzi M.; Mely Y.; Botta M. Synthesis and Evaluation of Bifunctional Aminothiazoles as Antiretrovirals Targeting the HIV-1 Nucleocapsid Protein. ACS Med. Chem. Lett. 2019, 10, 463–468. 10.1021/acsmedchemlett.8b00506. 30996780 PMC6466545

[ref66] Harris R. K.; Becker E. D.; Cabral de Menezes S. M.; Goodfellow R.; Granger P. NMR nomenclature. Nuclear spin properties and conventions for chemical shifts(IUPAC Recommendations 2001). Pure Appl. Chem. 2001, 73, 1795–1818. 10.1351/pac200173111795. 12637147

[ref67] Prishchenko A. A.; Livantsov M. V.; Shagi-Mukhametova N. M.; Petrosyan V. S. Synthesis of tetraisopropyl vinylidenediphosphonate. Zh. Obshch. Khim. 1991, 61, 1018.

