# Supplementary material for: Polyethylenimine–Bisphosphonate–Cyclodextrin Ternary Conjugates: Supramolecular Systems for the Delivery of Antineoplastic Drugs
Source: J Med Chem. 2021 Aug 9;64(16):12245–60. doi: 10.1021/acs.jmedchem.1c00887 (PMC8477368; doi:10.1021/acs.jmedchem.1c00887)
Supplement: Supplementary file 1 — jm1c00887_si_001.pdf [file jm1c00887_si_001.pdf]

SUPPORTING INFORMATION

Polyethylenimine-Bisphosphonate-Cyclodextrin Ternary Conjugates: Supramolecular Systems for the Delivery of Antineoplastic Drugs

Simona Plesselova,[a,d,†] Pablo Garcia-Cerezo,[b,d,†] Victor Blanco,[b,d] Francisco J. Reche-Perez,[a,d] Fernando Hernandez-Mateo,[b,c,d] Francisco Santoyo-Gonzalez,[b,c,d*] María Dolores Giron-Gonzalez,[a,d] and Rafael Salto-Gonzalez[a,d*]

[a]Department of Biochemistry and Molecular Biology II, School of Pharmacy, University of Granada, E-18071 Granada, Spain
[b]Department of Organic Chemistry, School of Sciences, University of Granada, E-18071 Granada, Spain
[c]Biotechnology Institute, University of Granada, E-18071 Granada, Spain
[d]Unit of Excellence in Chemistry Applied to Biomedicine and the Environment of the University of Granada, E-18071 Granada, Spain

Corresponding Authors e-mails: rsalto@ugr.es; fsantoyo@ugr.es

List of Contents

1. **Synthesis and characterization of PEI-BP-CD (20-21a,b) and PEI-MP-CD (22a.b) ternary systems** **S3**

2. **NMR Spectra** **S4**

3. **HRMS Spectra of key Products** **S35**

5. **Particle size of the PEI-BP-CD ternary conjugates** **S42**

6. **Subcellular fractionation of MG-63 cells** **S43**

7. **Effects of inhibitors of internalization routes on DOX⊂21b uptake** **S44**

8. **Specific PEI-BP-CD (20-21b) and PEI-MP-CD (22b) conjugates uptake in bone cells** **S45**

9. ***In vivo* imaging of tumor MDA-MB-231 xenografts in mice** **S46**

1. Synthesis of PEI-BP-CD (20-21a,b) and PEI-MP-CD (22a,b) ternary systems

Table S1. Individual Conditions.

Entry	2kPEI (μmol)	Phosphonate (μmol)	β-CD-VS (μmol)	Compound(mg)
**1**	12.5	**7** (12.5)	50.0	**20a (**95)
**2**	12.5	**7** (25.0)	50.0	**20b (**47)
**3**	12.5	**11** (12.5)	50.0	**21a (**76)
**4**	12.5	**11** (25.0)	50.0	**21b (**82)
**5**	7.5	**17** (7.5)	30.0	**22a (**54)
**6**	7.5	**17** (15.0)	30.0	**22b (**57)

Compound 20a. White foam. ^{31}P NMR (202 MHz, D_2O/DMSO-d_6): δ=17.73. IR (neat): ν= 2969, 1740, 1367, 1216, 1026 cm^{-1}.

Compound 20b. White foam. ^{31}P NMR (162 MHz, D_2O/DMSO-d_6): δ=17.75. IR (neat): ν= 2932, 1740, 1367, 1216, 1026 cm^{-1}.

Compound 21a. White solid. ^{31}P NMR (202 MHz, D_2O/DMSO-d_6): δ=6.50. IR (neat): ν=3282, 2930, 1285, 1079, 1022 cm^{-1}.

Compound 21b. White solid. ^{31}P NMR (202 MHz, D_2O/DMSO-d_6): δ=6.89. IR (neat): ν= 3282, 2930, 1739, 1367, 1079, 1026 cm^{-1}.

Compound 22a. Light yellow solid. ^{31}P NMR (202 MHz, D_2O/DMSO-d_6): δ=11.50. IR (neat): ν= 3271, 2923, 1738, 1365, 1078, 1024 cm^{-1}.

Compound 22b. Light yellow solid. ^{31}P NMR (202 MHz, D_2O/DMSO-d_6): δ=11.01. IR (neat): ν= 2914, 1740, 1367, 1080, 1029 cm^{-1}.

3. NMR spectra

Compound 2:

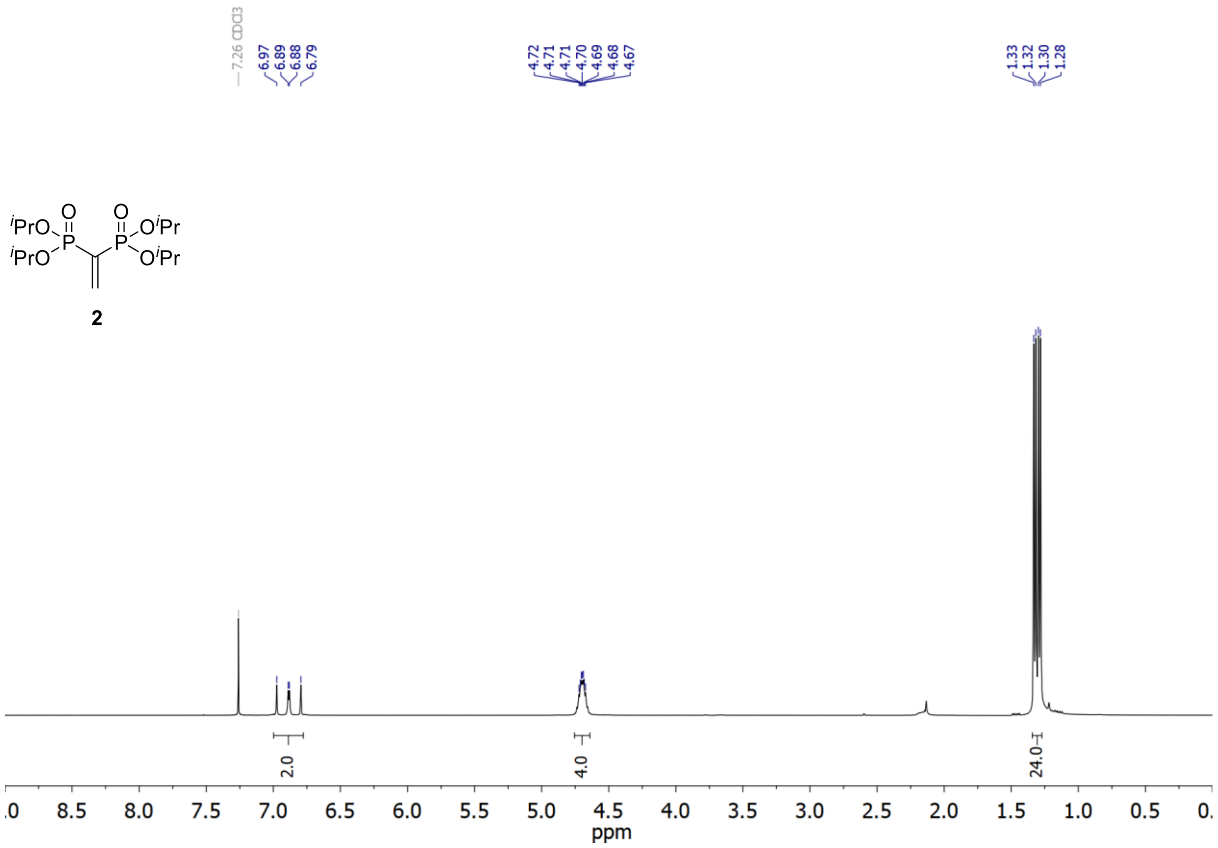

Figure S1. ^{1}H NMR (400 MHz, $CDCl_3$) spectrum of **2**.

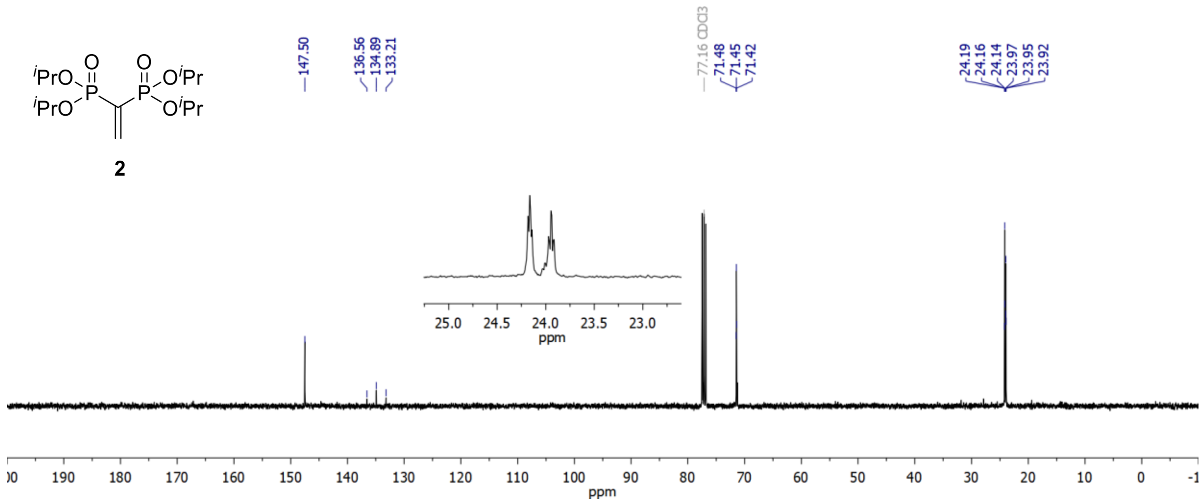

Figure S2. ^{13}C NMR (101 MHz, $CDCl_3$) spectrum of **2**.

Compound 4:

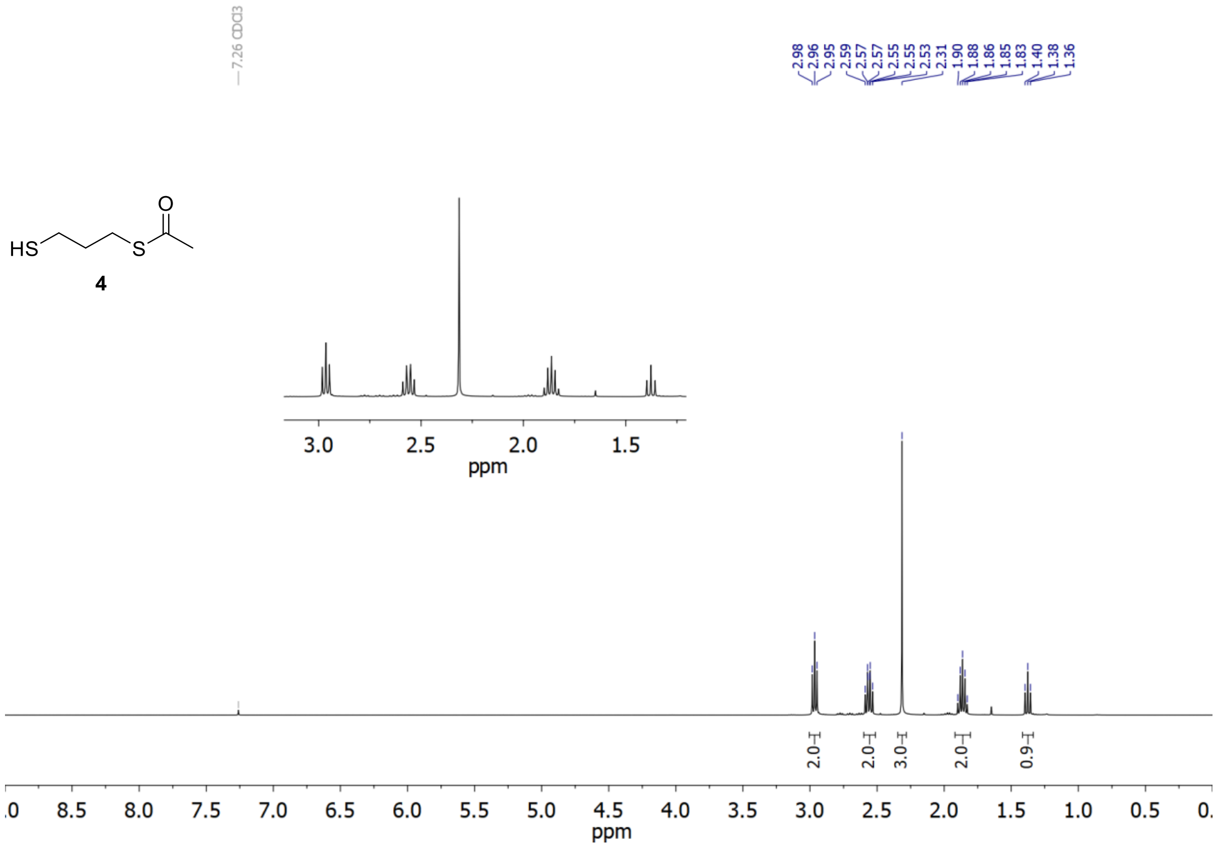

Figure S3. ^{1}H NMR (400 MHz, $CDCl_3$) spectrum of **4**.

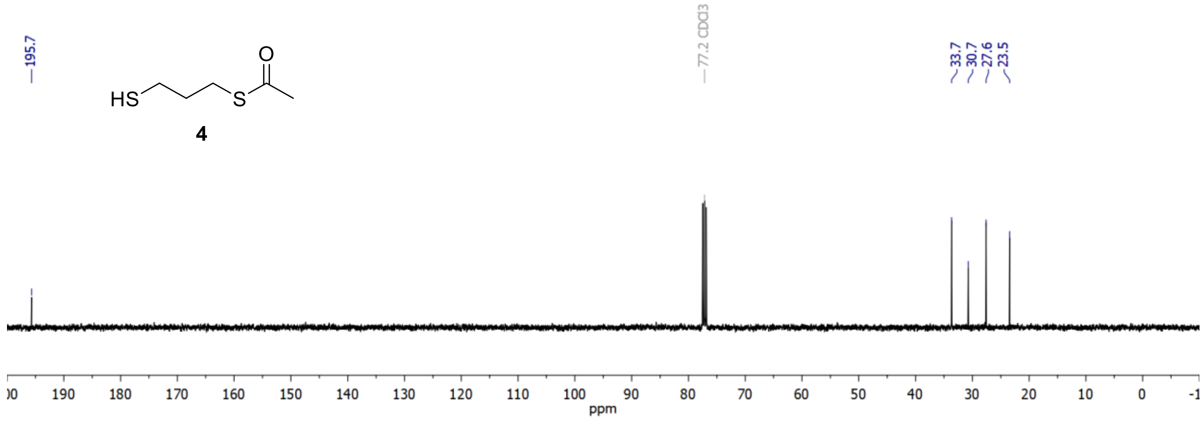

Figure S4. ^{13}C NMR (101 MHz, $CDCl_3$) spectrum of **4**.

Compound 5:

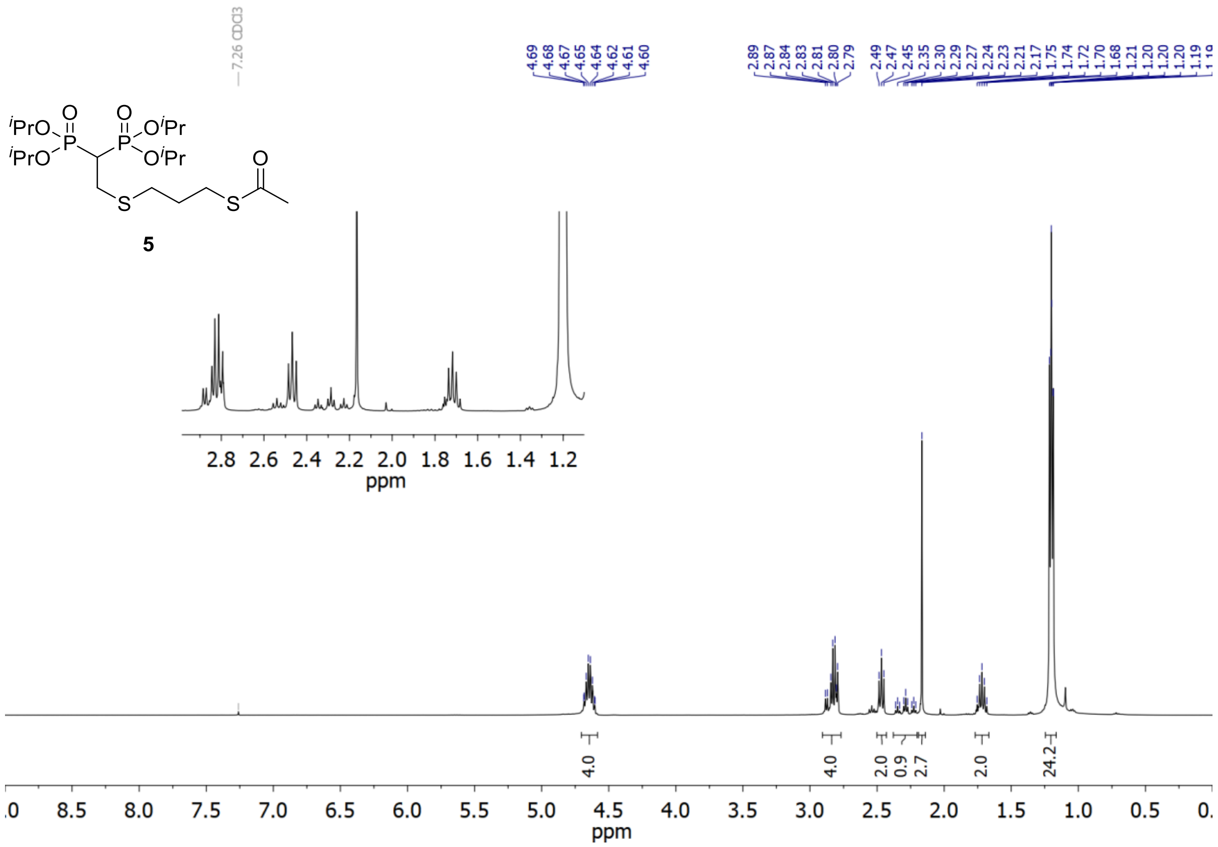

Figure S5. ^{1}H NMR (400 MHz, $CDCl_3$) spectrum of **5**.

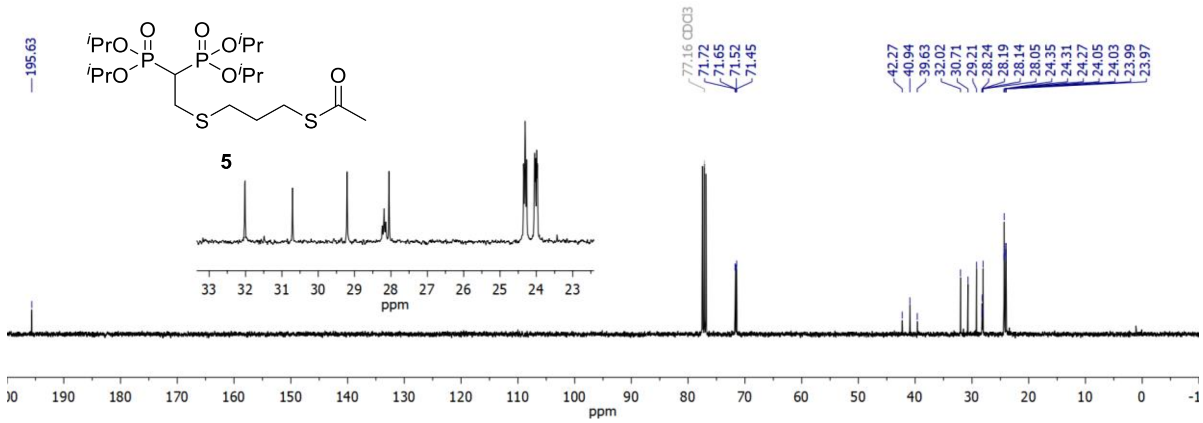

Figure S6. ^{13}C NMR (101 MHz, $CDCl_3$) spectrum of **5**.

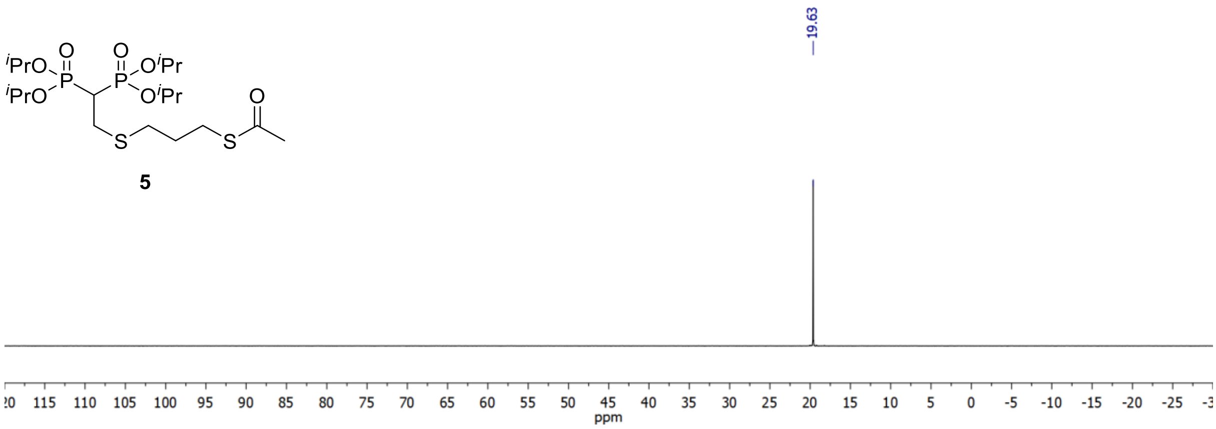

Figure S7. ^{31}P NMR (162 MHz, $CDCl_3$) spectrum of **5**.

Compound 6:

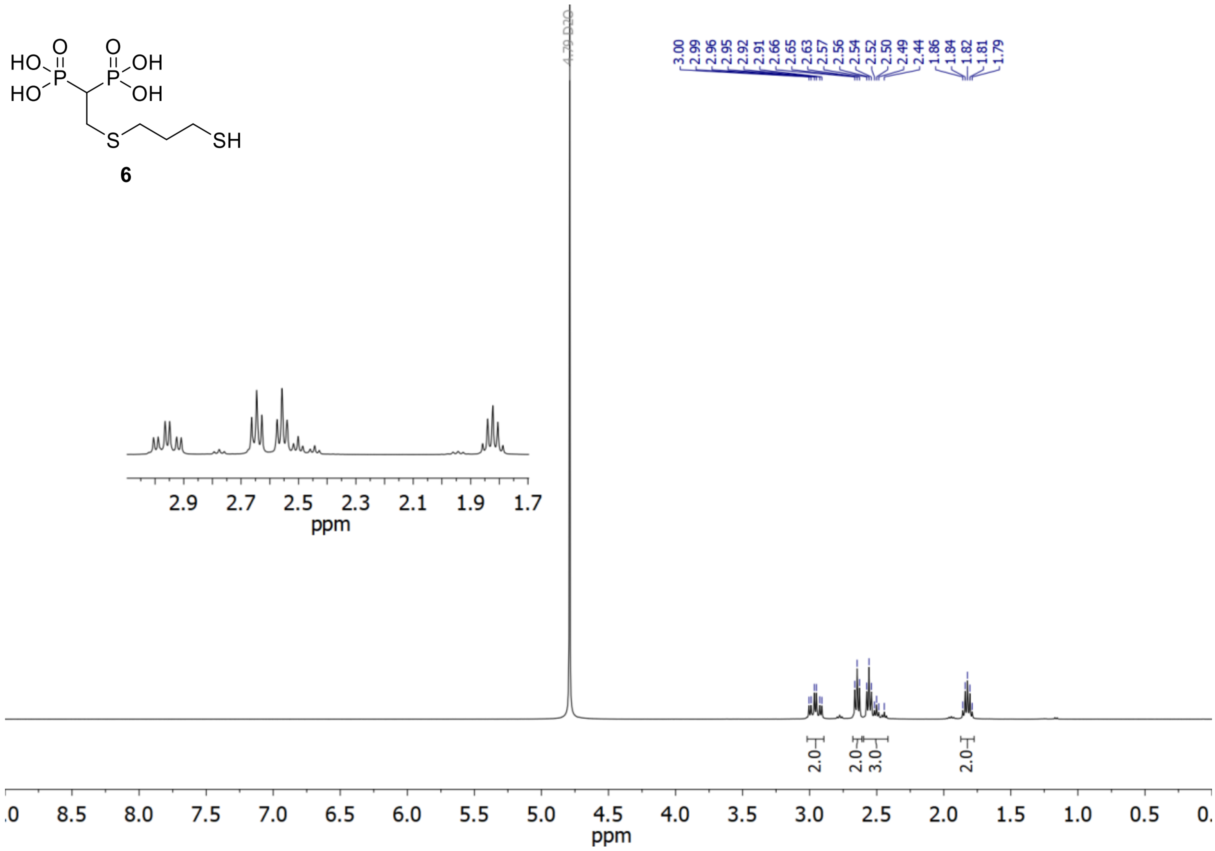

Figure S8. ^{1}H NMR (400 MHz, D_2O) spectrum of **6**.

Compound 7:

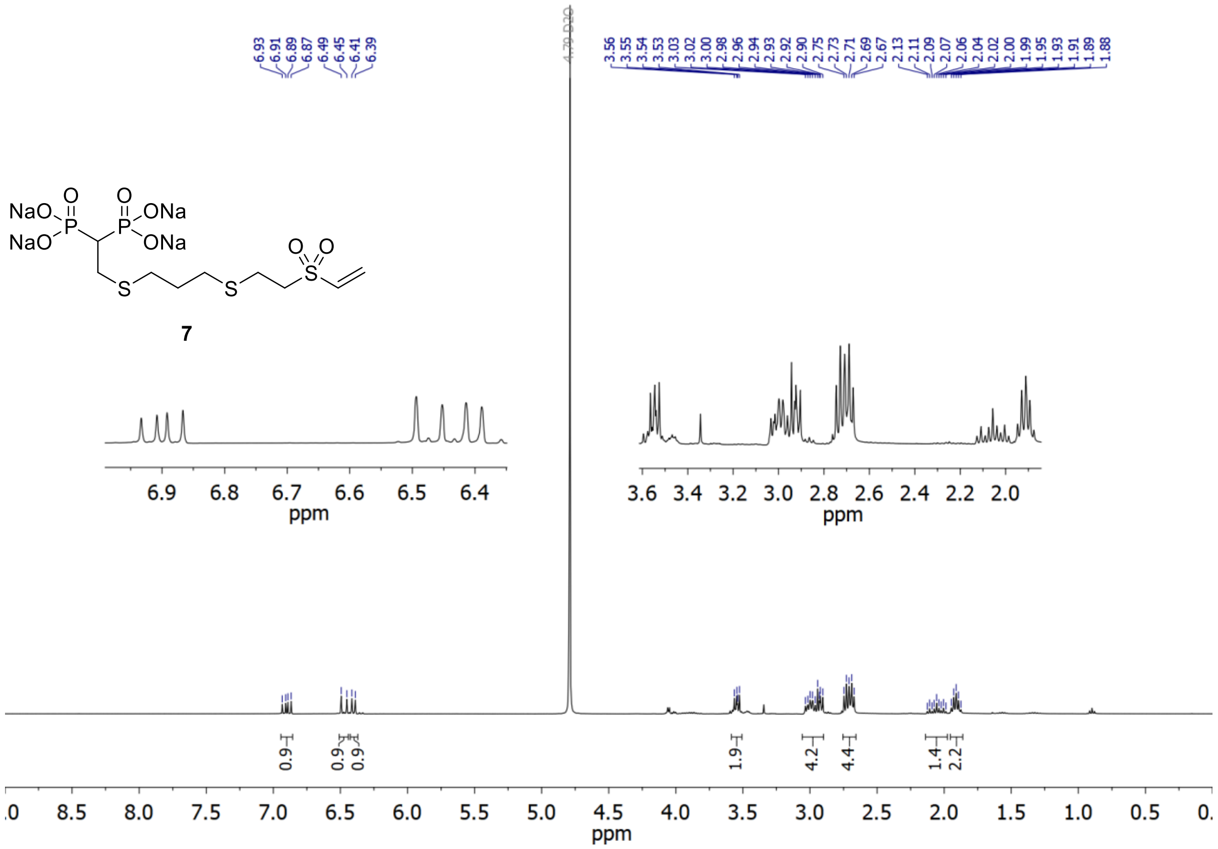

Figure S9. ^{1}H NMR (400 MHz, D_2O) spectrum of **7**.

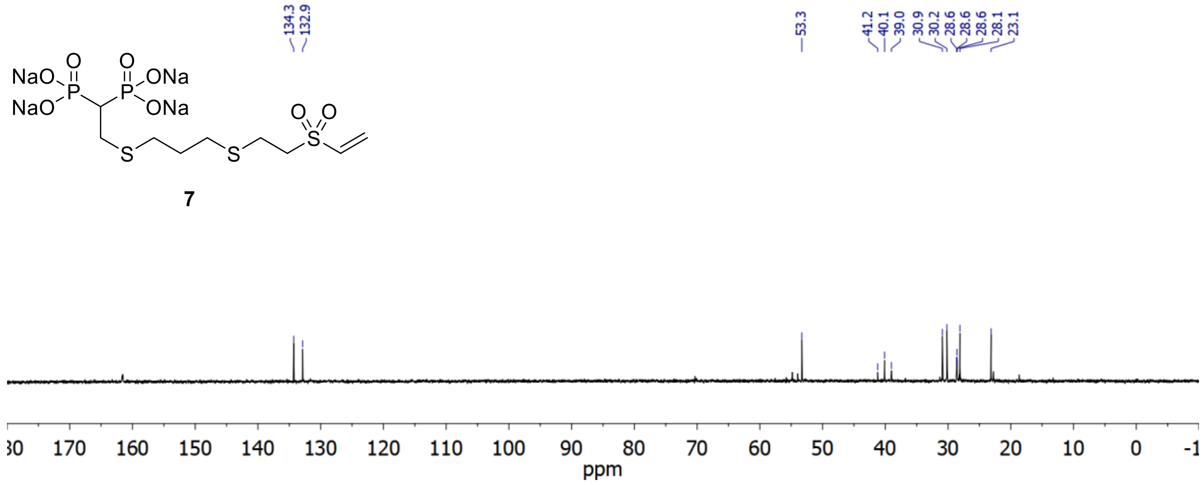

Figure S10. ^{13}C NMR (101 MHz, D_2O) spectrum of **7**.

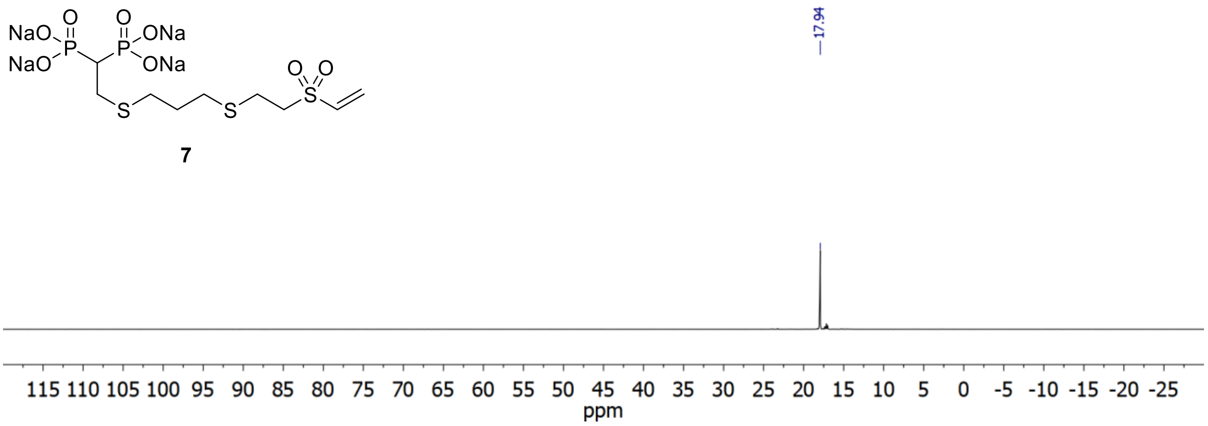

Figure S11. ^{31}P NMR (162 MHz, D_2O) spectrum of **7**.

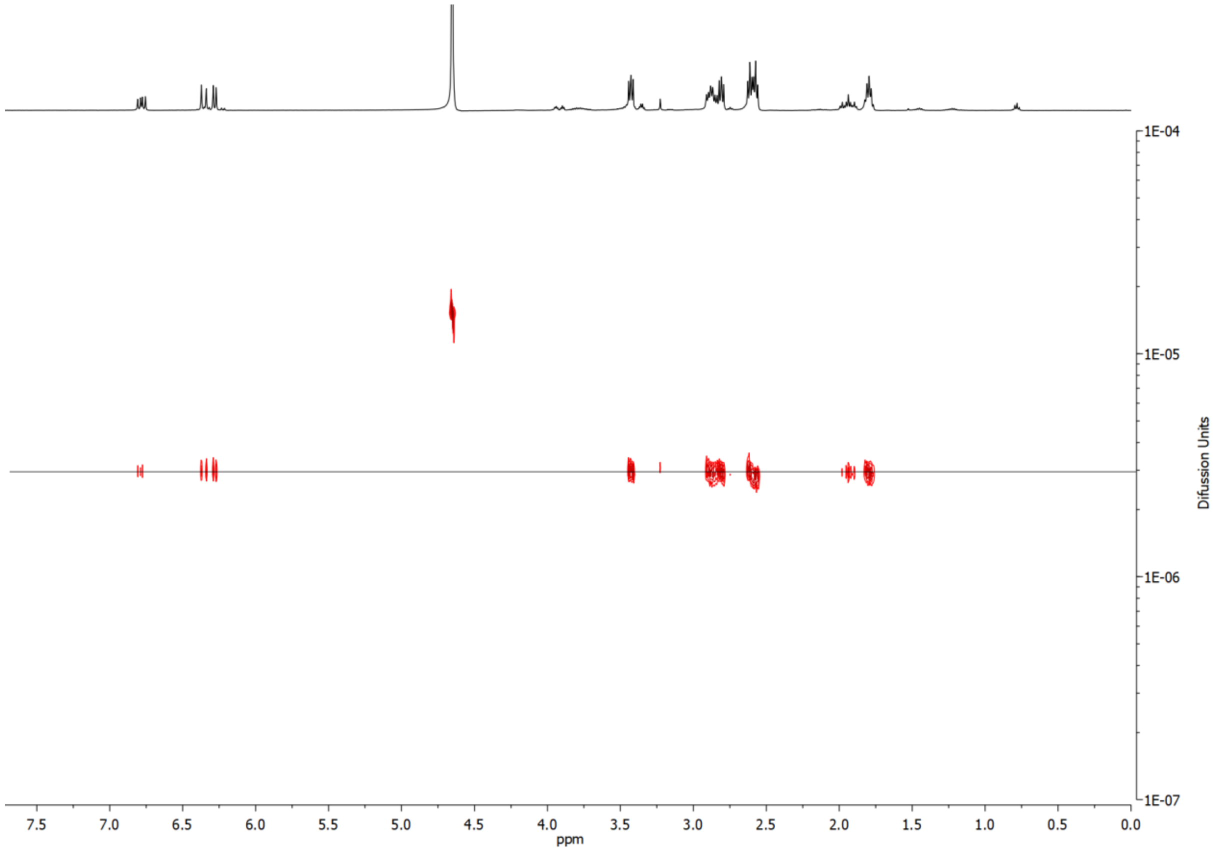

Figure S12. DOSY NMR (500 MHz, D_2O) spectrum of **7**.

Compound 9:

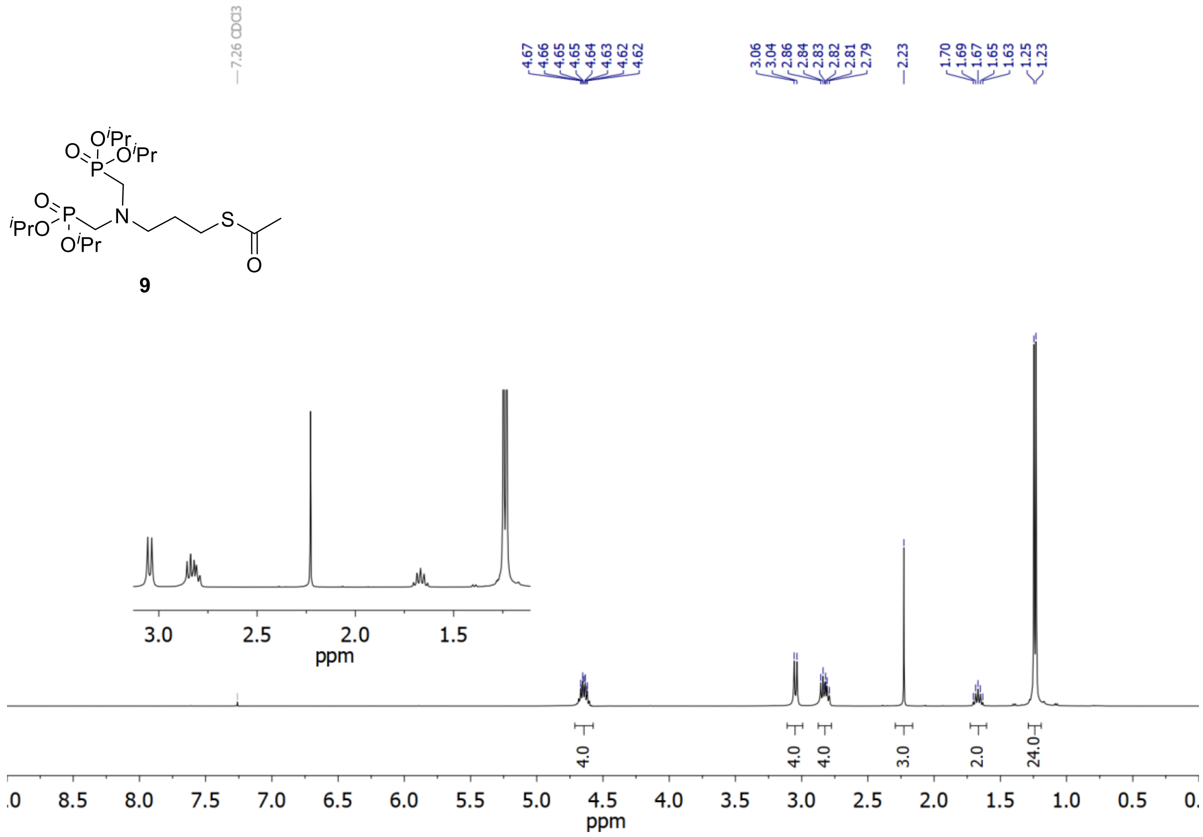

Figure S13. ^{1}H NMR (400 MHz, $CDCl_3$) spectrum of **9**.

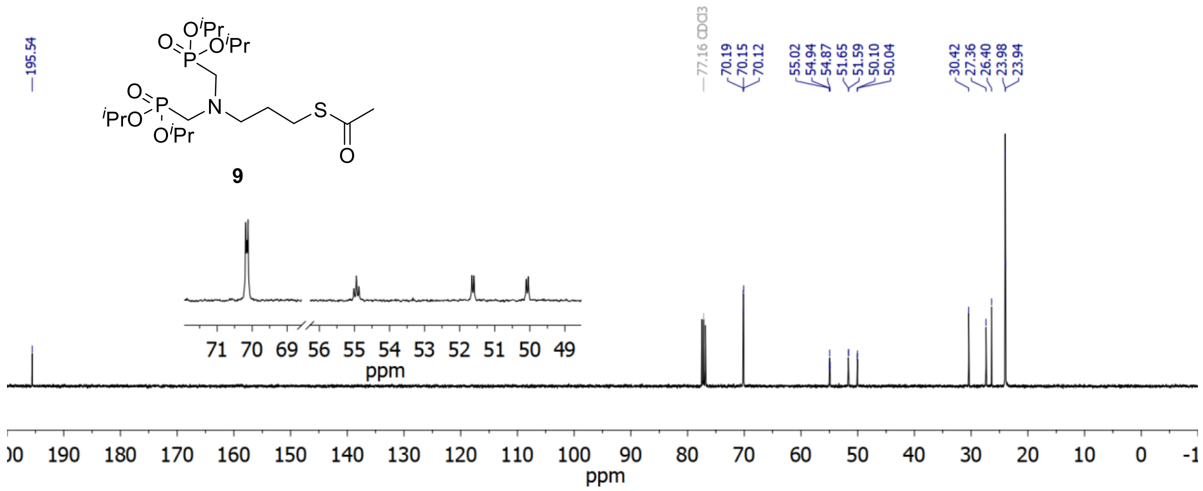

Figure S14. ^{13}C NMR (101 MHz, $CDCl_3$) spectrum of **9**.

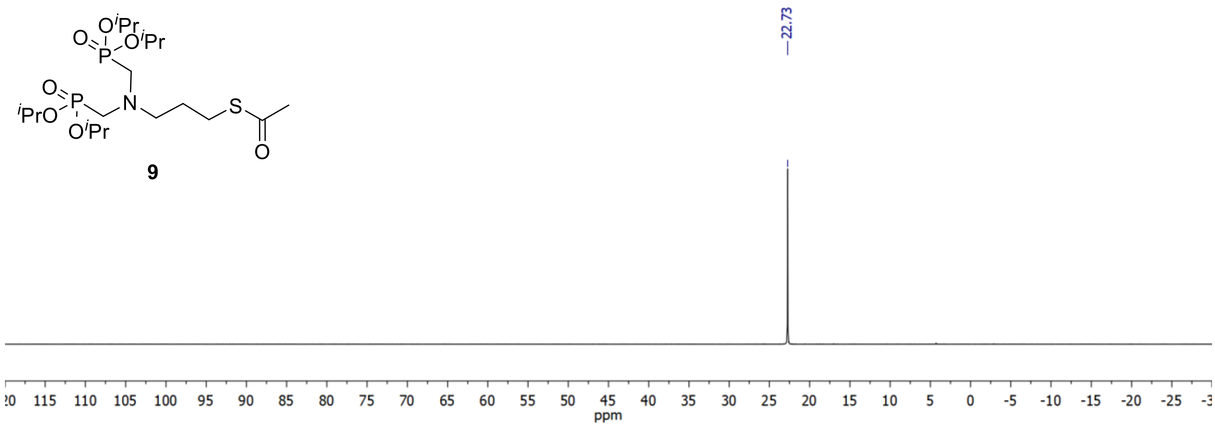

Figure S15. ^{31}P NMR (162 MHz, $CDCl_3$) spectrum of **9**.

Compound 10:

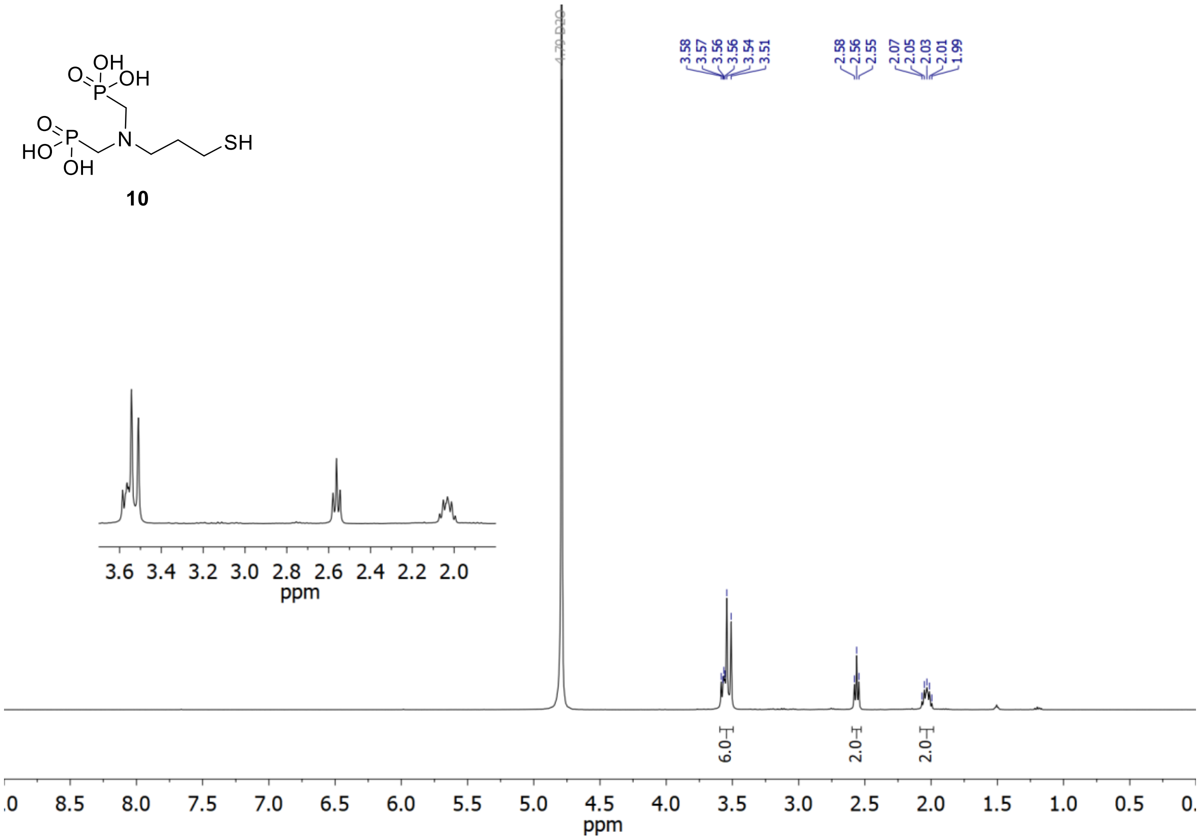

Figure S16. ^{1}H NMR (400 MHz, D_2O) spectrum of **10**.

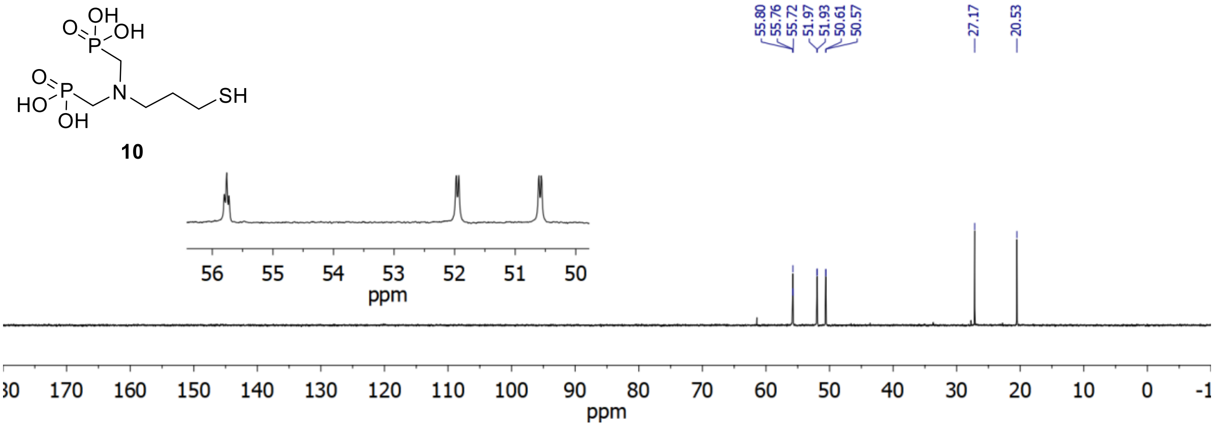

Figure S17. ^{13}C NMR (101 MHz, D_2O) spectrum of **10**.

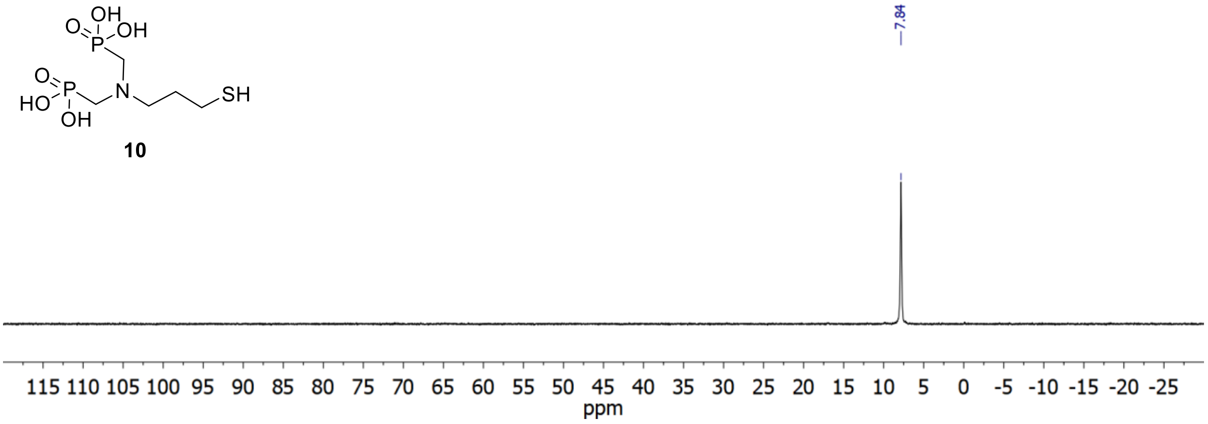

Figure S18. ^{31}P NMR (162 MHz, D_2O) spectrum of **10**.

Compound 11:

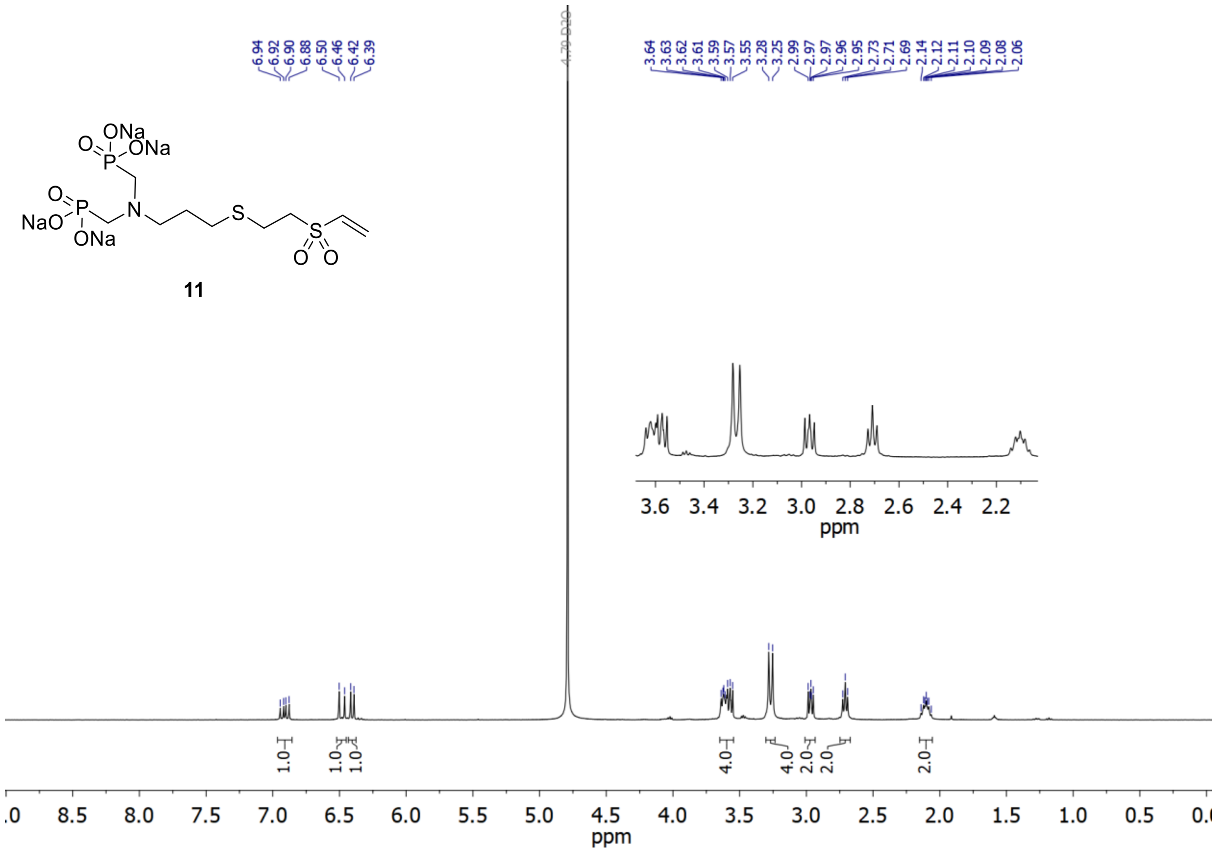

Figure S19. ^{1}H NMR (400 MHz, D_2O) spectrum of **11**.

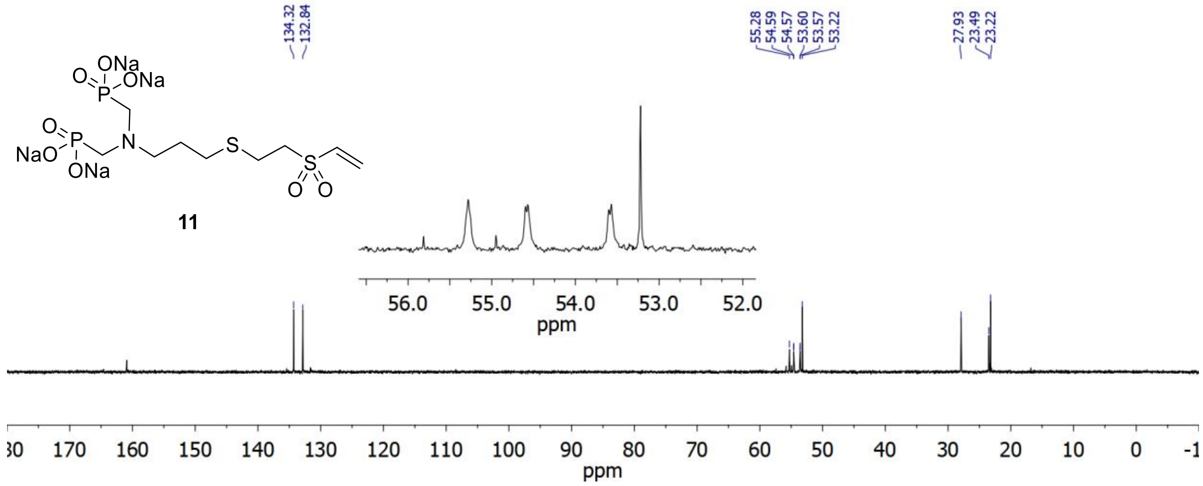

Figure S20. ^{13}C NMR (126 MHz, D_2O) spectrum of **11**.

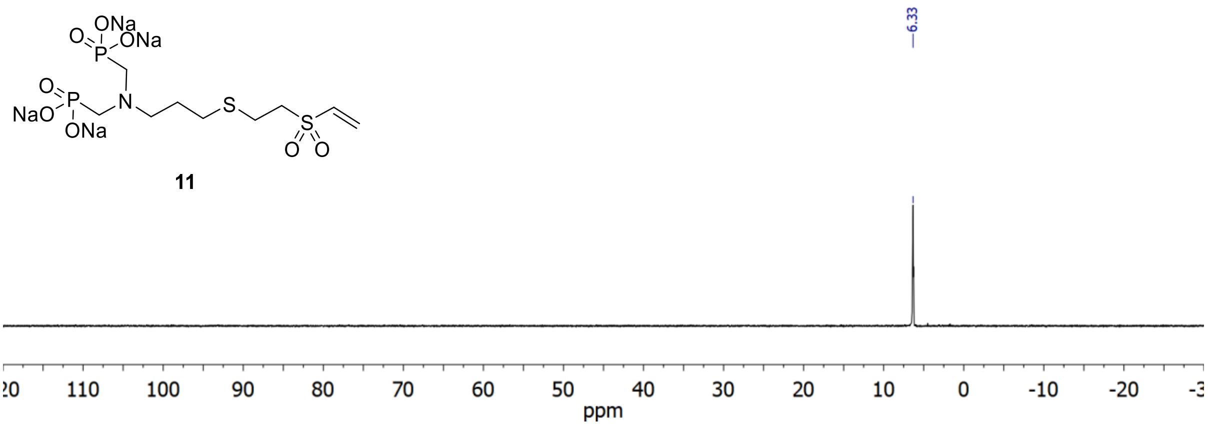

Figure S21. ^{31}P NMR (202 MHz, D_2O) spectrum of **11**.

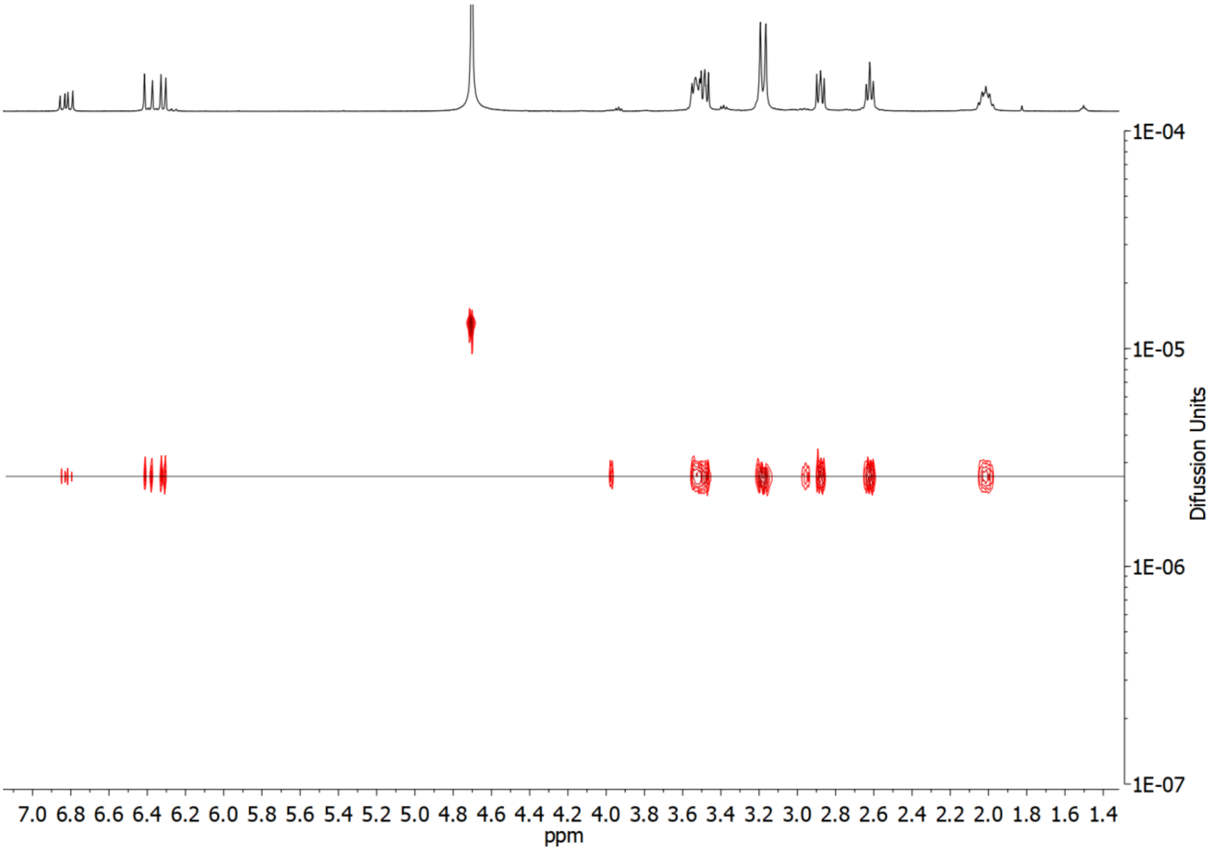

Figure S22. DOSY NMR (500 MHz, D_2O) spectrum of **11**.

Compound 13:

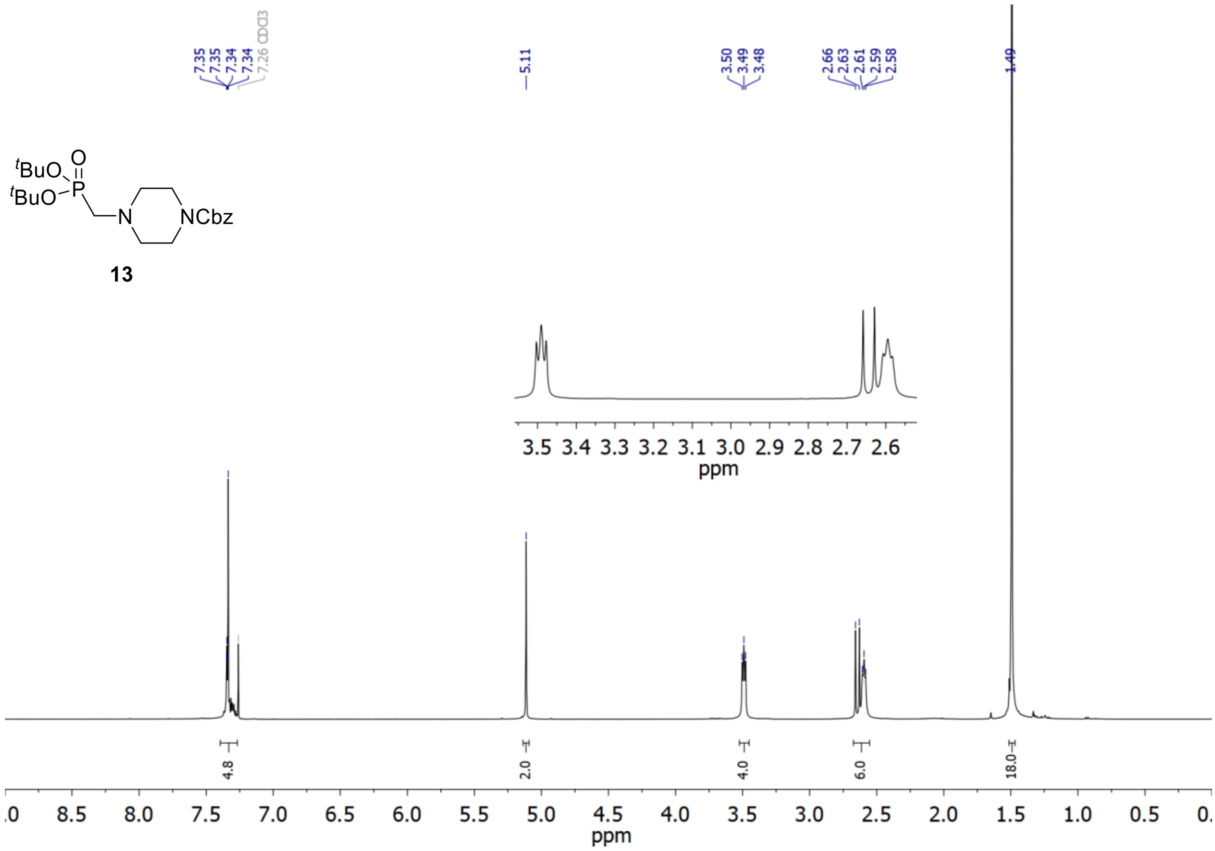

Figure S23. ^{1}H NMR (400 MHz, $CDCl_3$) spectrum of **13**.

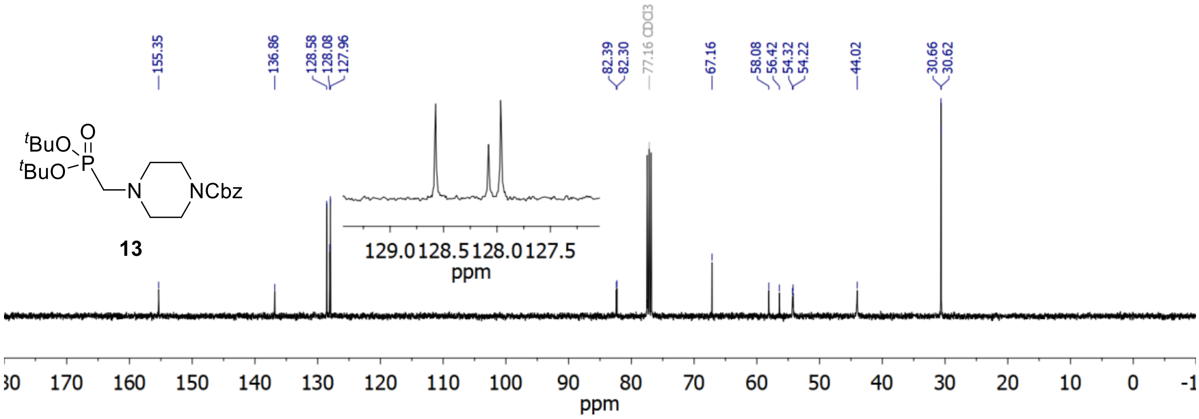

Figure S24. ^{13}C NMR (101 MHz, $CDCl_3$) spectrum of **13**.

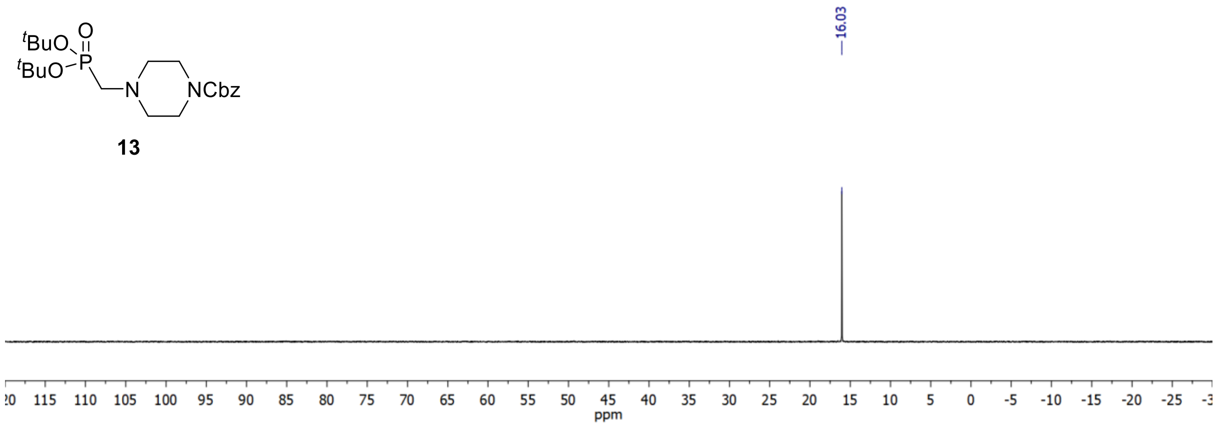

Figure S25. ^{31}P NMR (162 MHz, $CDCl_3$) spectrum of **13**.

Compound 14:

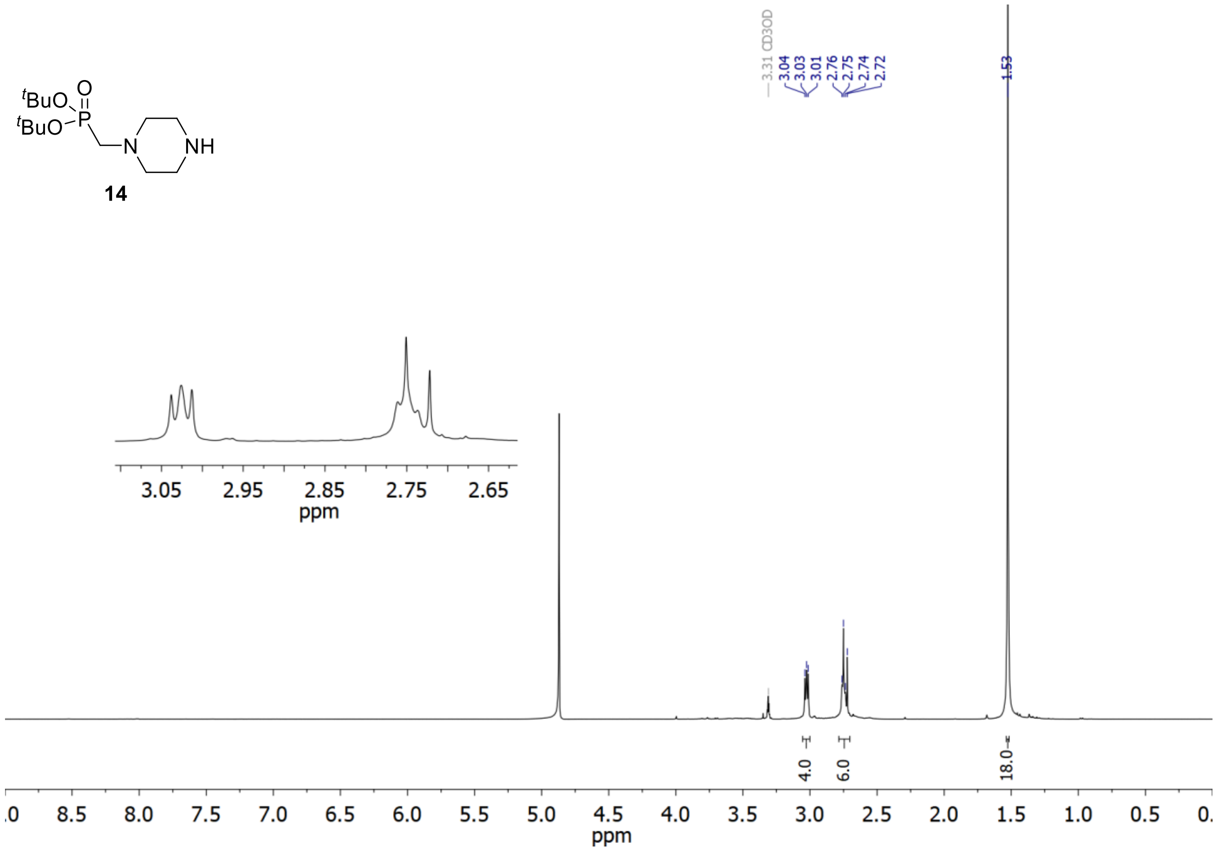

Figure S26. ^{1}H NMR (400 MHz, CD_3OD) spectrum of **14**.

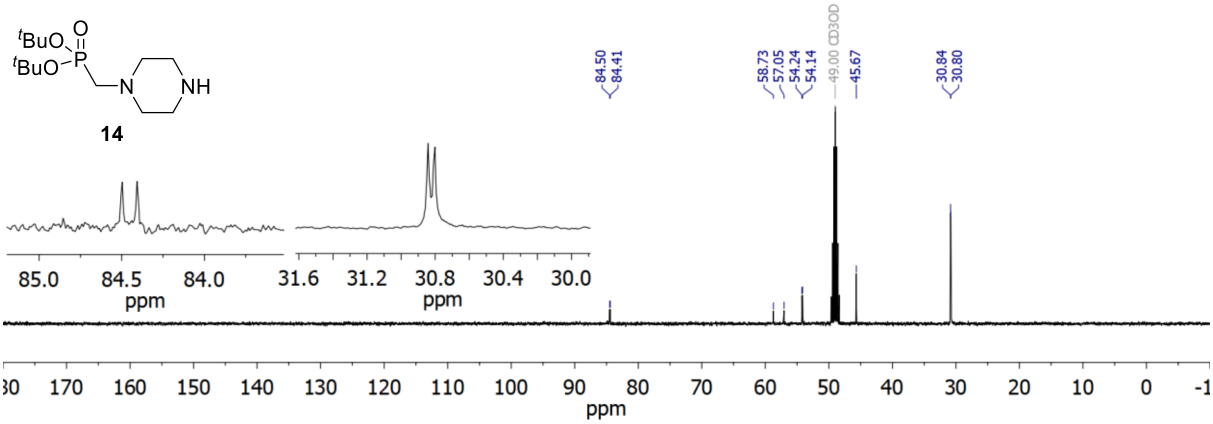

Figure S27. ^{13}C NMR (101 MHz, CD_3OD) spectrum of **14**.

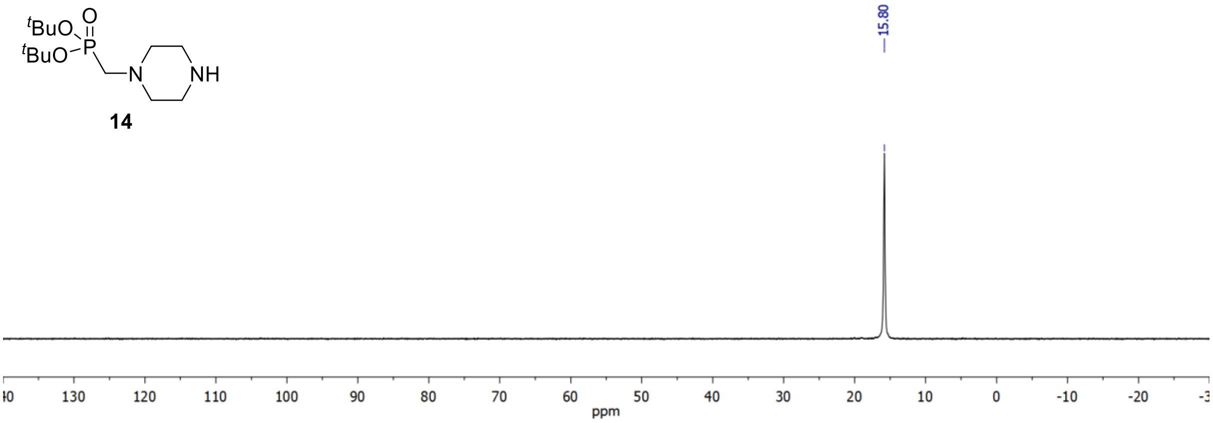

Figure S28. ^{31}P NMR (162 MHz, CD_3OD) spectrum of **14**.

Compound 16:

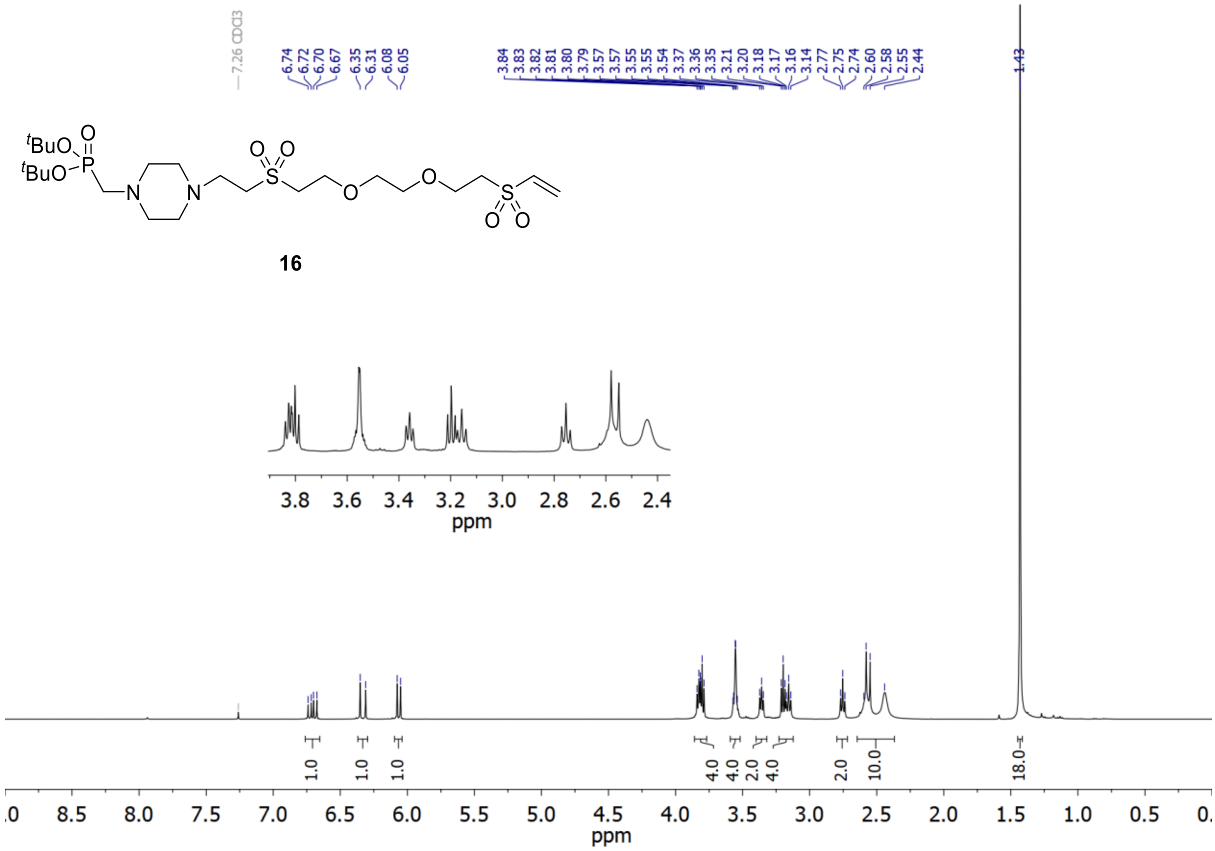

Figure S29. ^{1}H NMR (400 MHz, $CDCl_3$) spectrum of **16**.

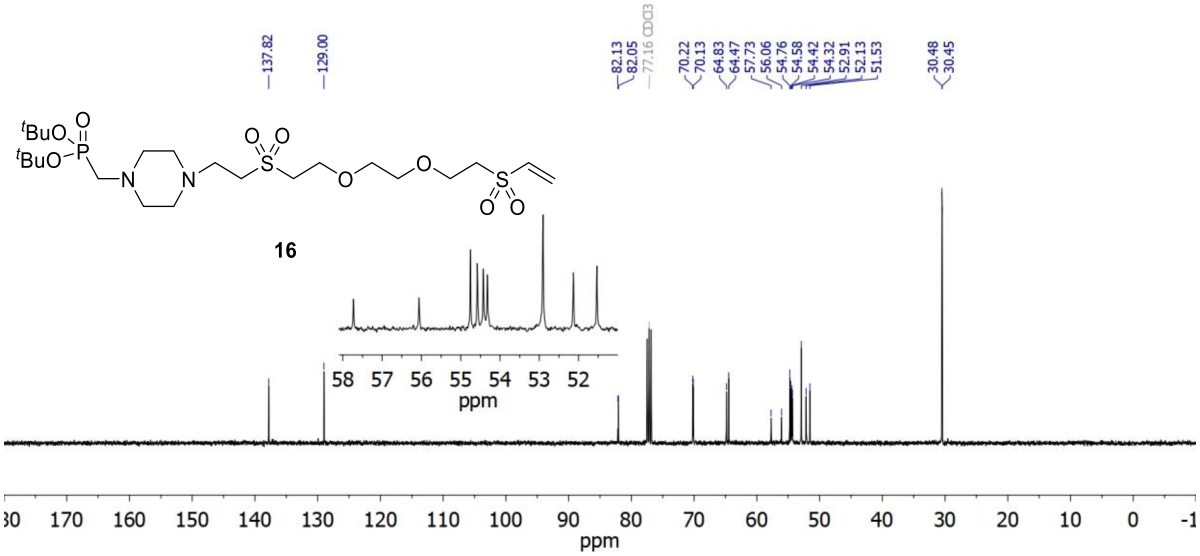

Figure 30. ^{13}C NMR (101 MHz, $CDCl_3$) spectrum of **16**.

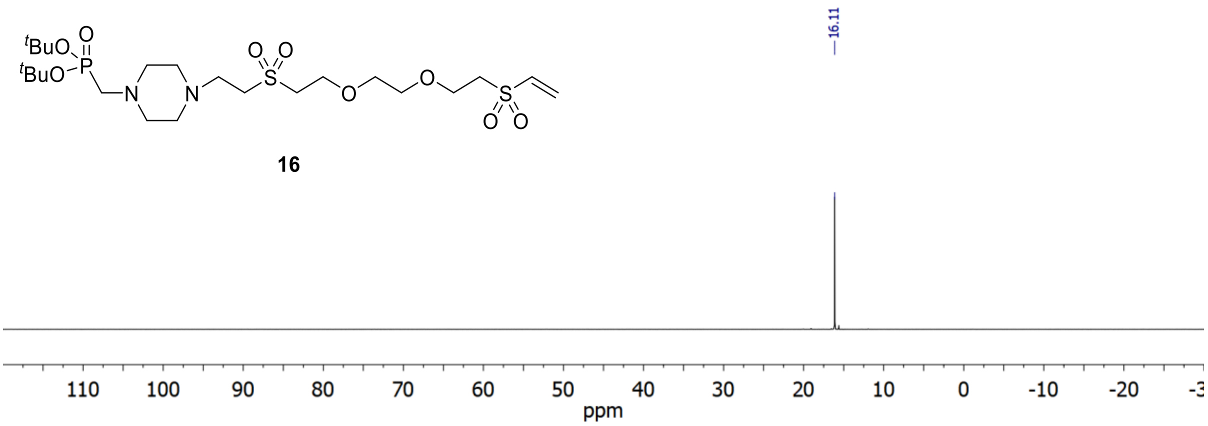

Figure S31. ^{31}P NMR (202 MHz, $CDCl_3$) spectrum of **16**.

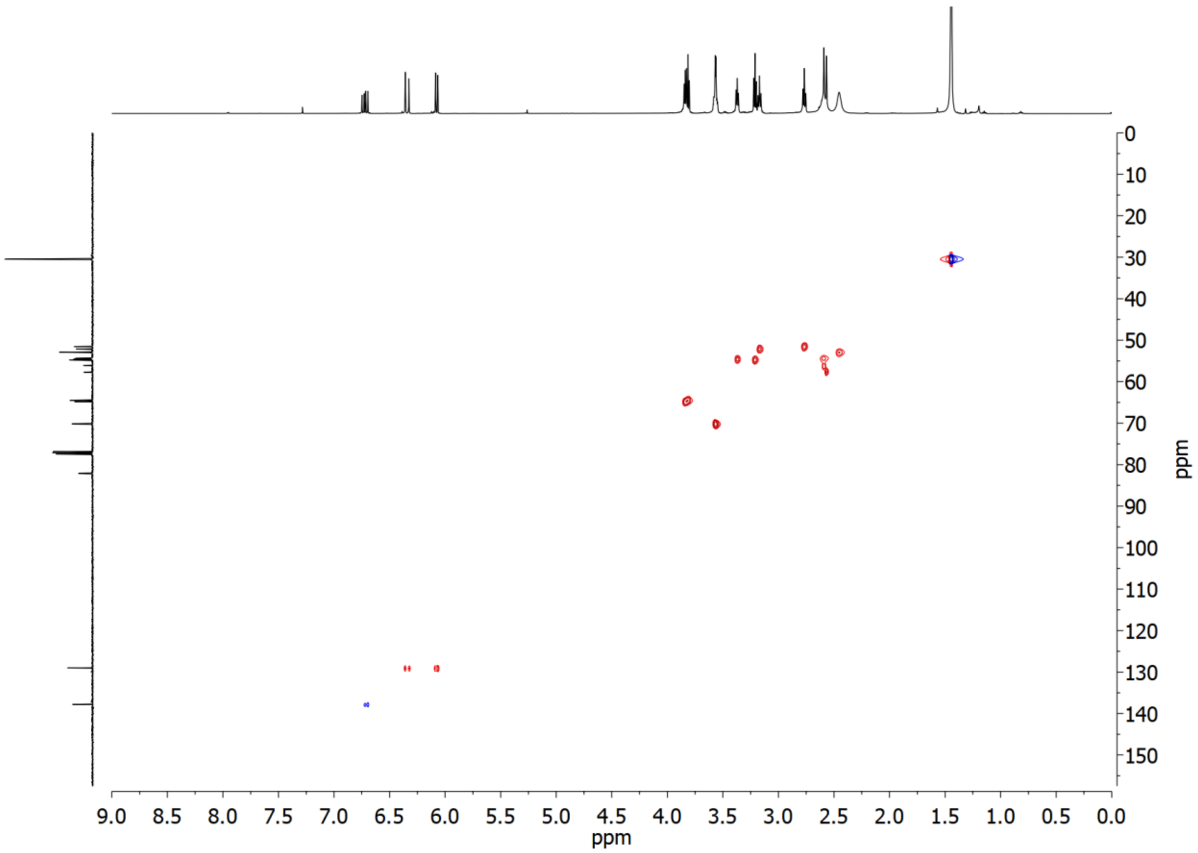

Figure S32. HSQC NMR (500 MHz and 126 MHz, $CDCl_3$) spectrum of **16**.

Compound 17:

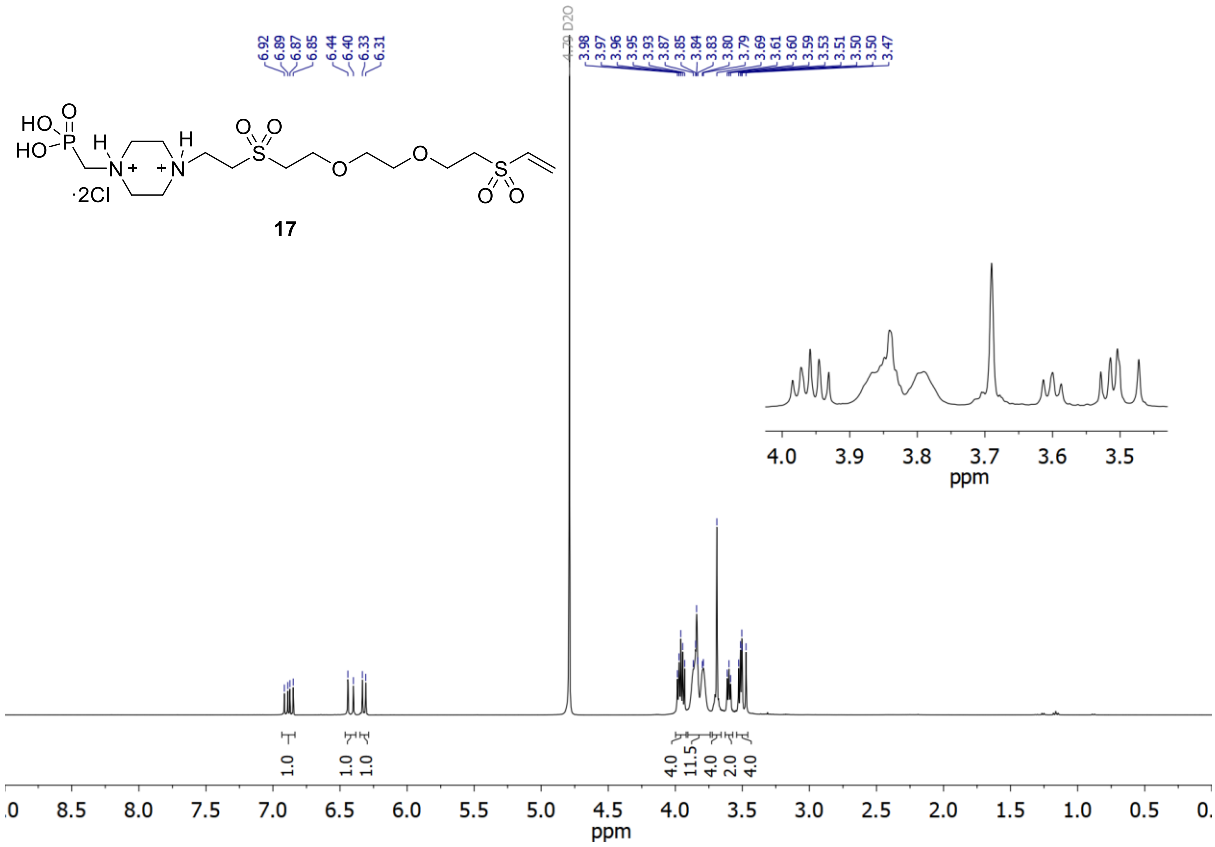

Figure S33. ^{1}H NMR (400 MHz, D_2O) spectrum of **17**.

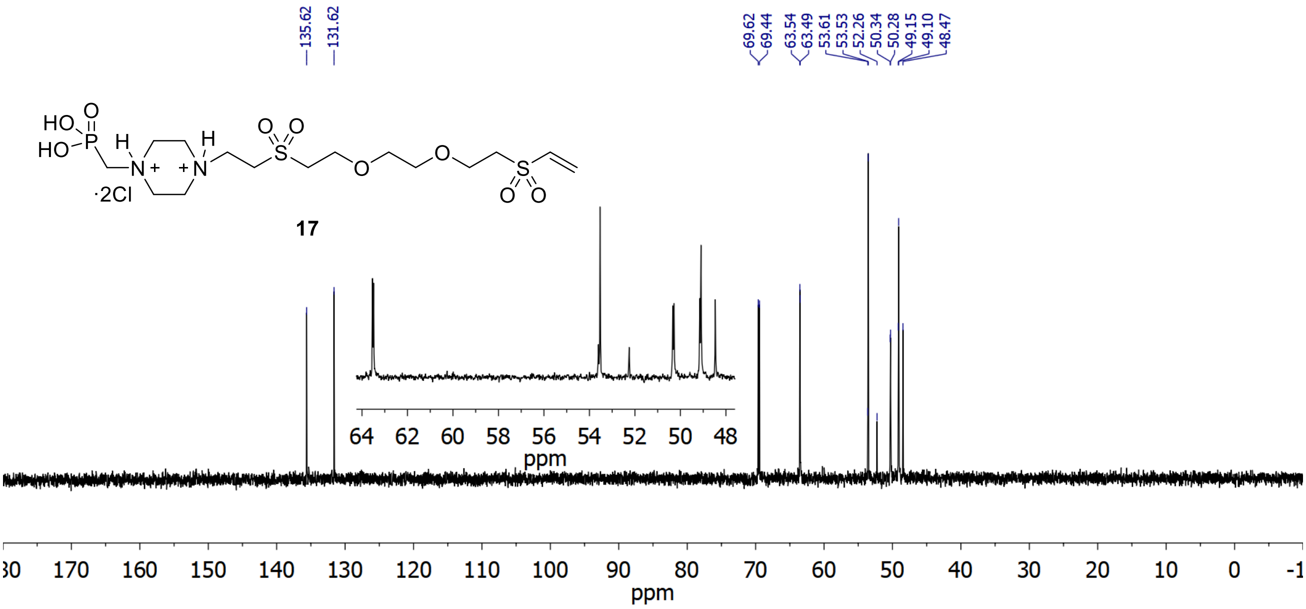

Figure S34. ^{13}C NMR (101 MHz, D_2O) spectrum of **17**.

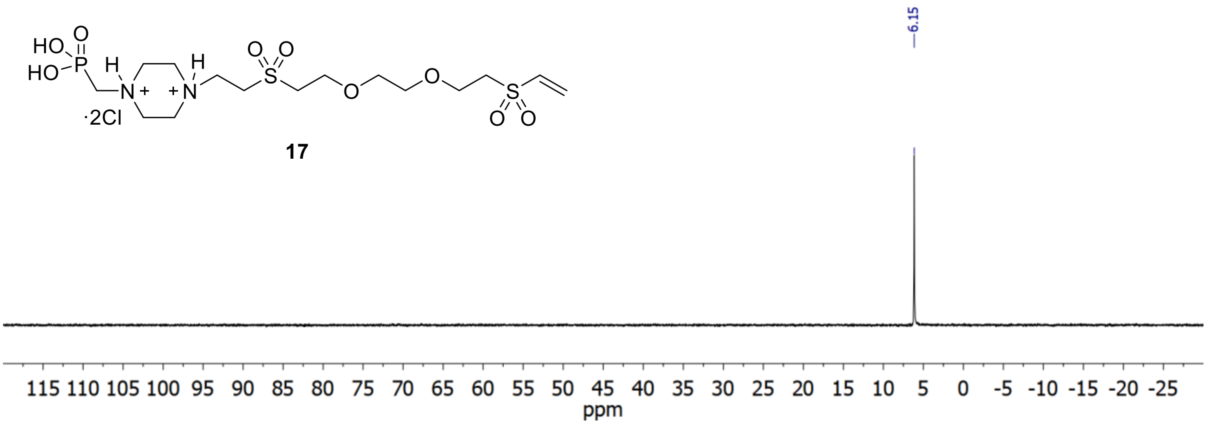

Figure S35. ^{31}P NMR (162 MHz, D_2O) spectrum of **17**.

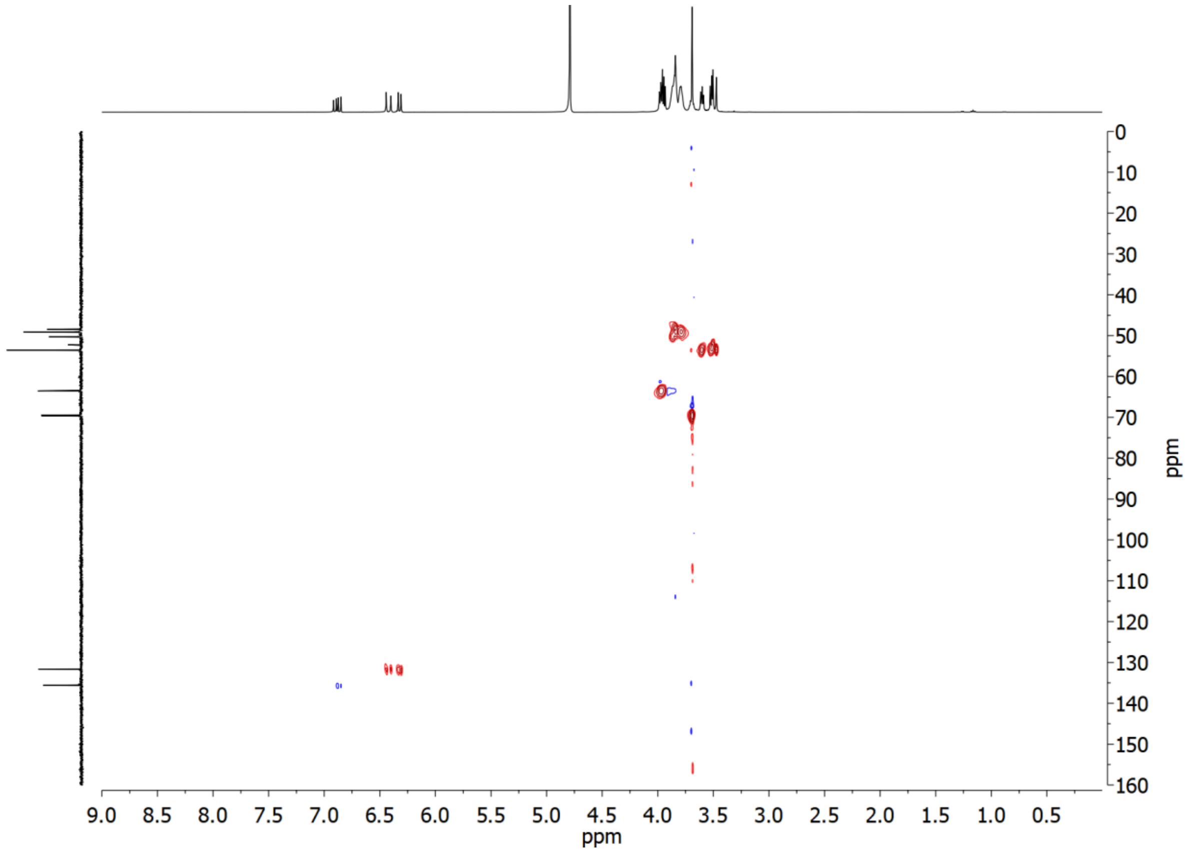

Figure S36. HSQC NMR (400 MHz and 101 MHz, D_2O) spectrum of **17**.

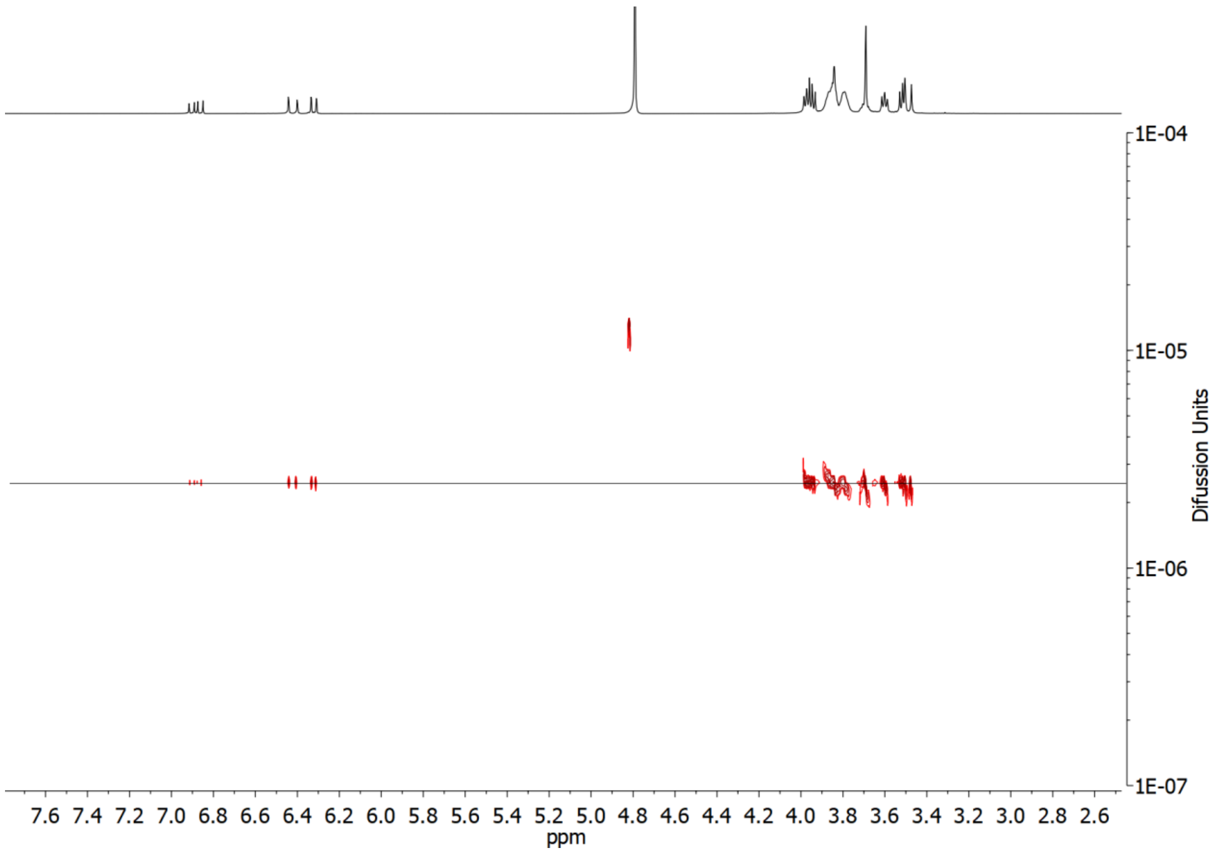

Figure S37. DOSY NMR (500 MHz, D_2O) spectrum of **17**.

Compound 20a:

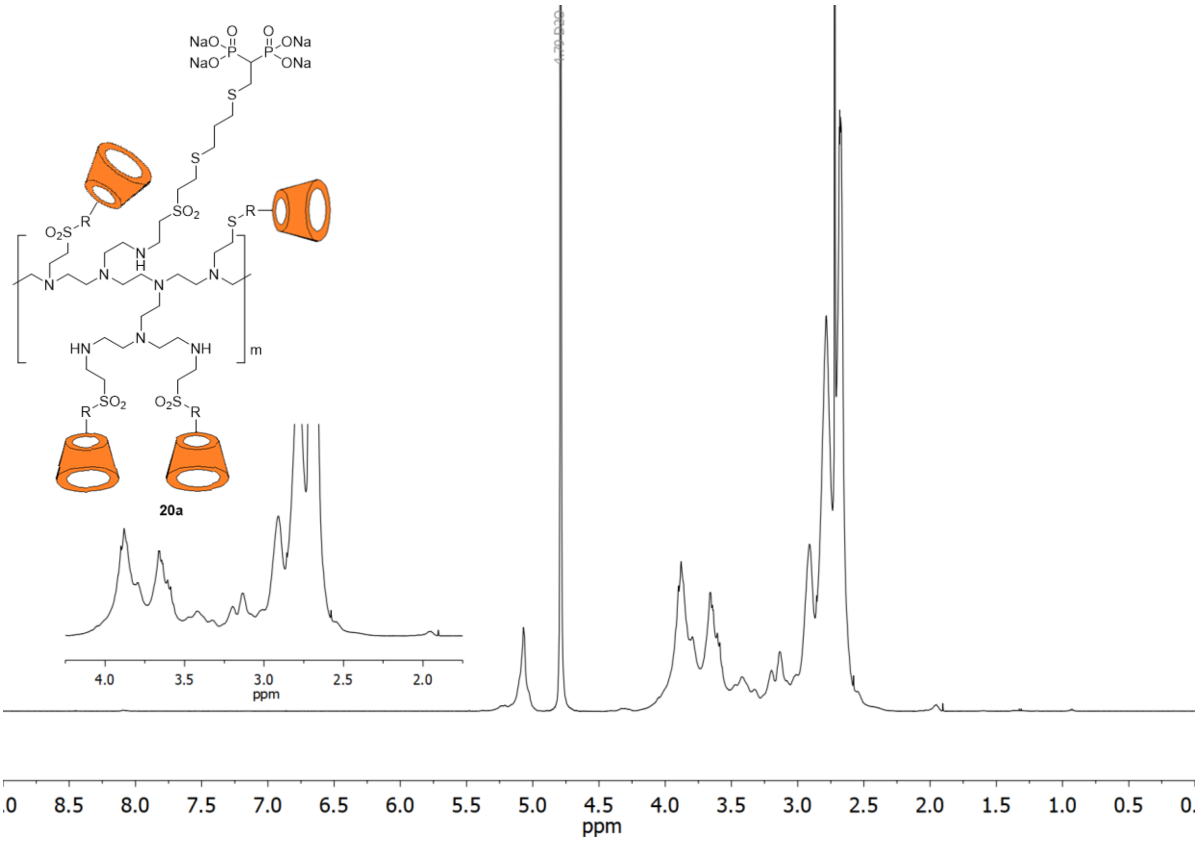

Figure S38. ^{1}H NMR (500 MHz, D_2O/DMSO-d_6) spectrum of **20a**.

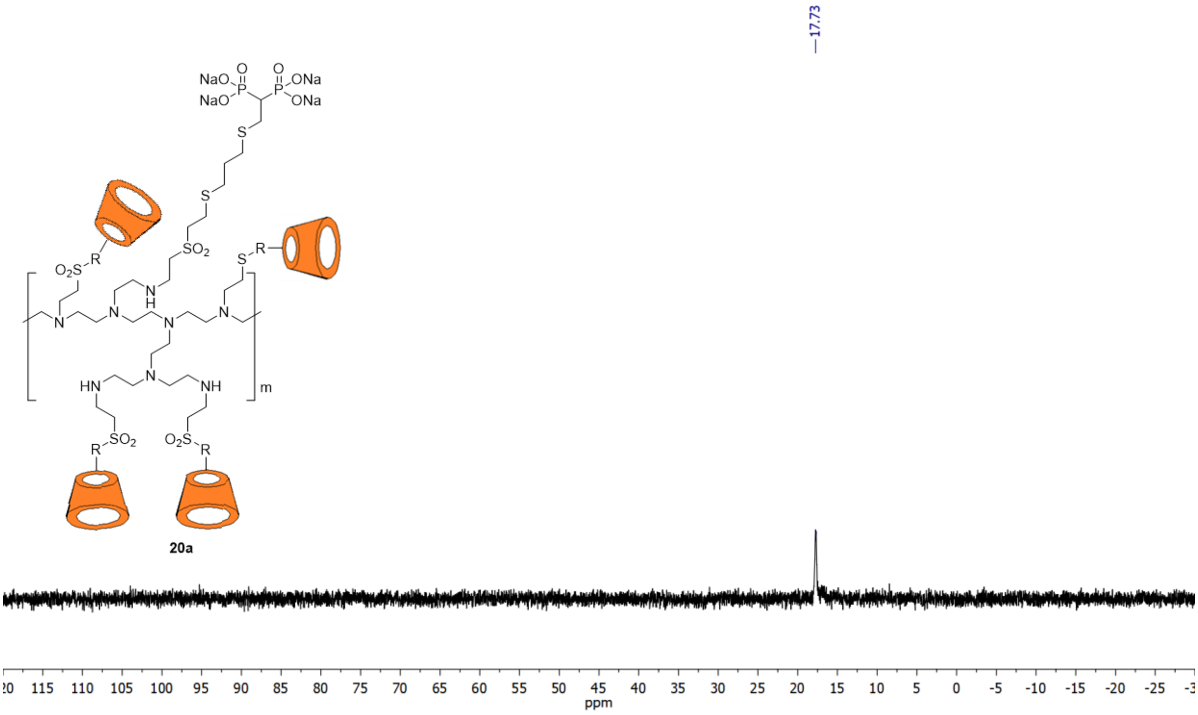

Figure S39. ^{31}P NMR (202 MHz, D_2O/DMSO-d_6) spectrum of **20a**.

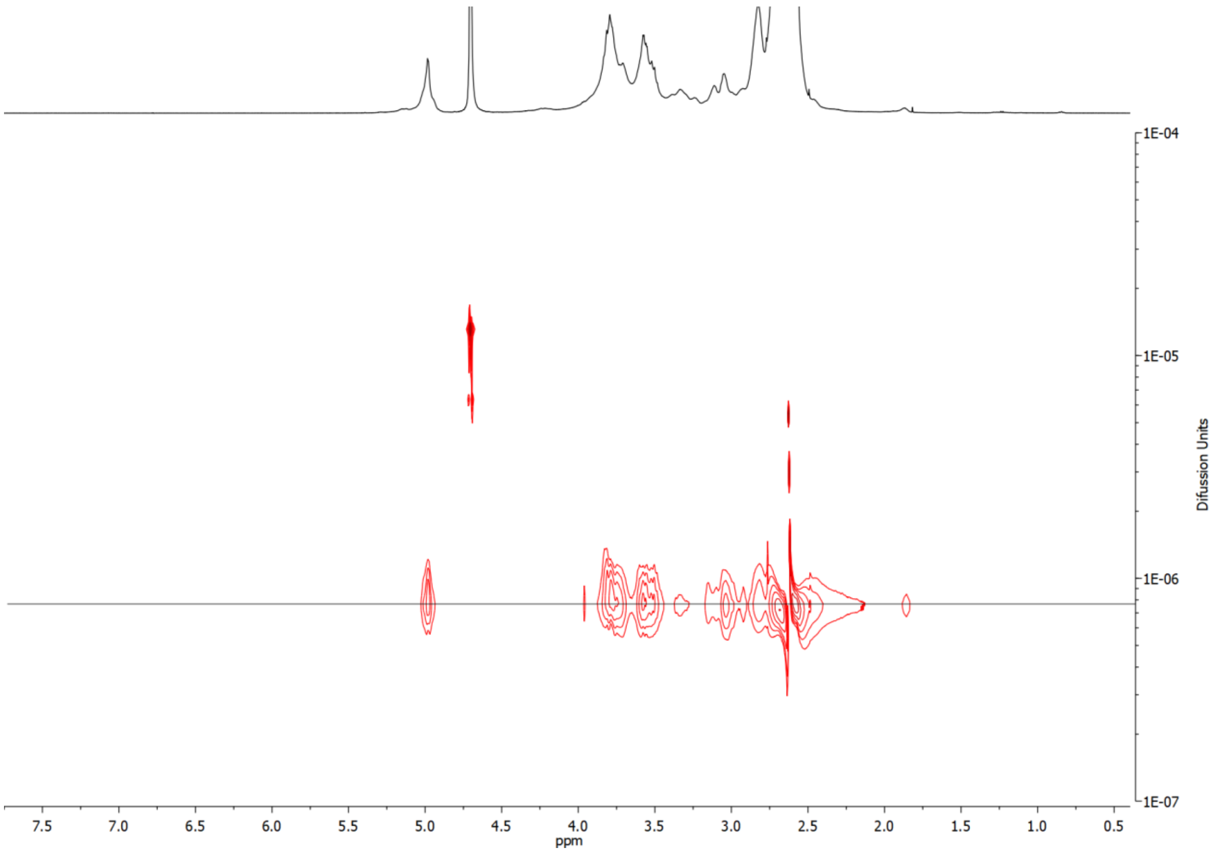

Figure S40. DOSY NMR (500 MHz, D_2O/DMSO-d_6) spectrum of **20a**.

Compound 20b:

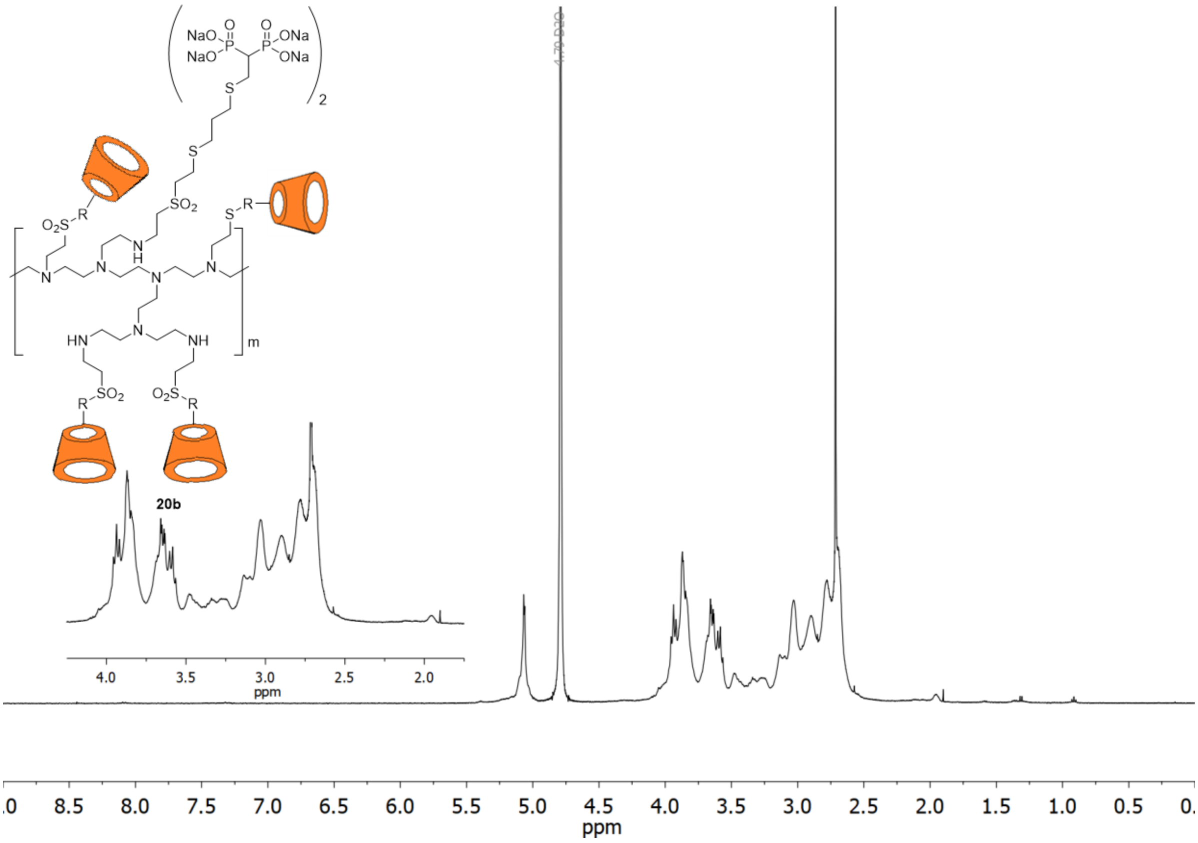

Figure S41. ^{1}H NMR (500 MHz, D_2O/DMSO-d_6) spectrum of **20b**.

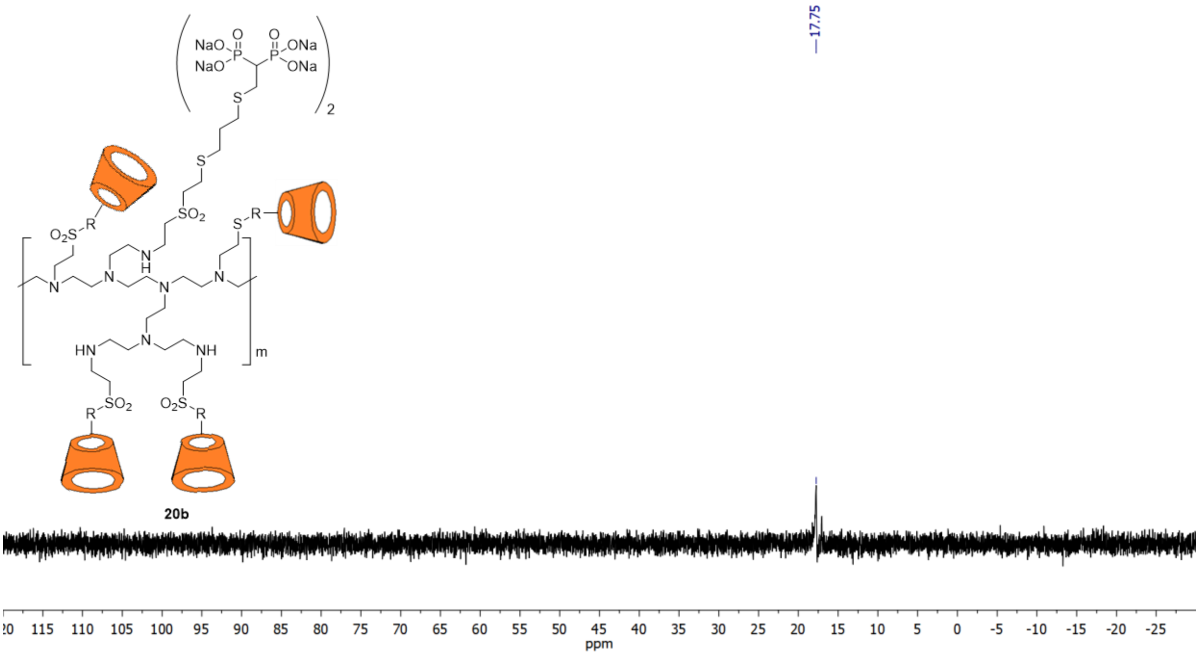

Figure S42. ^{31}P NMR (162 MHz, D_2O/DMSO-d_6) spectrum of **20b**.

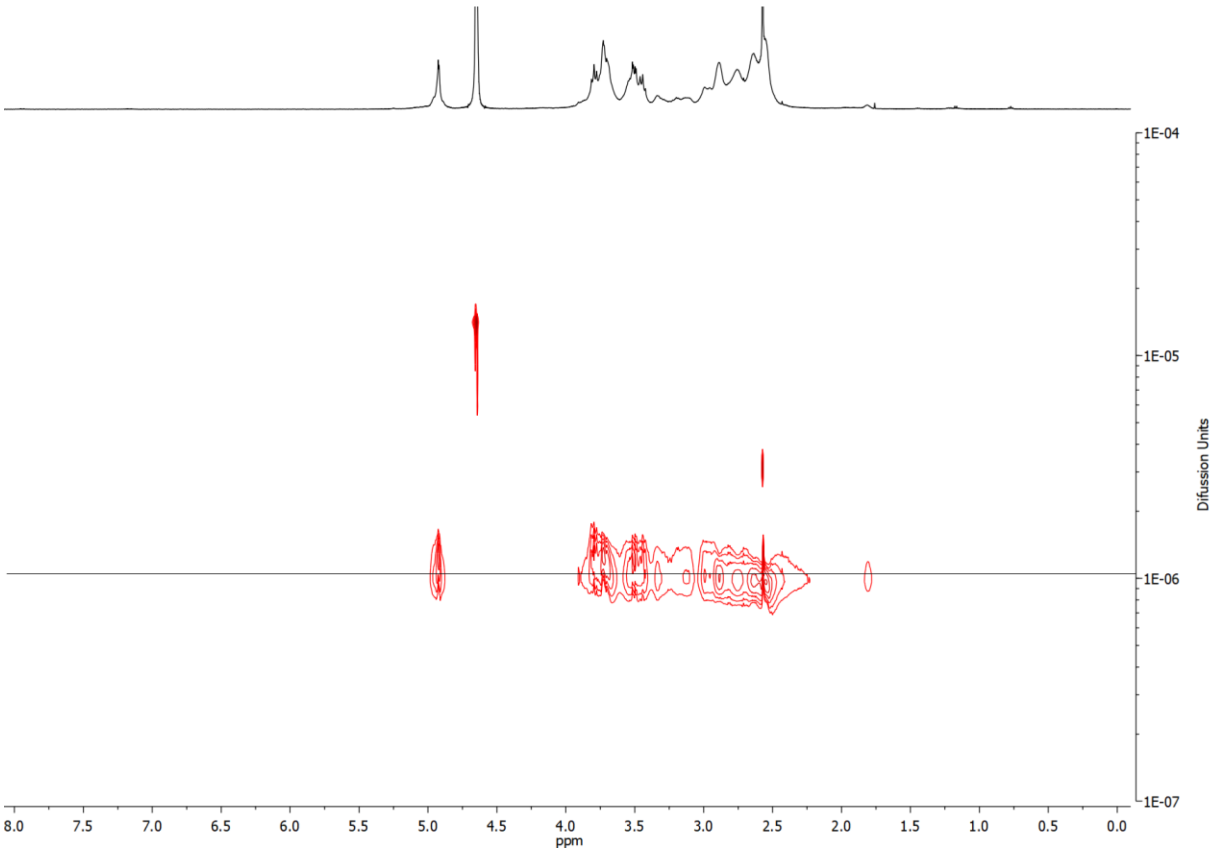

Figure S43. DOSY NMR (500 MHz, D_2O/DMSO-d_6) spectrum of **20b**.

Compound 21a:

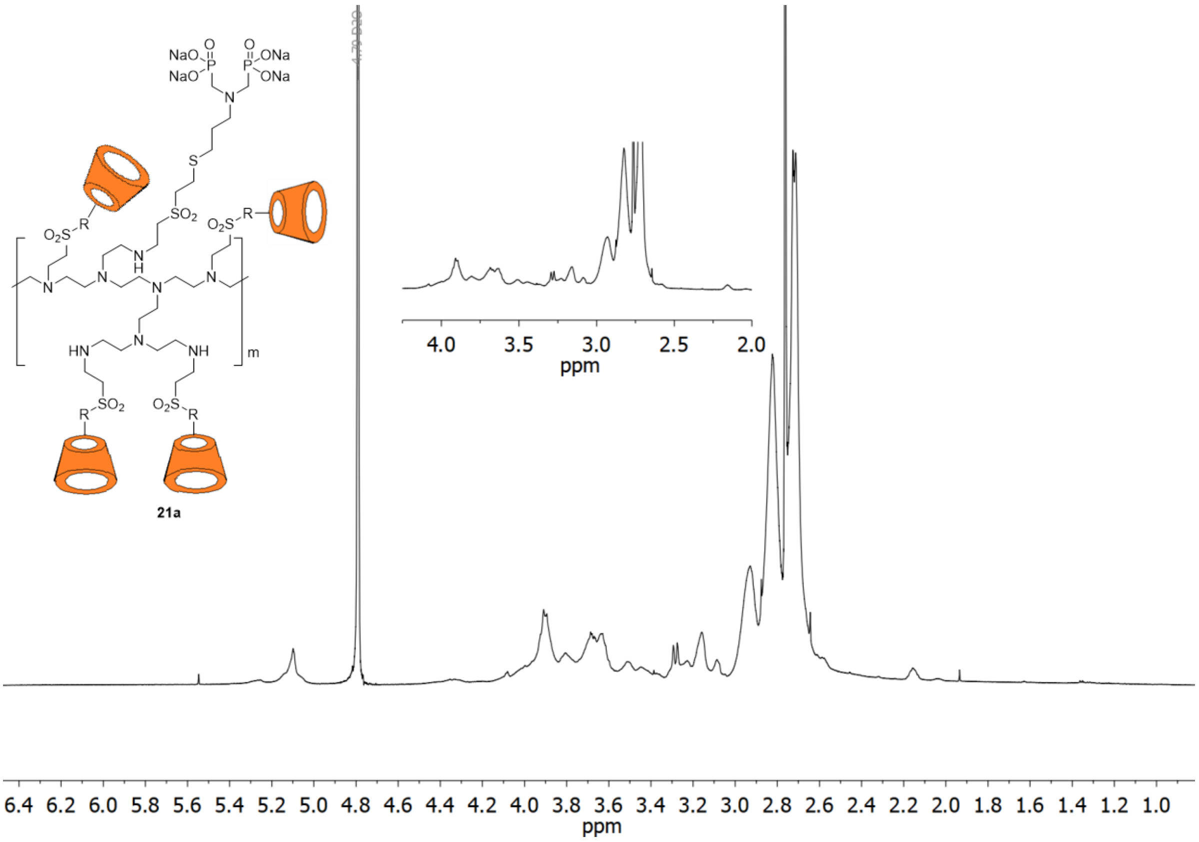

Figure S44. Partial ^{1}H NMR (600 MHz, D_2O/DMSO-d_6) spectrum of **21a**.

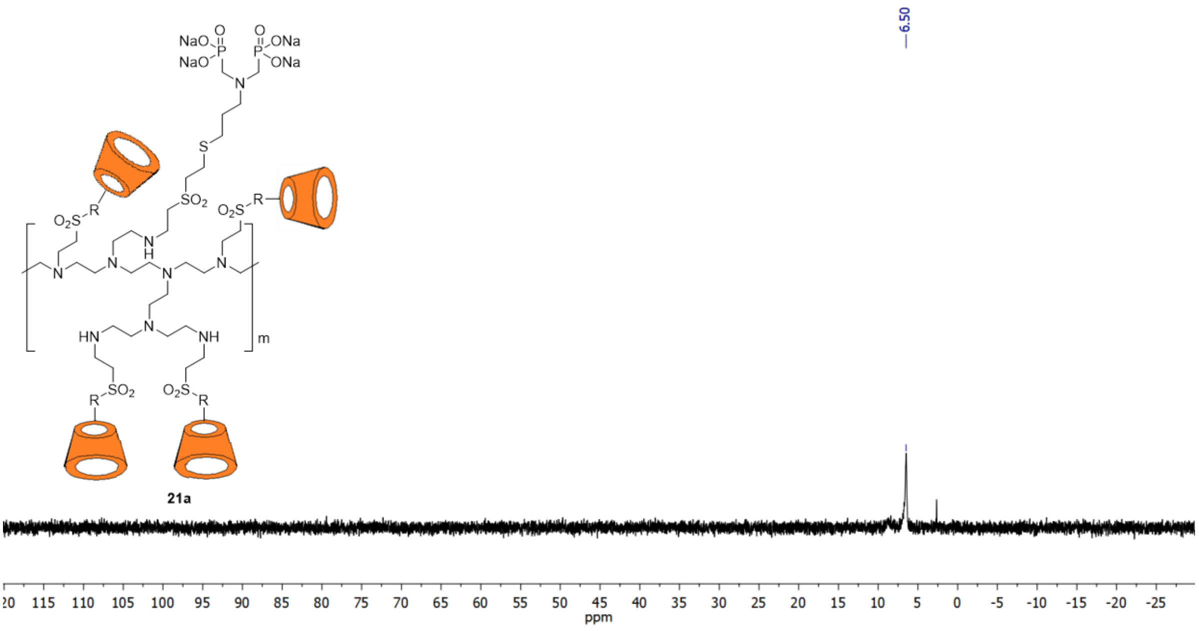

Figure S45. ^{31}P NMR (202 MHz, D_2O/DMSO-d_6) spectrum of **21a**.

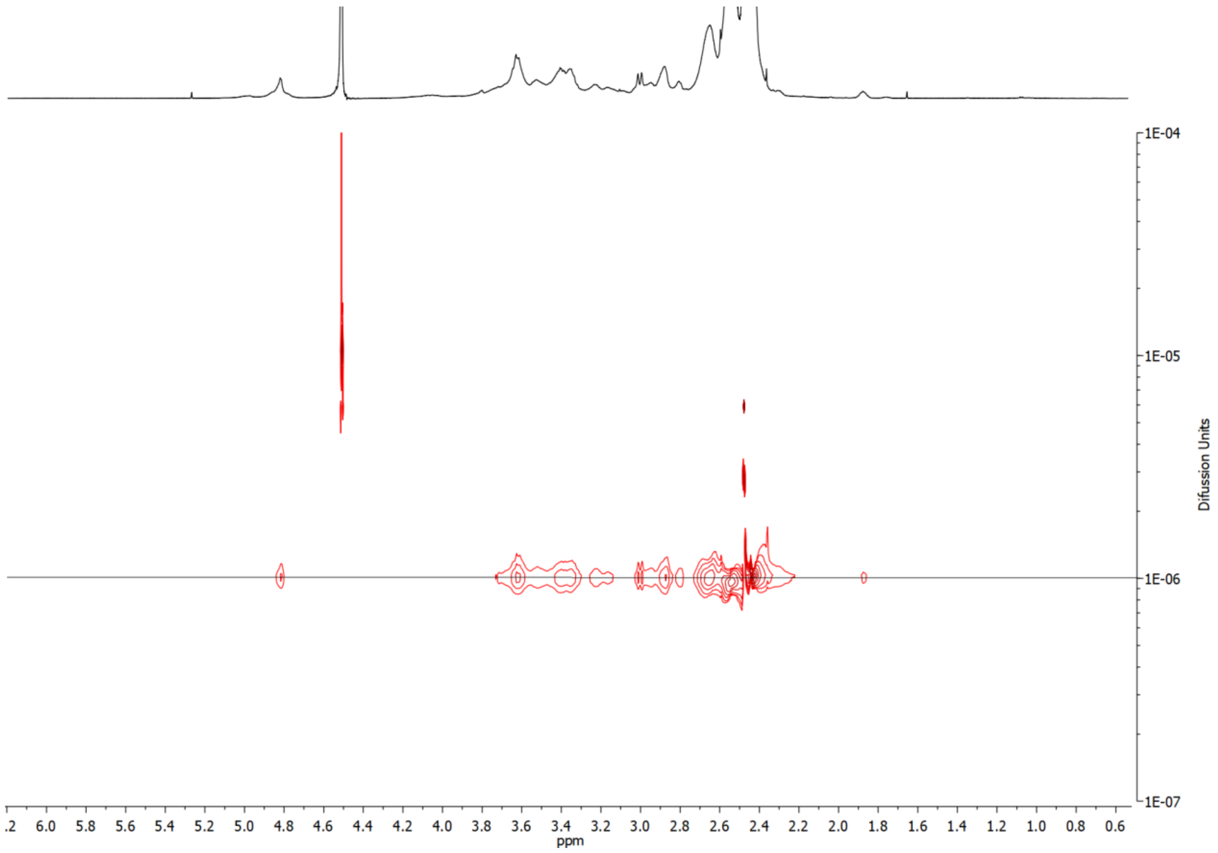

Figure S45. DOSY NMR (600 MHz, D_2O/DMSO-d_6) spectrum of **21a**.

Compound 21b:

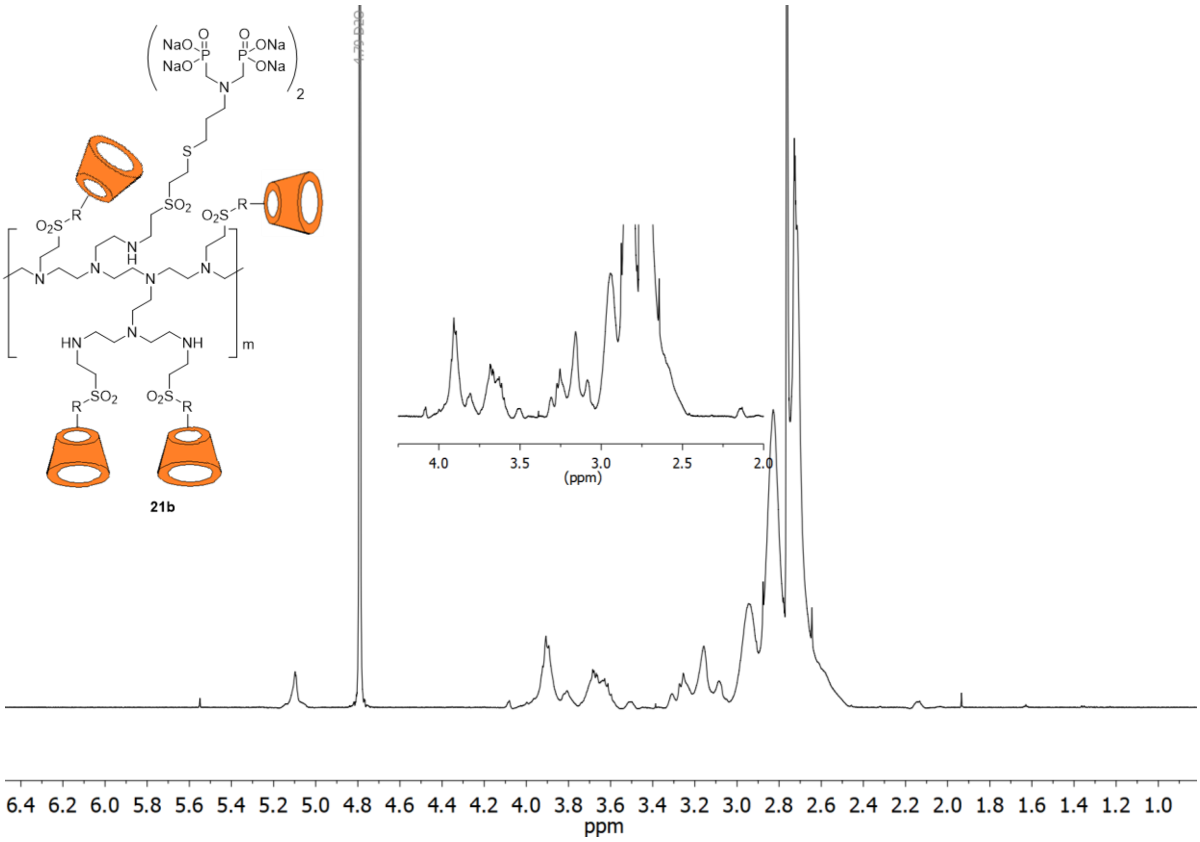

Figure S47. Partial ^{1}H NMR (600 MHz, D_2O/DMSO-d_6) spectrum of **21b**.

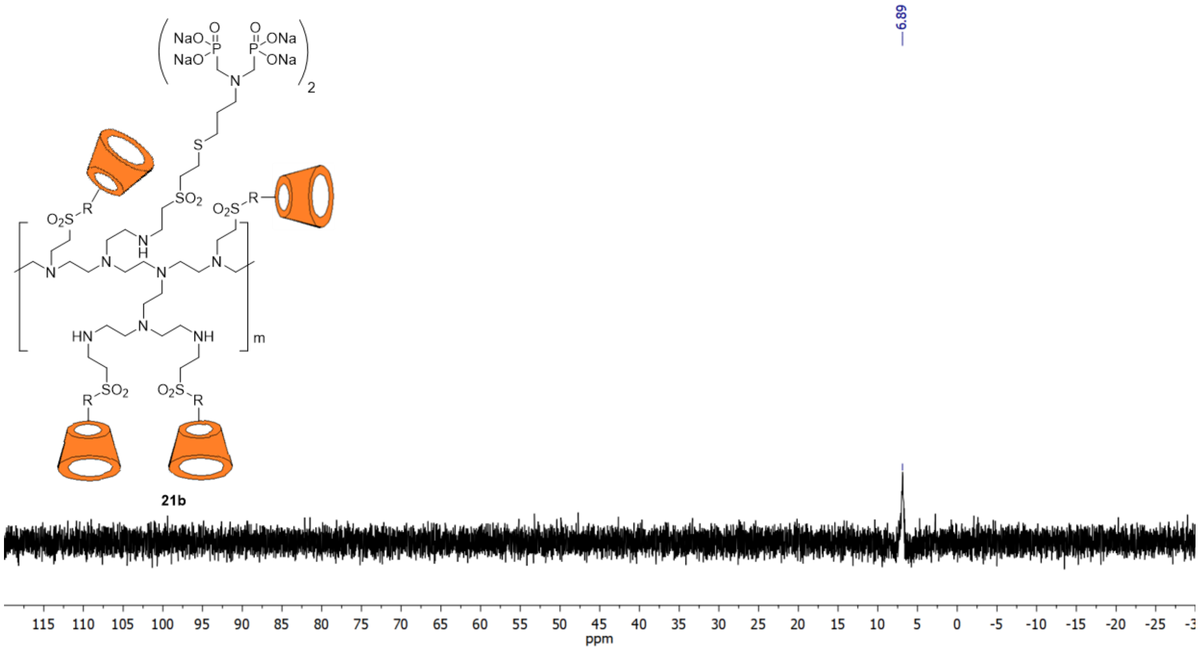

Figure S48. ^{31}P NMR (202 MHz, D_2O/DMSO-d_6) spectrum of **21b**.

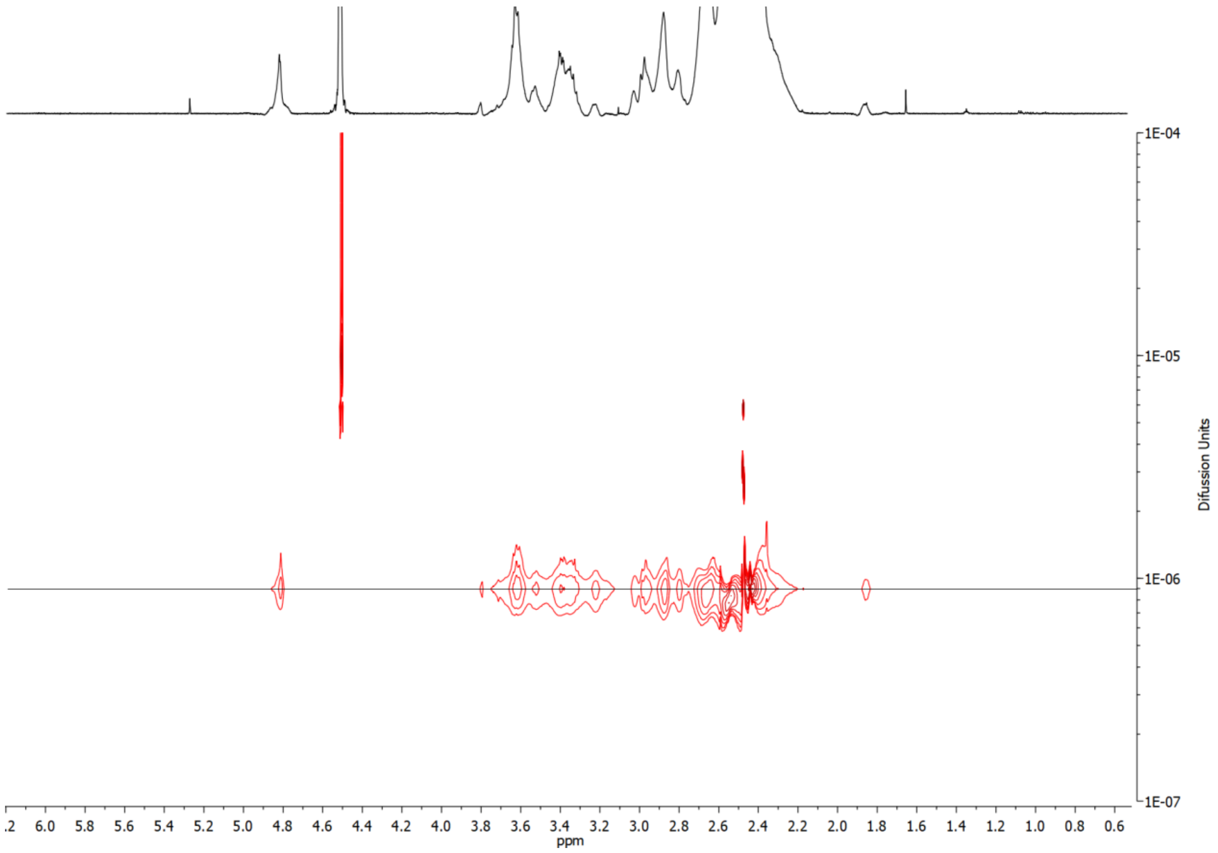

Figure S49. DOSY NMR (600 MHz, D_2O/DMSO-d_6) spectrum of **21b**.

Compound 22a:

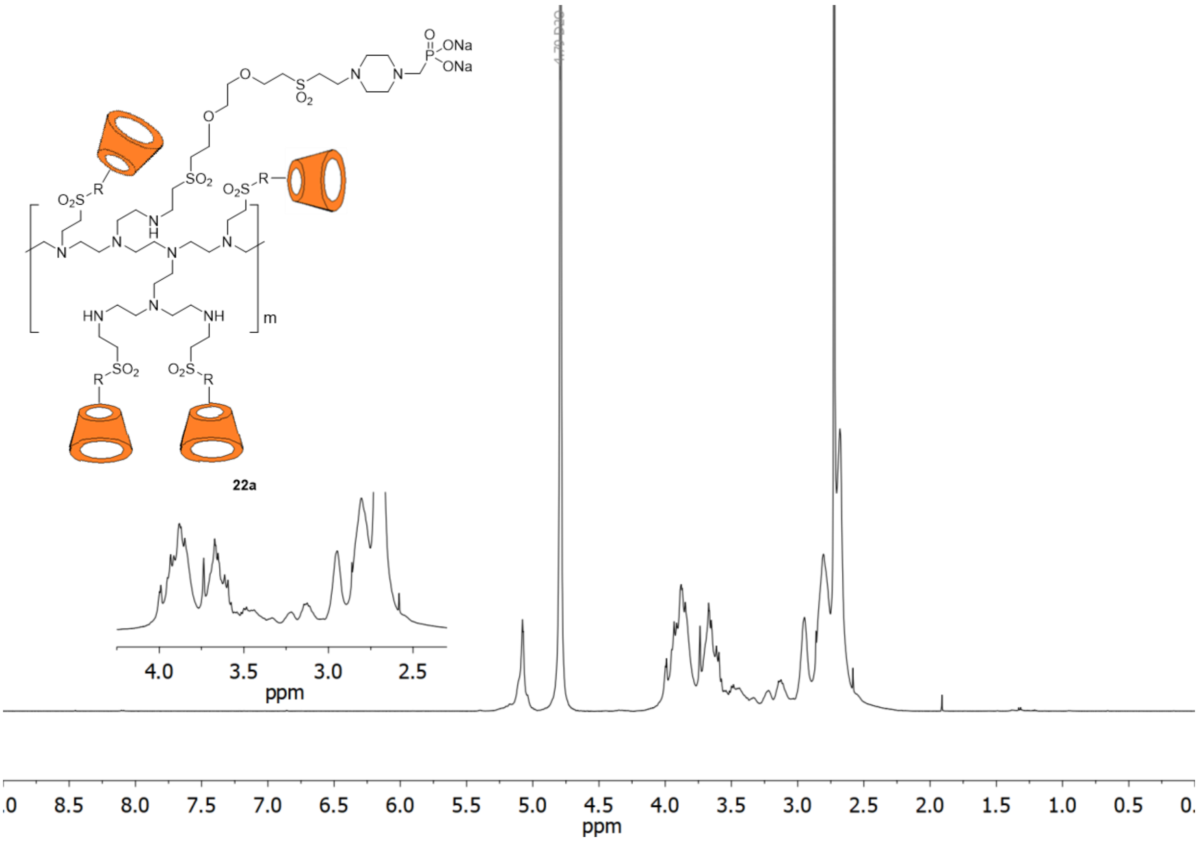

Figure S50. ^{1}H NMR (500 MHz, D_2O/DMSO-d_6) spectrum of **22a**.

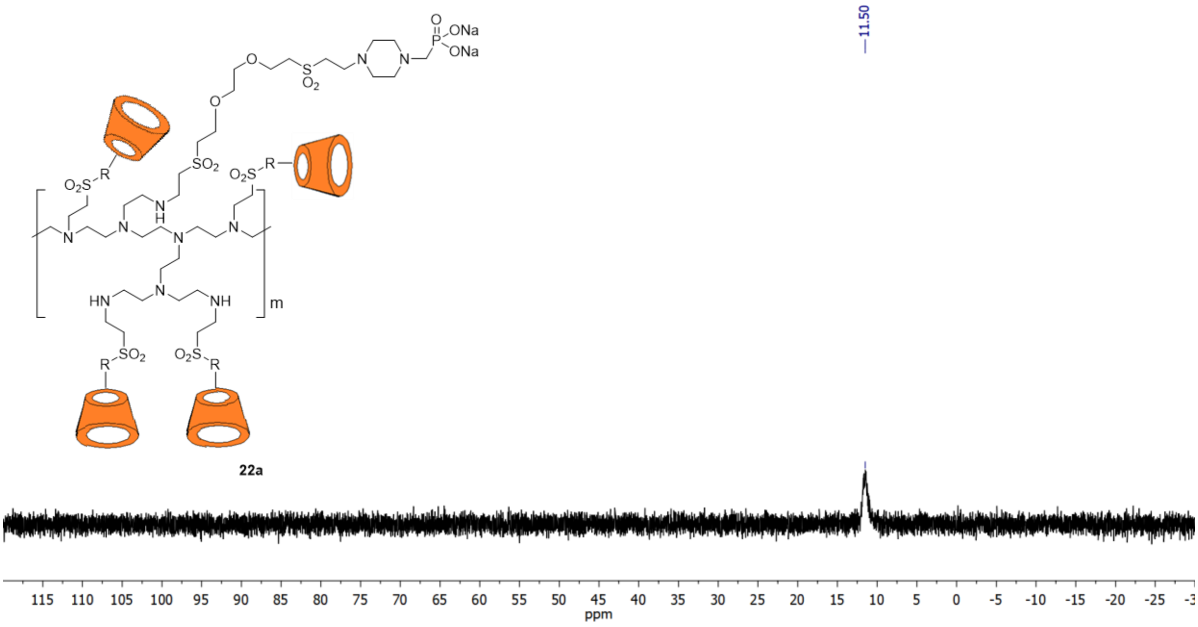

Figure S51. ^{31}P NMR (202 MHz, D_2O/DMSO-d_6) spectrum of **22a**.

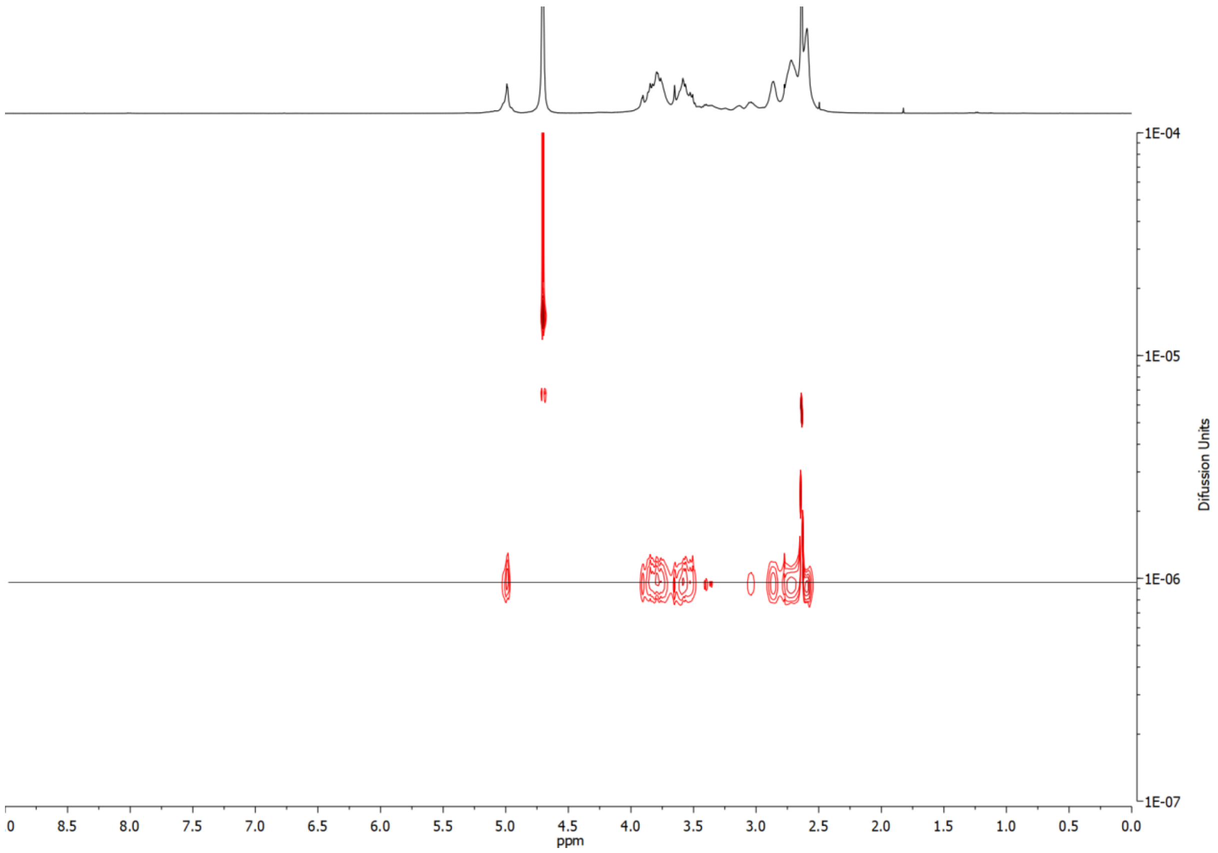

Figure S52. DOSY NMR (500 MHz, D_2O/DMSO-d_6) spectrum of **22a**.

Compound 22b:

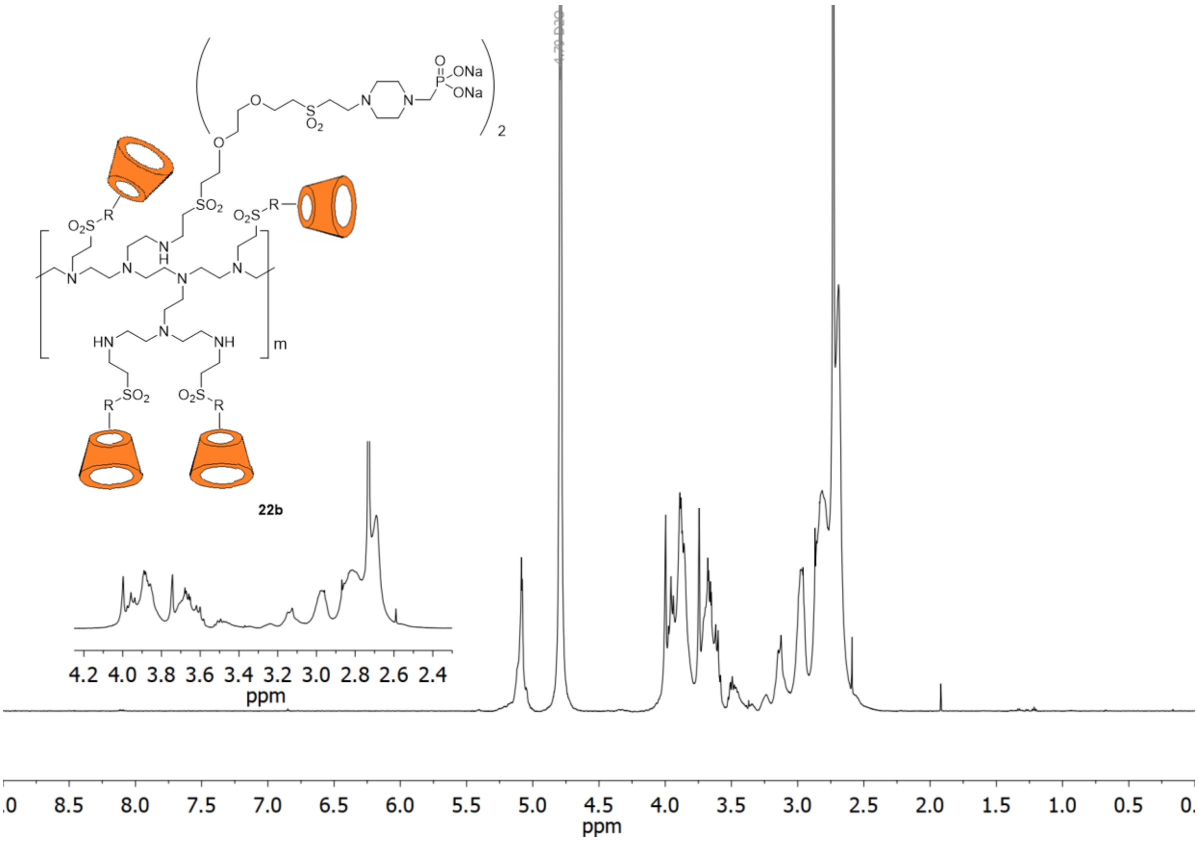

Figure S53. ^{1}H NMR (500 MHz, D_2O/DMSO-d_6) spectrum of **22b**.

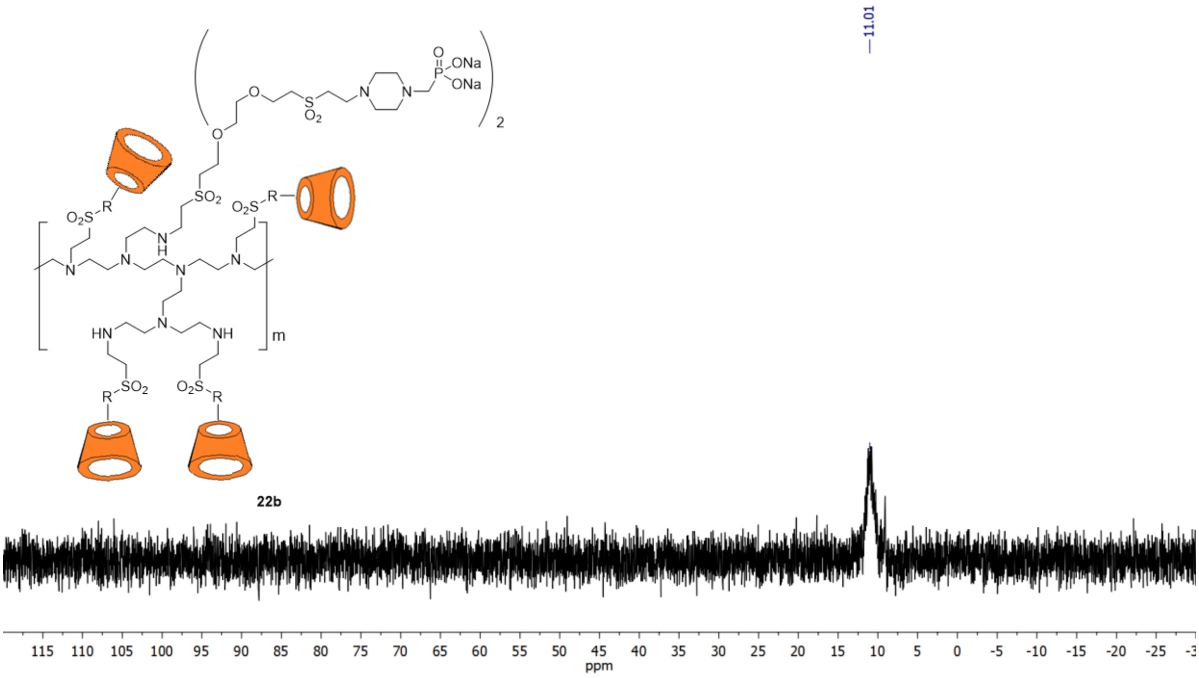

Figure S54. ^{31}P NMR (202 MHz, D_2O/DMSO-d_6) spectrum of **22b**.

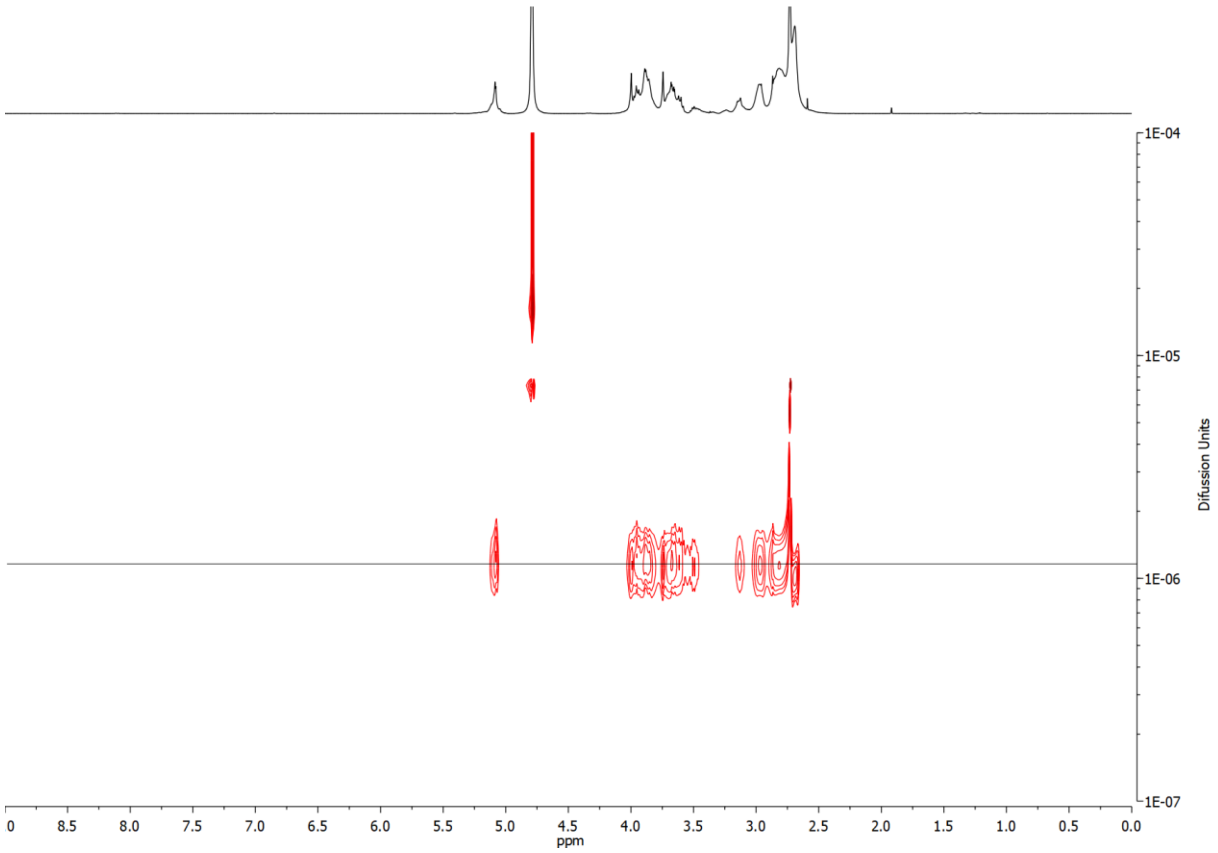

Figure S55. DOSY NMR (500 MHz, D_2O/DMSO-d_6) spectrum of **22b**.

3. HRMS spectra of key compounds

Compound 5

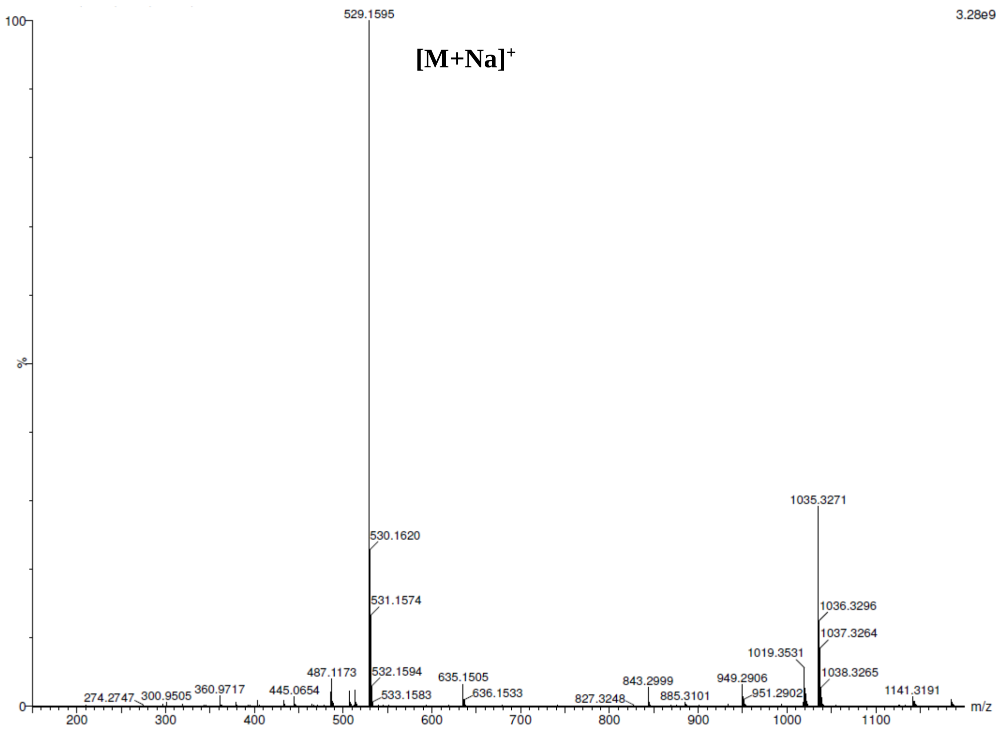

Figure S56. HRMS (ESI$^+$) spectrum of **5**.

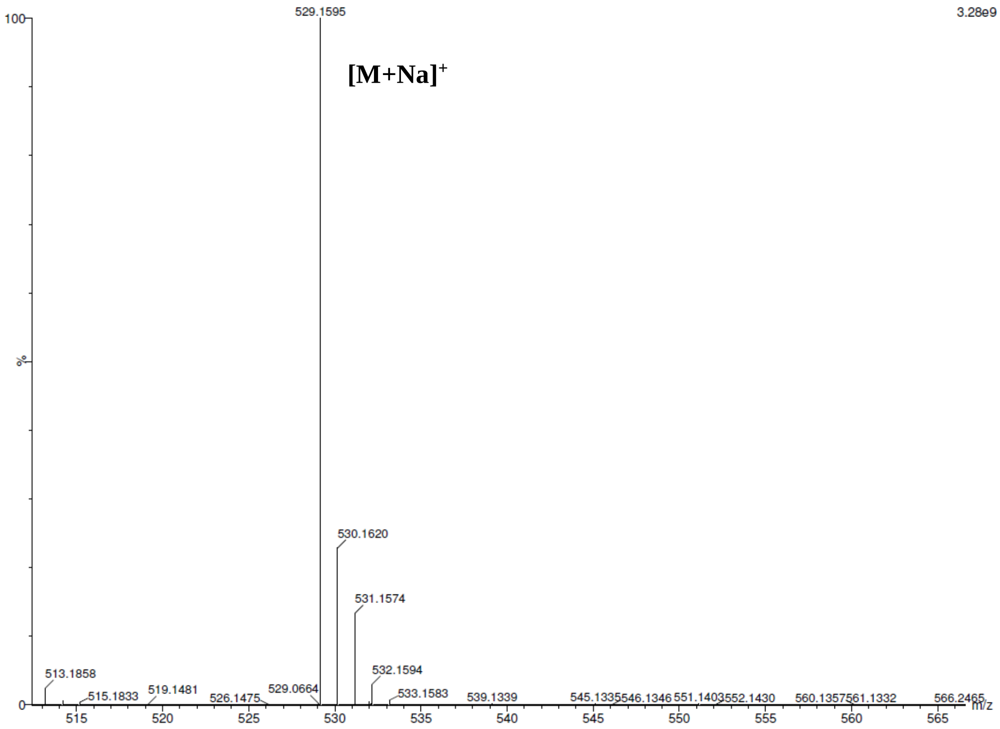

Figure S57. Partial HRMS (ESI$^+$) spectrum of **5**.

Compound 7

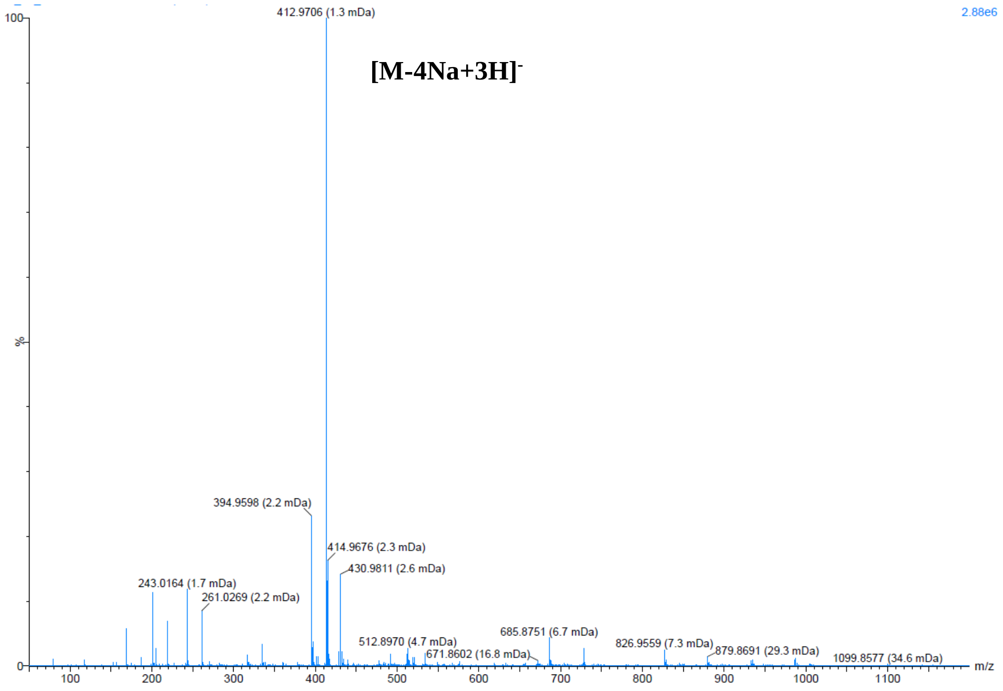

Figure S58. HRMS (ESI$^-$) spectrum of **7** (performed using 0.1% of formic acid).

Compound 9

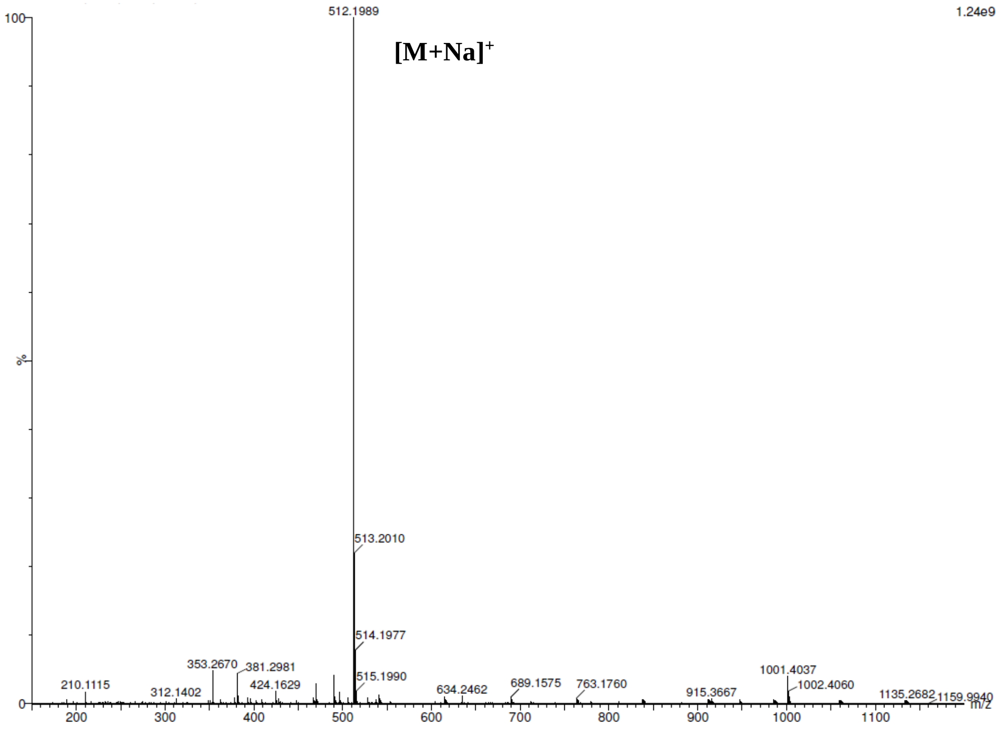

Figure S59. HRMS (ESI$^+$) spectrum of **9**.

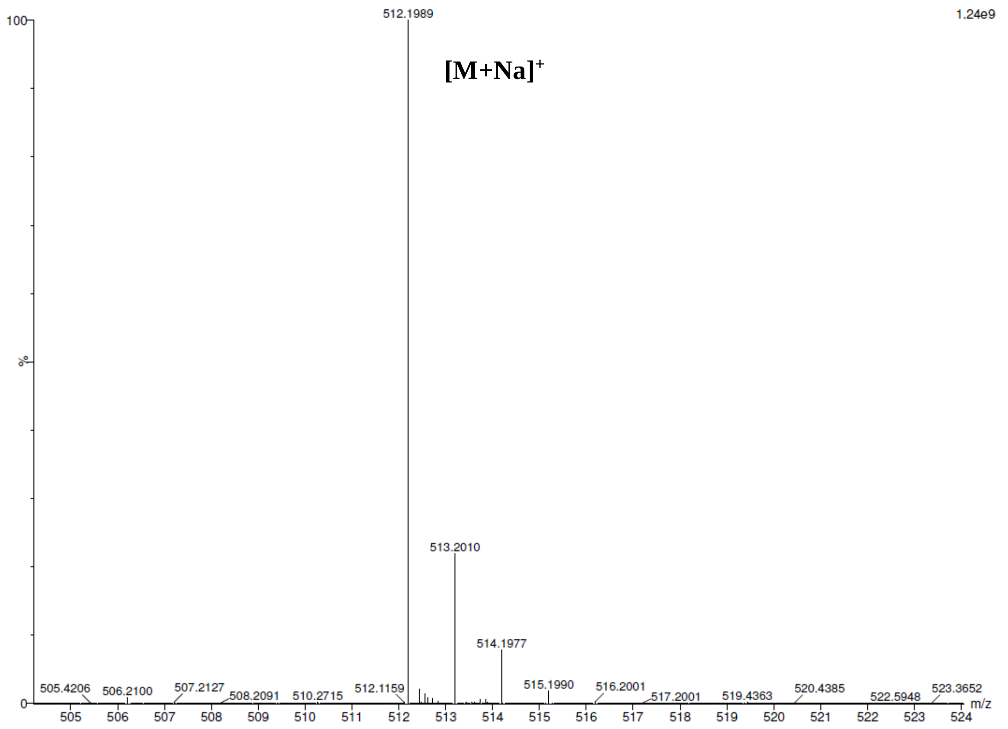

Figure S60. Partial HRMS (ESI$^+$) spectrum of **9**.

Compound 11

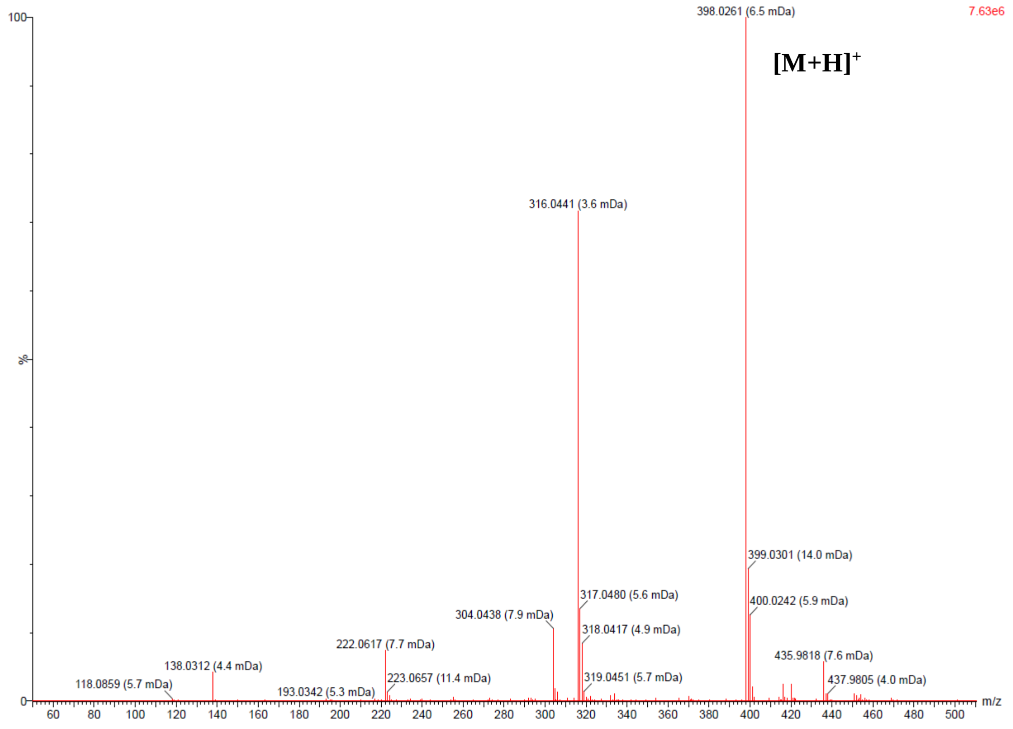

Figure S61. HRMS (ESI$^+$) spectrum of **11** (performed using 0.1% of formic acid).

Compound 13

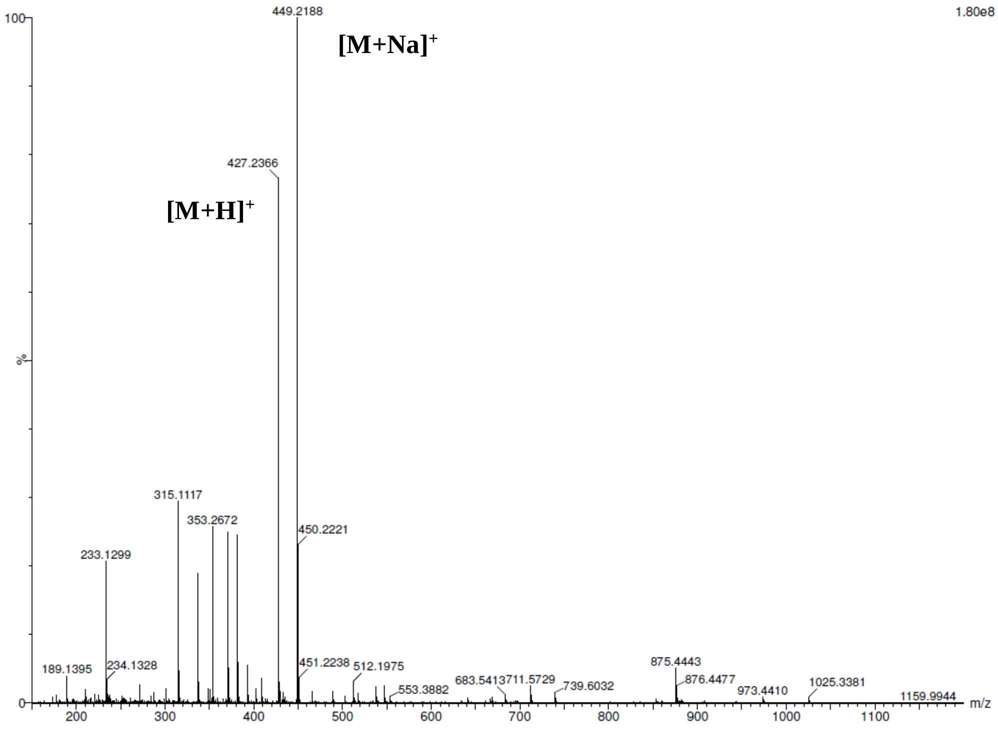

Figure S62. HRMS (ESI$^+$) spectrum of **13**.

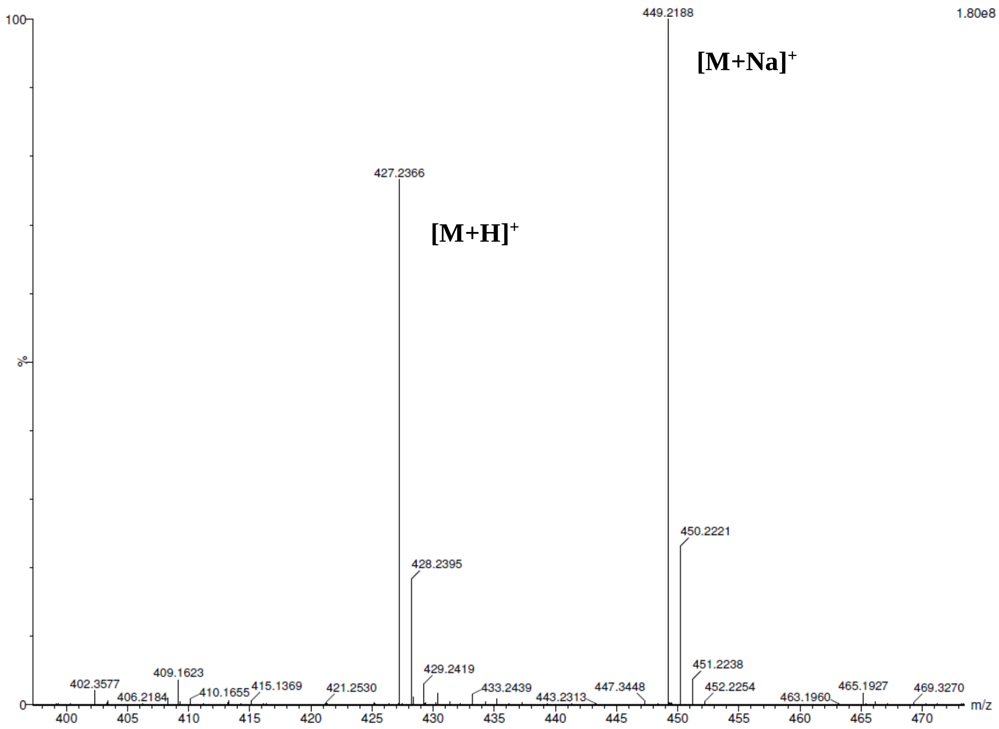

Figure S63. Partial HRMS (ESI$^+$) spectrum of **13**.

Compound 14

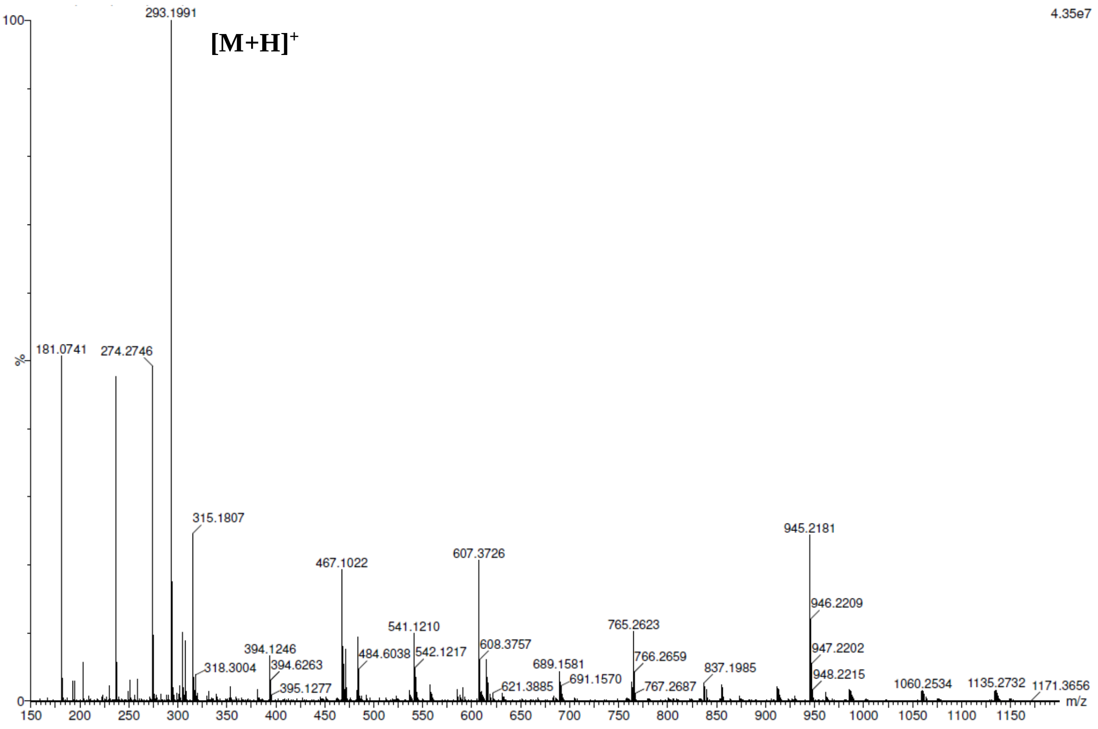

Figure S64. HRMS (ESI+) spectrum of **14**.

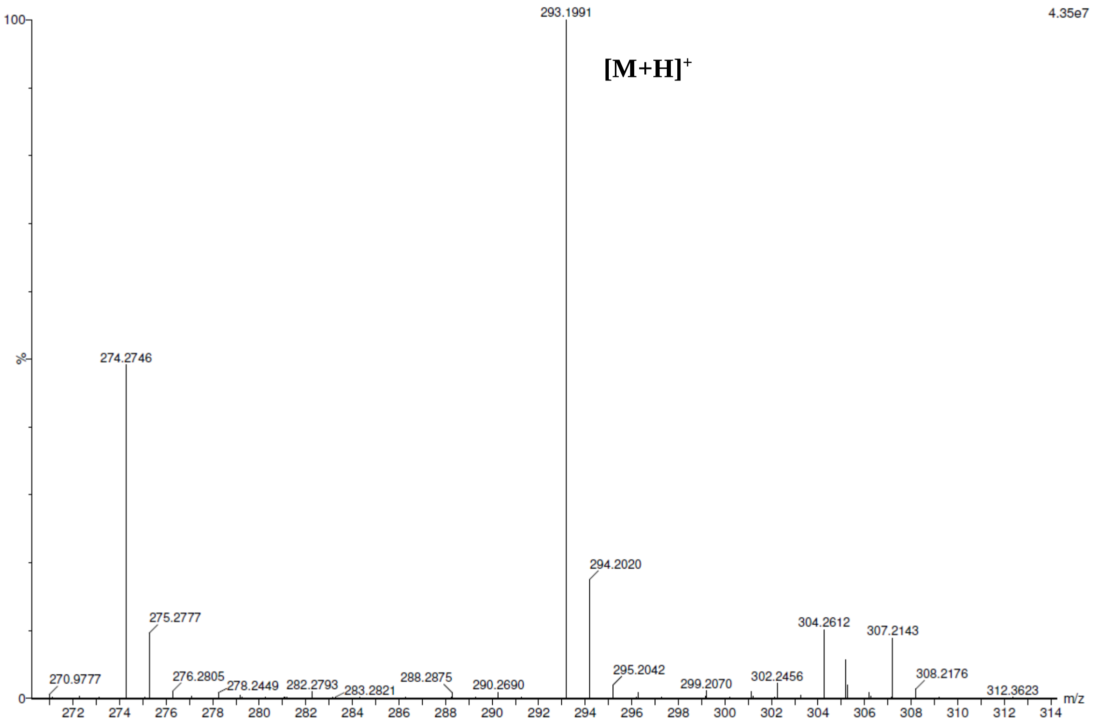

Figure S65. Partial HRMS (ESI+) spectrum of **14**.

Compound 16

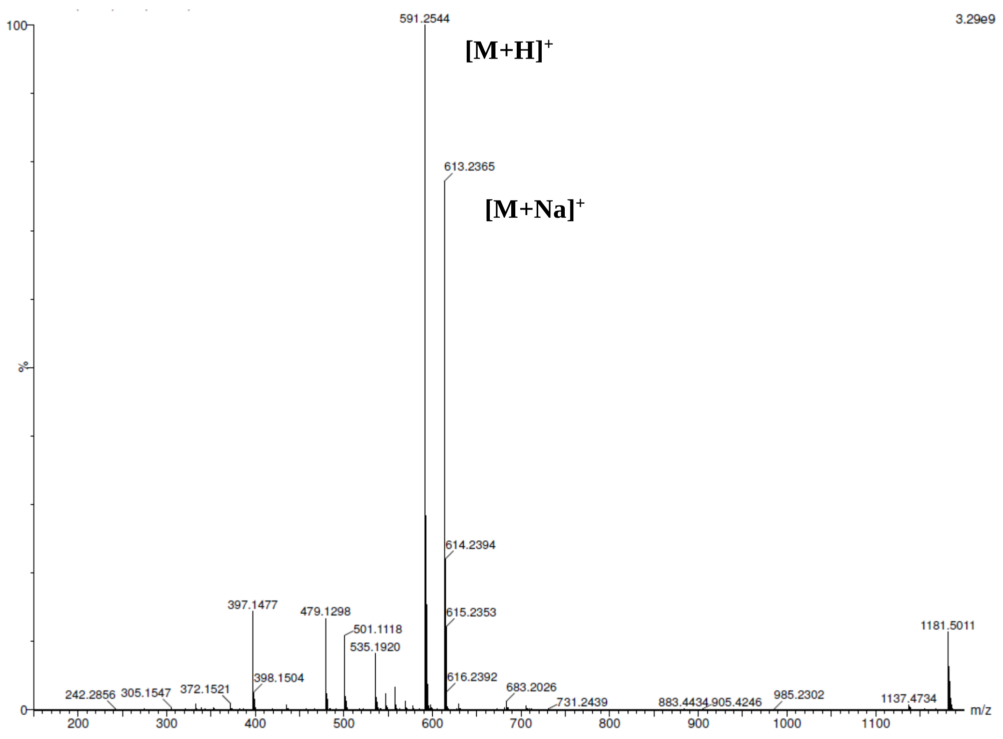

Figure S66. HRMS (ESI$^+$) spectrum of **16**.

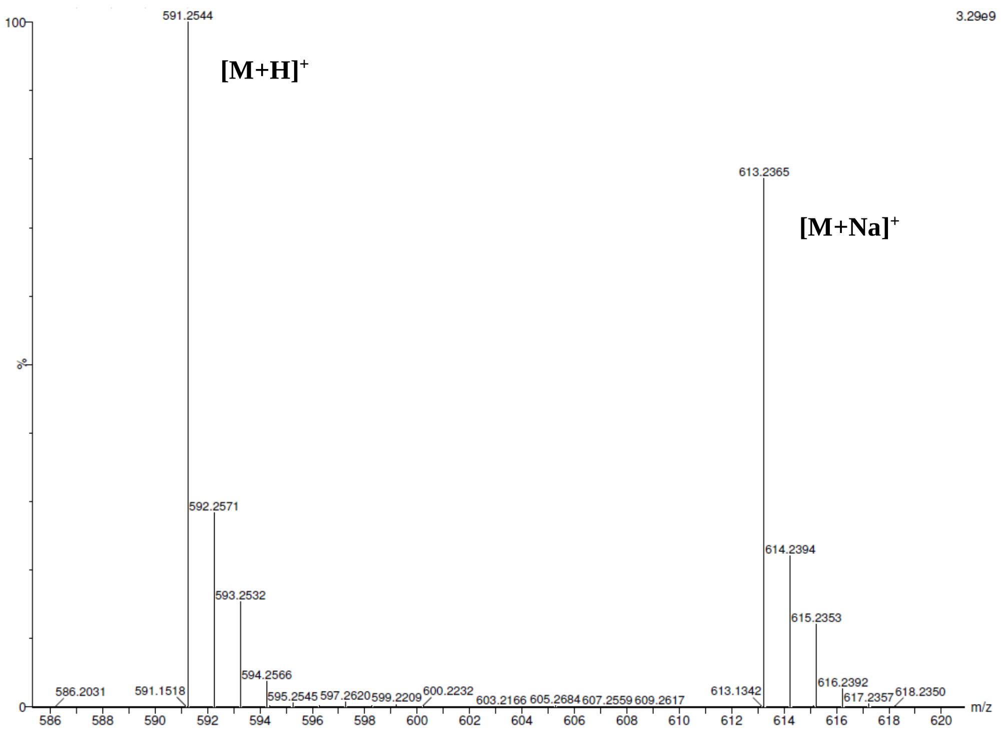

Figure S67. Partial HRMS (ESI$^+$) spectrum of **16**.

Compound 17

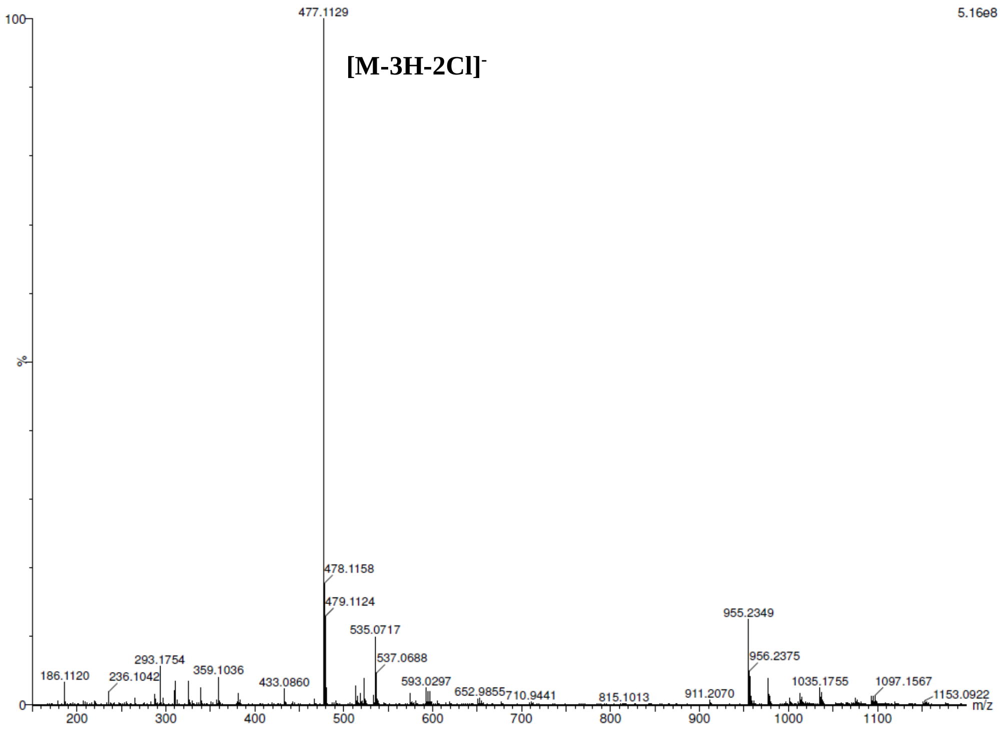

Figure S68. HRMS (ESI$^-$) spectrum of **17**.

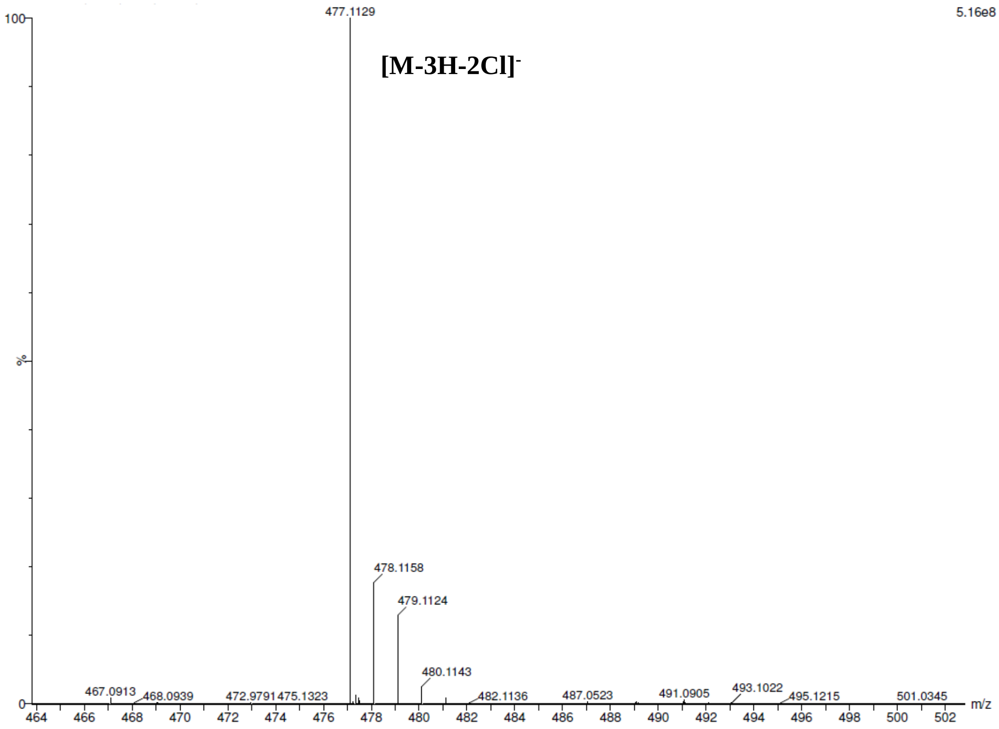

Figure S69. Partial HRMS (ESI$^-$) spectrum of **17**.

5. Particle size of the PEI-BP-CD ternary conjugates

Table S2. Particle size of the PEI-BP-CD ternary conjugates

Nanoparticle	Size (nm)	SEM
**20a**	445.9	55.6
**20b**	450.9	15.8
**21a**	533.0	45.6
**21b**	402.8	29.4
**22a**	529.8	22.1
**22b**	560.3	27.5

The nanoparticle size was determined using the Zetasizer µV instrument (Malvern) and 50 µl UV-transparent Disposable cuvettes (Sarsted). A 0.1 mg/ml suspension of nanoparticles (PEI-βCD or PEI-BP derivatives) was prepared in PBS and the measurement was carried out at 25°C with a refractive index of the sample of 1.53 and A=0, with 3 cycles of 15 measurements of 10 s.

6. Subcellular fractionation of MG-63 cells

Table S3. Subcellular fractionation of MG-63 cells

	Pyruvate Carboxylase		Histone H3	
**Fraction**	**DOX**	**DOX⊂21a**	**DOX**	**DOX⊂21a**
**Lysate**	100.00 ± 6.68	100.00 ± 10.31	100.00 ± 16.10	100.80 ± 12.80
**Cytosol**	31.43 ± 9.54	42.73 ± 1.43	16.04 ± 4.54	11.49 ± 0.28
**Nuclei**	123.40 ± 6.00	84.81 ± 33.64	109.13 ± 15.51	95.67 ± 5.18
**Mitochondria**	210.73 ± 21.46	210.94 ± 8.38	9.72 ± 2.90	7.61 ± 3.83

Mitochondria from MG-63 cells treated with DOX or **DOX⊂21a** for two hours were isolated by a differential centrifugation method. Briefly, cells were washed and scraped with 1.5 ml of sterile ice-cold phosphate-buffered saline (PBS, Sigma-Aldrich, Missouri, USA) and centrifuged 10 min at 800 x g. The pellet was homogenized with a mitochondrial isolation buffer (Tris HCl 50 mM pH 7.5; sucrose 250mM, EDTA 1mM) in a Teflon-glass homogenizer at maximum speed for 10 passes. The cell lysate was centrifuged 10 min at 800 x g to remove cell debris and nuclei and then the supernatant was spun 10 min at 10 000 x g to obtain mitochondria enriched pellet and a cytosolic fraction as supernatant. All procedure was carried out in cold conditions (at 4 °C). Markers for each subcellular fraction were measured by Western-blot using an antibody against Pyruvate Carboxylase as mitochondrial marker and against Histone H3 as nuclear marker. Results are means ± S.E.M. (n=4).

7. Effects of inhibitors of internalization routes on DOX⊂21b uptake

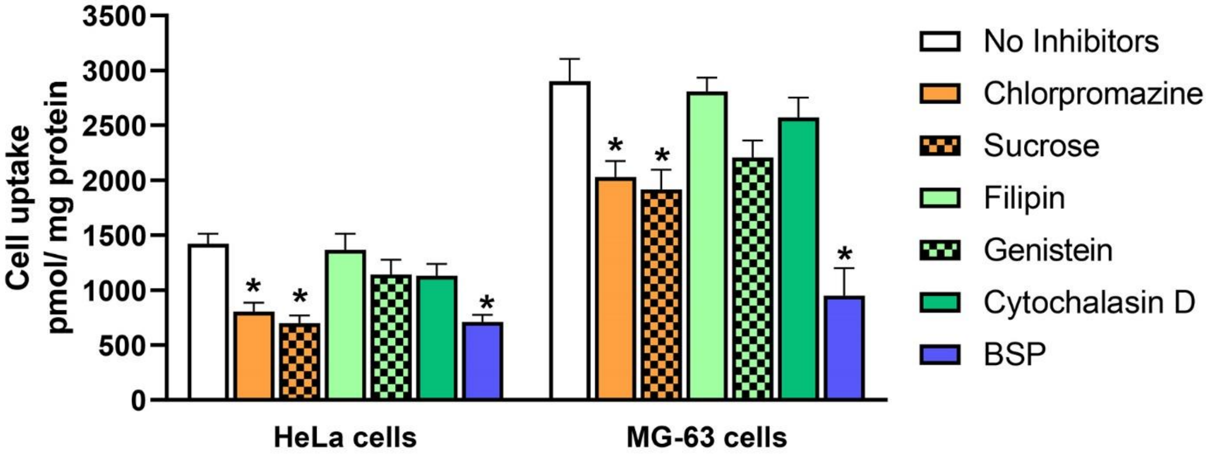

Figure S70. *Effects of inhibitors of internalization routes on* ***DOX⊂21b*** *uptake.* HeLa and MG-63 sarcoma cells were pretreated with chlorpromazine (50 µM), sucrose (0.45 M), filipin (5 µg/mL), genistein (400 µM), cytochalasin D (2 µM) or bromosulfophthalein (BSP) (0.25 mM) for 30 min before incubation with **DOX⊂21b** (1µM). DOX uptake was determined 2 h later. Results are expressed as pmol of DOX/mg protein as means ± SEM (n = 6). *p < 0.05 vs **DOX⊂21b** treated cells.

8. Specific PEI-BP-CD (20-21b) and PEI-MP-CD (22b) conjugates uptake in bone cells

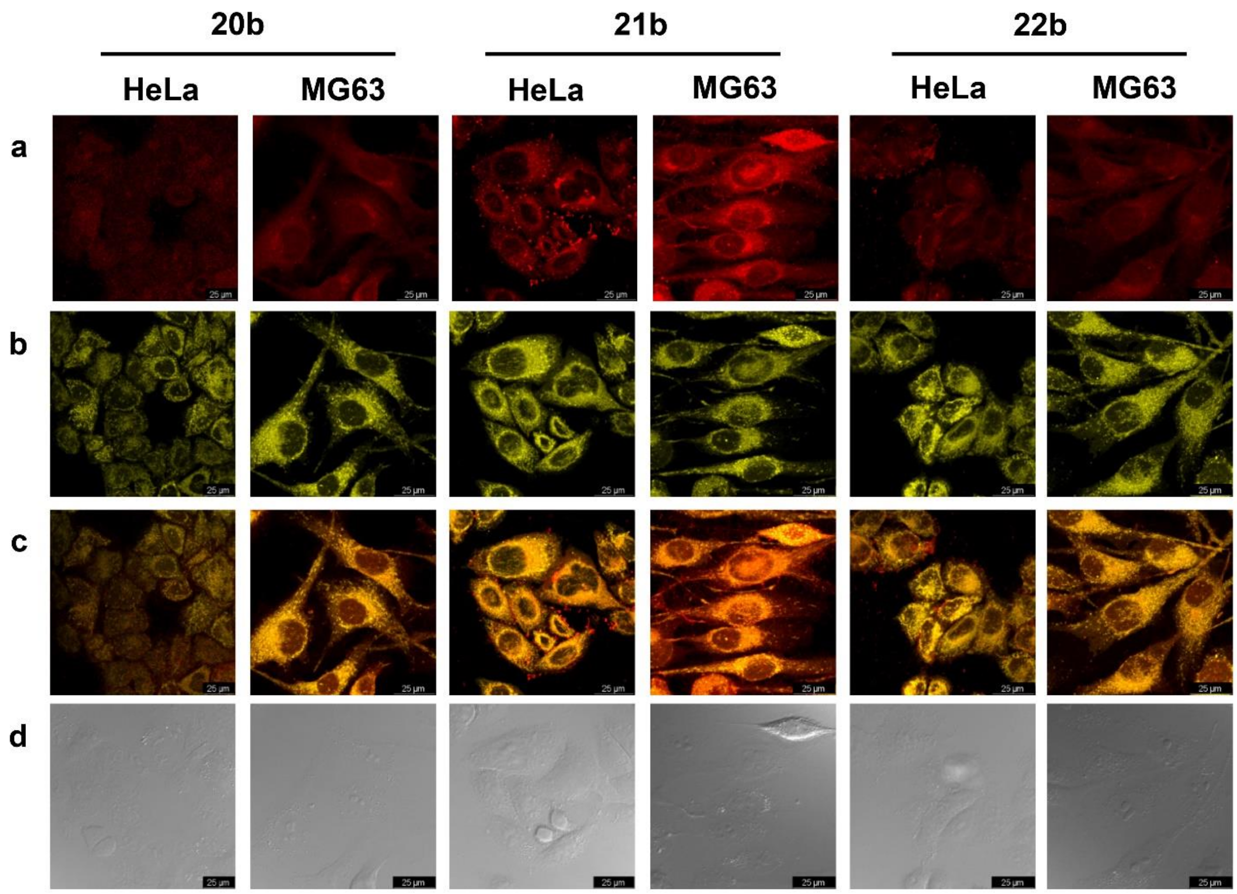

Figure S71. *Specific PEI-BP-CD (**20-21b**) and PEI-MP-CD (**22b**) conjugates.* HeLa and MG-63 sarcoma cells were incubated with mitotracker Deep Red (a) for 30 min, and then with different PEI-BP derivatives containing DOX (b) for 2 hours. Merge (c) and Nomarsky (d) photos are also depicted.

9. *In vivo* imaging of tumor MDA-MB-231 xenografts in mice

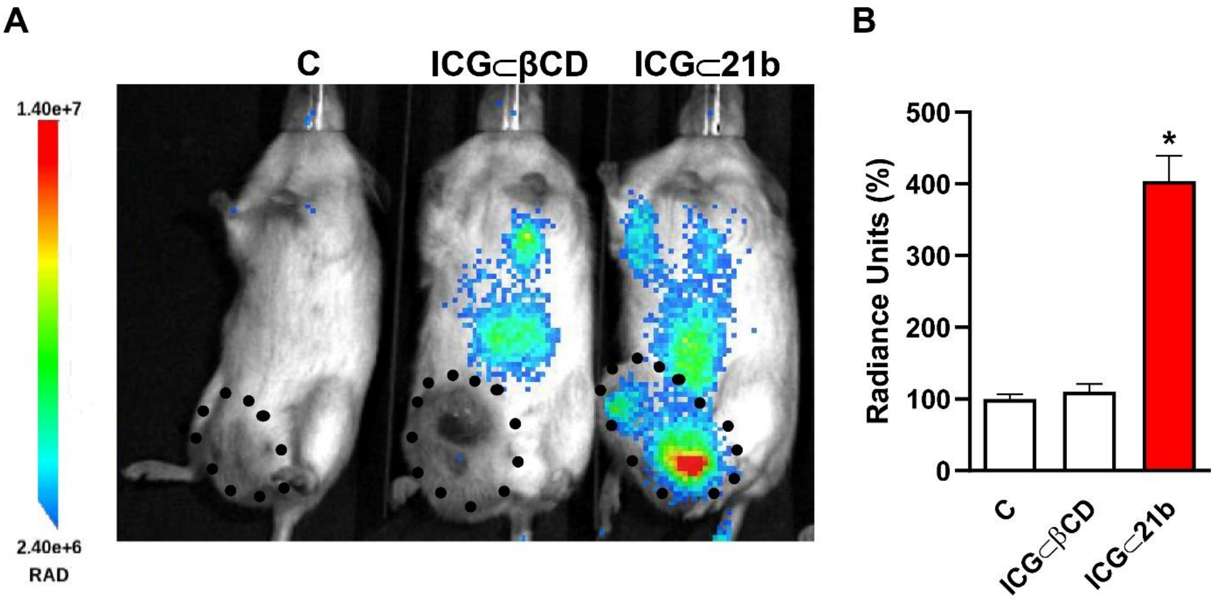

Figure S72. *In vivo imaging of tumor MDA-MB-231 xenografts in mice.* NSG mice bearing breast cancer (MDA-MB-231 cells) tumors were injected intravenously in the tail vein with IGC⊂βCD or **IGC⊂21b** and fluorescence was measured 30 min later. (A) ICG fluorescence images. The size of the xenografts is indicated by a dotted line. (B) Average radiance of the xenografts in vivo. Data are shown as means ± SEM (n = 4). *p < 0.05 vs C animals.
